# 3-Benzylaminomethyl Lithocholic Acid Derivatives Exhibited Potent and Selective Uncompetitive Inhibitory Activity Against Protein Tyrosine Phosphatase 1B (PTP1B)

**DOI:** 10.1021/acsomega.4c04948

**Published:** 2024-07-22

**Authors:** María-Eugenia Mendoza-Jasso, Jaime Pérez-Villanueva, José G. Alvarado-Rodríguez, Martin González-Andrade, Francisco Cortés-Benítez

**Affiliations:** † Doctorado en Ciencias Farmacéuticas, División de Ciencias Biológicas y de la Salud, Universidad Autónoma Metropolitana − Unidad Xochimilco, Ciudad de México 04960, Mexico; ‡ Laboratorio de Síntesis y Aislamiento de Sustancias Bioactivas, Departamento de Sistemas Biológicos, División de Ciencias Biológicas y de la Salud, Universidad Autónoma Metropolitana − Unidad Xochimilco, Ciudad de México 04960, Mexico; § Laboratorio de Biosensores y Modelaje Molecular, Departamento de Bioquímica, Facultad de Medicina, Universidad Nacional Autónoma de México, Ciudad de México 04510, Mexico; ∥ Área académica de Química, Universidad Autónoma del Estado de Hidalgo, Hidalgo 42184, Mexico

## Abstract

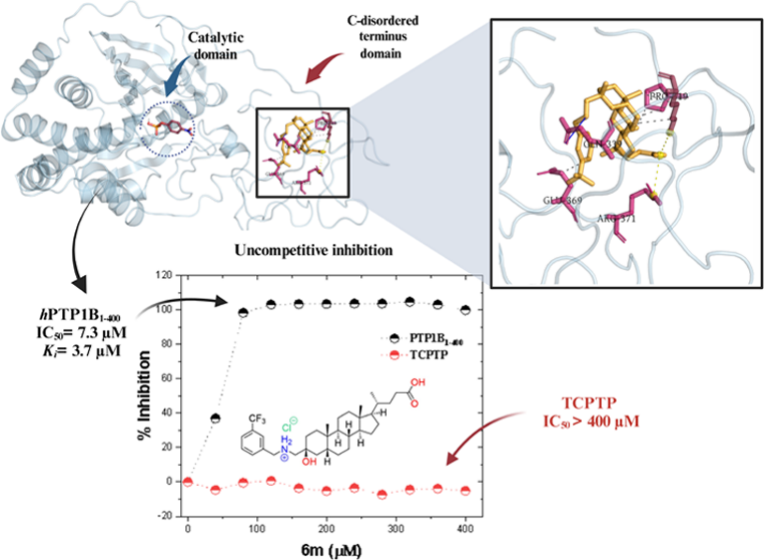

Protein tyrosine phosphatase 1B (PTP1B) is a promising drug target for treating type 2 diabetes (T2DM) and obesity. As a result, developing new therapies that target PTP1B is an attractive strategy for treating these diseases. Herein, we detail the synthesis of 15 lithocholic acid (LA) derivatives, each containing different benzylaminomethyl groups attached to the C3 position of the steroid skeleton. The derivatives were assessed against two forms of PTP1B enzyme (*h*PTP1B_1–400_ and *h*PTP1B_1–285_), and the most potent compounds were then tested against T-cell protein tyrosine phosphatase (TCPTP) to determine their selectivity. The results showed that compounds **6m** and **6n** were more potent than the reference compounds (ursolic acid, chlorogenic acid, suramin, and TCS401). Additionally, both compounds exhibited greater potency over *h*PTP1B_1–400_. Furthermore, enzyme kinetic studies on *h*PTP1B_1–400_ revealed that these two lithocholic acid derivatives have an uncompetitive inhibition against *h*PTP1B_1–400_ with *K*_i_ values of 2.5 and 3.4 μM, respectively. Interestingly, these compounds were around 75-fold more selective for PTP1B over TCPTP. Finally, docking studies and molecular dynamics simulations (MDS) were conducted to determine how these compounds interact with PTP1B. The docking studies revealed hydrophobic and H-bond interactions with amino acid residues in the unstructured region. MDS showed that these interactions persisted throughout the 200 ns simulation, indicating the crucial role of the unstructured zone in the biological activity and inhibition of PTP1B.

## 1 Introduction

The World Health Organization (WHO) describes diabetes mellitus (DM) as a chronic metabolic disease characterized by elevated blood glucose levels. Type 2 diabetes (T2DM) is the most common and diagnosed worldwide. It occurs when the body becomes resistant to insulin or does not produce enough insulin.^1^ Prolonged exposure to high glucose blood levels leads to severe, irreversible damage to the eyes, heart, kidneys, and nerves, including coronary heart disease, chronic kidney disease, peripheral neuropathy, vascular disease, oral disorders, and retinopathy. Moreover, other serious pathologies have been associated with DM, such as an increased risk of developing cancer, liver disease, infection-related complications, and cognitive and affective disorders.^1,2^

Some targets such as free fatty acid receptor 1 (FFAR1), α-glucosidase, peroxisome proliferator-activated receptor-γ (PPARγ), dipeptidyl peptidase-4 (DPP4), sodium–glucose cotransporter 2 (SGLT2), aldose reductase (ALR), glycogen phosphorylase (GP), fructose-1,6-bisphosphatase (FBPase), glucagon receptor (GCGr), phosphoenolpyruvate carboxykinase (PEPCK), and protein tyrosine phosphatase 1B (PTP1B) have been identified to treat DM.^2,3^

PTP1B is a negative insulin and leptin signaling pathway regulator, and a validated therapeutic target for DM and obesity. PTP1B-knockout mice exhibited improved insulin sensitivity and resistance to gaining weight with much-lowered triglyceride levels.^4−7^ PTP1B catalyzes either the dephosphorylation of phosphotyrosine (pTyr) residues of activated insulin receptor subunit β (IRb) and insulin receptor substrate-1 and 2 (IRS-1, IRS-2), or the dephosphorylation of the leptin receptor (LepR) and Janus kinase (JAK2), inactivating STAT3, and controlling the expression of genes *POMC* and *SOCS3* involved in energy balance.^3,8,9^ PTP1B has been identified as a potential target for treating both DM and obesity for a long time. However, in recent years, it has gained more attention for its role in the development of various other diseases, including Alzheimer’s, Parkinson’s, metabolic dysfunction-associated steatotic liver disease (MASLD), cardiac dysfunction, and certain types of cancer such as breast, prostate, and pancreatic cancer.^9,10^ Hence, developing novel therapies that can effectively target this enzyme has become a subject of significant interest. However, developing potent and selective PTP1B inhibitors is challenging because of the high homology between PTP1B and T-cell protein tyrosine phosphatase (TCPTP) catalytic domain (∼74%).^7,11^ TCPTP is highly expressed in hematopoietic cells, and inhibiting it causes defects in hematopoiesis and immune function caused by B- and T-cell abnormalities.^12^ Therefore, the combined inhibition of TCPTP and PTP1B may have several adverse effects.^5^ Therefore, developing selective inhibitors of PTP1B would be an excellent strategy to reduce the side effects of inhibiting both enzymes. In this regard, natural products are known to be essential sources for drug discovery. Many natural products, such as alkaloids, flavonoids, triterpenoids, and steroids, have been described as PTP1B inhibitors.^13^

The steroid lithocholic acid (LA) is a monohydroxy bile acid and metabolic product by bacterial 7-dehydroxylation of chenodeoxycholic acid (CDCA) and ursodeoxycholic acid (UDCA).^14^ Researchers have developed some synthetic methods for LA using various starting materials, including animal bile-based materials such as cholic acid, deoxycholic acid, chenodeoxycholic acid, hyodeoxycholic acid, and plant-based bisnoralcohol. The overall yield of the second method ranges from 60% to 70%.^15,16^ Therapeutic functions of LA have been studied, such as antimicrobial,^17,18^ tumor inhibition,^19,20^ and vitamin D receptor modulation.^21,22^ LA has been reported as a PTP1B inhibitor with an IC_50_ value of 12.54 μM and 2-fold more selectivity for PTP1B over TCPTP.^23^ Despite that, few attempts to prepare semisynthetic derivatives have been reported. In this regard, He et al.^23^ reported the synthesis of 4,4-dimethyl lithocholic acid derivatives with fused *N*-phenylpyrazoles moieties to the A-ring. These compounds were potent competitive inhibitors of PTP1B with IC_50_ values up to 0.73 μM and 32-fold more selectivity for PTP1B over TCPTP. The results of docking studies determined meaningful H-bond interactions with the guanidine group of Arg^254^ to the second pTyr binding site of PTP1B, while the COOH group bound into the catalytic site for these compounds.^5,23^

To enhance the potency and selectivity of LA as a PTP1B inhibitor, herein, we report the synthesis, biological evaluation, molecular docking analyses, and molecular dynamics simulations (MDS) of 15 3-benzylaminomethyl lithocholic acid derivatives. We based our design on the fact that LA has been reported as a competitive inhibitor. We propose the addition of different benzylamines to C3 to mimic the pTyr substrate, as well as the incorporation of electronegative groups such as OCH_3_, OH, and COOH, F, Cl, CF_3_ in the *para* and *meta* positions of the benzene ring of the LA derivatives. This can increase its affinity for PTP1B due to the positively charged nature of the catalytic domain of the enzyme,^24,25^ which is composed of residues Tyr^46^, Asp^48^, Lys^120^, Asp^181^, Phe^182^, Cys^215^, Ser^216^, Ile^219^, Arg^221^, and Gln^262^, leading to enhanced inhibitory activity. Also, it is proposed to investigate the impact on the inhibition of PTP1B by adding hydrophobic groups (CH_3_ and CF_3_).

**Figure 1:**
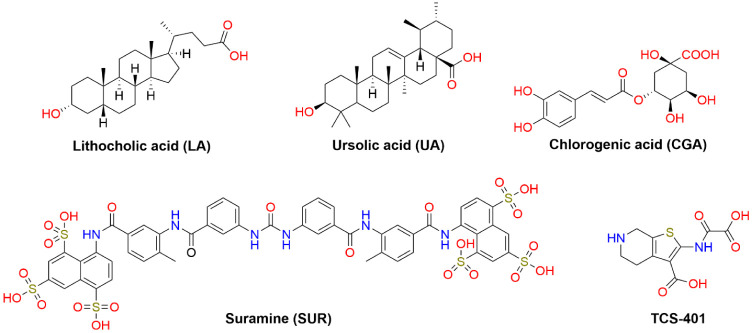
Structure of PTP1B inhibitors.

The new LA derivatives (**6a**–**6o**) were tested at several concentrations against PTP1B. Their inhibitory activity was compared to the reference compounds LA, ursolic acid (UA), suramin (SU), chlorogenic acid (CGA), and TCS-401 (Figure 1). UA is a pentacyclic triterpenoid found in various plants and possesses a wide range of biological functions. It is implicated in reducing blood glucose, and has been identified as a PTP1B inhibitor.^26^ Suramin is used to treat sleeping sickness and onchocerciasis. Moreover, it has shown potent competitive PTP1B inhibition activity.^27,28^ CGA is a phenolic acid with various biological functions, including antioxidant, antibacterial, hepatoprotective, cardioprotective, anti-inflammatory, antipyretic, neuroprotective, antiobesity, and central nervous system (CNS) stimulator. In addition, CGA can regulate lipid metabolism and glucose levels.^29^ It has been reported as a competitive PTP1B inhibitor.^3^ Finally, TCS401, a PTP1B competitive inhibitor, inhibits glucagon-induced insulin-mediated glycogenolysis in primary hepatocytes. It is more selective than other phosphatases involved in the negative regulation of the insulin receptor, such as PTPα and PTP-LAR.^11^

## 2 Materials and Methods

### 2.1 Synthesis

All commercial reagents and TLC silica gel plates were acquired from Sigma-Aldrich (St. Louis, MO, USA). TLC monitored reactions on 0.2 mm percolated silica gel 60 F254 plates and visualized them with a UV lamp. Column chromatography was performed on silica gel 60 (0.063–0.2 mm mesh). Melting points were determined by the Fisher–Johns apparatus and were uncorrected. NMR spectra were recorded on Agilent DD2 (Agilent, Santa Clara, CA, USA) and Bruker Ascend (Bruker, Billerica, MA, USA) spectrometers at 600 and 500 MHz, for ^1^H, respectively, and 151 and 126 MHz for ^13^C, respectively with chemical shifts reported as parts per million. TMS was used as an internal standard. Splitting patterns are expressed as follows: s: singlet; d: doublet; q: quadruplet m: multiplet. ESI-MS spectra were obtained using micrOTOF-ESI-TOF-MS mass spectrometer by direct infusion and in a positive mode using nitrogen (4 mL/min) as nebulizer gas, spray voltage (4.5 kV) at 150 °C, within a mass range of *m*/*z* 50–3000. The results are expressed as *m*/*z*. All data spectra are reported in Supporting Information. According to IUPAC rules, compounds were named using the automatic generator tool implemented in ChemDraw Professional 22.0.0 software (PerkinElmer, Waltham, MA, USA).

**Scheme 1:**
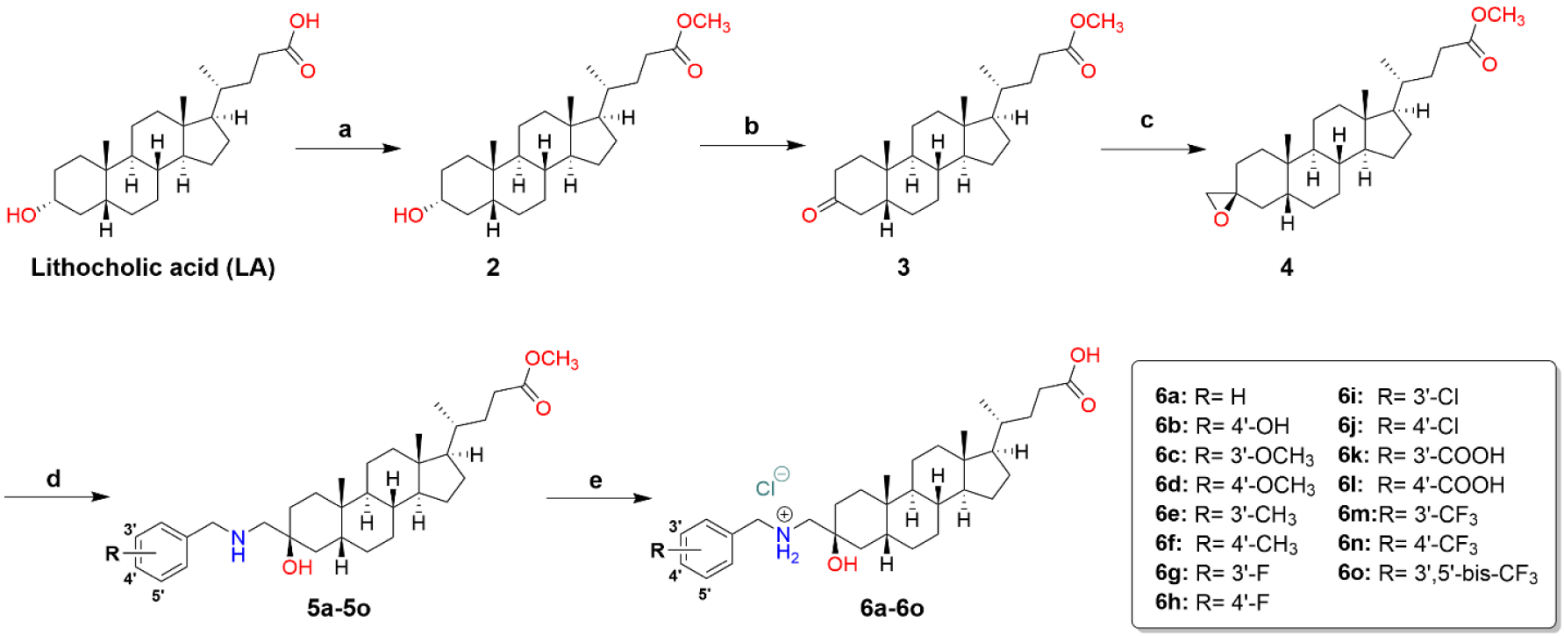
Synthesis of 3-Benzylaminomethyl Lithocholic Acid Derivatives (**6a**–**6o**) a) SOCl_2_, MeOH, 0° C, 2.5 h, 97%; b) PDC, CH_2_Cl_2_, r.t, 24 h, 90%; c) TMSI, NaH, DMSO, r.t, 18 h, 86%; d) *meta* or *para*-substituted benzylamines, EtOH, 60 °C, 18 h; e) 1. KOH, MeOH, 70 °C, 2 h; 2. HCl conc, CH_2_Cl_2_, 10-80%.

Single crystals of **4** were obtained from a CH_2_Cl_2_ solution. Data were collected using an Agilent Xcalibur Gemini CCD diffractometer using graphite-monochromated MoKa (*l* = 0.71073 Å) radiation in the ω-2θ scan mode at 293 K by using Olex2.^30^ The structures were solved with the SHELXT^31^ structure solution program using Intrinsic Phasing and refined with the SHELXL^32^ refinement package using the least-squares minimization. The non-hydrogen atoms were treated anisotropically. Hydrogen atoms included in the structure factor calculation were placed at idealized positions and refined isotropically. The absolute configuration of **4** was established by the known configuration of the lithocholic acid acquired from Sigma and used as a starting material (Scheme 1). Table S3 shows relevant crystal data. The CCDC-2354163 data have been deposited to the Cambridge Crystallographic Data Centre from where they can be obtained, free of charge, via http://www.ccdc.cam.ac.uk/conts/retrieving.html.

#### 2.1.1 Synthesis of Methyl 3α-hydroxy-5β-cholan-24-oate (**2**)

This compound was prepared according to the reference.^5^ To a solution of lithocholic acid (LA, 9.83 mmol) in methanol (100 mL), thionyl chloride (2.9 mL, 39.97 mmol) was added. The reaction was stirred at 0 °C for 30 min and then at room temperature for 2 h. The solvent was evaporated, and water was added. The precipitate was filtered and dried to give **2** (97%). The crude product was used for the following reaction without any further purification.

#### 2.1.2 Synthesis of Methyl 3-oxo-5β-cholan-24-oate (**3**)

Pyridinium dichromate (19.3 mmol) and 1 g of silica gel were added to a solution of **2** (10.05 mmol) in 150 mL of dichloromethane. The reaction was stirred at room temperature for 24 h. Afterward, the solvent was evaporated, and the crude product was purified by column chromatography using hexanes/EtOAc (85:15) to give the titled compound as a white solid. Yield 90%. Mp. 117–118 °C. ^1^H NMR (600 MHz, CDCl_3_) δ 3.67 (s, 3H), 2.79–2.62 (m, 1H), 1.02 (s, 3H), 0.92 (d, *J* = 6.5 Hz, 3H), 0.68 (s, 3H). ^13^C NMR (151 MHz, CDCl_3_) δ 213.82, 175.07, 56.72, 56.24, 51.84, 21.50, 18.59, 12.38.

#### 2.1.3 Synthesis of Methyl (3β-oxirane)-5β-cholan-24-oate (**4**)

**Figure 2:**
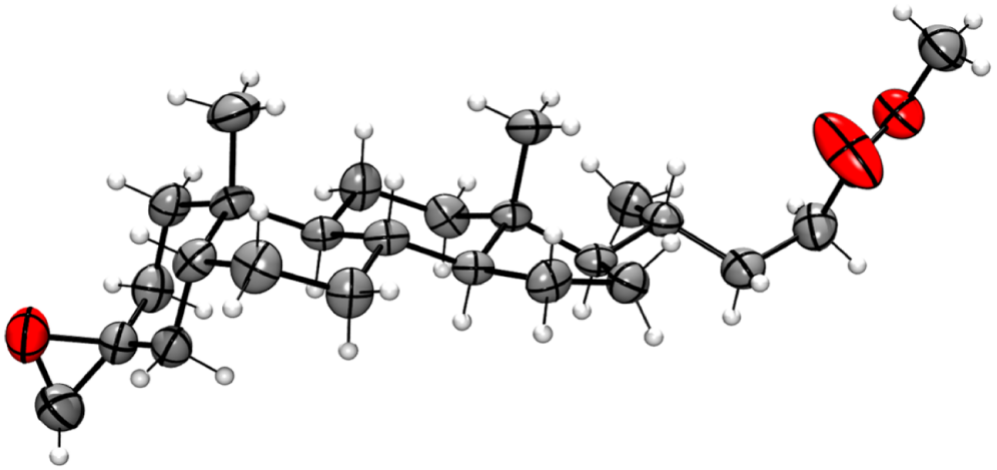
ORTEP view of methyl (3β-oxirane)-5β-cholan-24-oate (**4**).

Sodium hydride 60% in oil (4.2 mmol) was added to a solution of trimethylsulfoxonium iodide (1.93 mmol) in 5 mL of anhydrous DMSO. The mixture was stirred under nitrogen atmosphere for 1 h at room temperature. Then, a solution of **3** (1.29 mmol) in 3 mL of DMSO was slowly added. The reaction mixture was stirred for 18 h, poured into a water/ice bath (150 mL), filtered, and dried. The crude product was purified by column chromatography using hexanes/EtOAc (90:10) to give the oxirane **4** as a white solid. Yield 86%. Mp. 132 °C. ^1^H NMR (600 MHz, CDCl_3_) δ 3.67 (s, 3H), 2.62 (d, *J* = 1.2 Hz, 2H), 0.99 (s, 3H), 0.92 (d, *J* = 6.5 Hz, 3H), 0.66 (s, 3H). ^13^C NMR (151 MHz, CDCl_3_) δ 174.90, 59.39, 56.72, 56.11, 53.74, 51.63, 21.22, 18.39, 12.18. HRMS (ESI-MS) *m*/*z* for C_26_H_43_O_3_ [M + H]^+^ calc. 403.3207, found 403.3207. Single crystal X-ray diffraction: see Figure 2 and Table S3.

#### 2.1.4 General Procedure for the Synthesis of Intermediates **5a**–**5o**

A solution of oxirane **4** (0.371 mmol) in ethanol (5 mL) was added to the appropriate *para-* or *meta-*benzylamine (1.112 mmol), and the solution was stirred for 22 h at 60 °C. Afterward, the resulting mixture was evaporated, and the crude products were purified by column chromatography using hexanes/EtOAc (90:10 to 85:15) to give the corresponding secondary amines (**5a**–**5o**). DMF was used as the solvent for compounds **5k** and **5l**. The reaction mixture was stirred at 90 °C for 22 h.

#### 2.1.5 General Procedure for the Synthesis of *N*-Benzyl Methylammonium Chloride Derivatives of Lithocholic Acid (**6a**–**6o**)

Potassium hydroxide (1.71 mmol) was added to a solution of the compounds **5a**–**5o** (1.112 mmol) in 5 mL of methanol. The reaction mixture was stirred at 70 °C overnight. After the solvent was evaporated, a mixture of concentrated HCl (1 mL) and dichloromethane (10 mL) was added. The resulting solid was filtered and dried, giving compounds **6a**–**6o**.

##### 2.1.5.1 *N*-Benzyl-1-((3*S*,5*R*,10*S*,13*R*)-17-((*R*)-4-carboxybutan-2-yl)-3-hydroxy-10,13-dimethylhexadecahydro-1*H*-cyclopenta[a]phenanthren-3-yl)methanaminium Chloride (**6a**)

White solid. Yield 37%. Mp. 262–266 °C. ^1^H NMR (600 MHz, pyridine-*d*_5_) δ 7.96 (*d*, *J* = 7.3 Hz, 2H), 7.33 (t, *J* = 7.1 Hz, 2H), 7.30 (d, *J* = 6.8 Hz, 1H), 4.66 (s, 2H), 3.67 (s, 1H), 3.38 (q, *J* = 12.6 Hz, 2H), 0.97 (d, *J* = 6.3 Hz, 3H), 0.94 (s, 3H), 0.58 (s, 3H). ^13^C NMR (151 MHz, pyridine-*d*_5_) δ 176.71, 132.97, 131.08, 129.43, 129.39, 70.36, 58.83, 56.45, 52.28, 43.01, 40.04, 38.31, 35.95, 35.86, 35.15, 32.05, 31.99, 31.78, 31.02, 27.18, 26.74, 23.98, 18.77, 12.38. HRMS (ESI-MS) *m*/*z* for C_32_H_50_NO_3_^+^ [M + H]^+^ calc. 496.3786; found 496.3783.

##### 2.1.5.2 1-((3*S*,5*R*,10*S*,13*R*)-17-((*R*)-4-carboxybutan-2-yl)-3-hydroxy-10,13-dimethylhexadecahydro-1*H*-cyclopenta[a]phenanthren-3-yl)-*N*-(4-hydroxybenzyl)methanaminium Chloride (**6b**)

White solid. Yield 32%. Mp. 215–218 °C. ^1^H NMR (600 MHz, DMSO-*d*_6_) δ 12.01 (s, 1H), 9.81 (s, 1H), 8.93 (d, *J* = 56.6 Hz, 2H), 7.36 (d, *J* = 8.5 Hz, 1H), 7.33 (d, *J* = 8.5 Hz, 1H), 6.80 (d, *J* = 8.5 Hz, 1H), 4.83 (s, 2H), 3.98 (t, *J* = 4.5 Hz, 2H), 2.68 (s, 2H), 0.88 (s, 3H), 0.85 (d, *J* = 6.5 Hz, 3H), 0.59 (s, 3H). ^13^C NMR (151 MHz, DMSO-*d*_6_) δ 174.90, 158.13, 132.09, 121.33, 115.42, 68.92, 56.54, 55.85, 55.70, 52.83, 50.51, 42.36, 38.87, 37.04, 35.27, 34.92, 34.33, 30.84, 30.79, 23.45, 18.26, 11.96. HRMS (ESI-MS) *m*/*z* for C_32_H_50_NO_4_^+^ [M + H]^+^ calc. 512.3735; found 512.3727.

##### 2.1.5.3 1-((3*S*,5*R*,10*S*,13*R*)-17-((*R*)-4-carboxybutan-2-yl)-3-hydroxy-10,13-dimethylhexadecahydro-1*H*-cyclopenta[a]phenanthren-3-yl)-*N*-(3-methoxybenzyl)methanaminium Chloride (**6c**)

White solid. Yield 51%. Mp. 243–246 °C. ^1^H NMR (600 MHz, pyridine-*d*_5_) δ 7.85 (s, 1H), 7.59 (s, 1H), 7.45 (d, *J* = 7.4 Hz, 1H), 7.28 (d, *J* = 1.5 Hz, 1H), 6.96 (d, *J* = 8.2 Hz, 1H), 4.69 (s, 3H), 3.76 (d, *J* = 2.3 Hz, 3H), 3.43 (q, *J*= 12.6, 2H), 2.61 (m, 2H), 0.98 (d, *J* = 4.9 Hz, 3H), 0.95 (s, 3H), 0.58 (s, 3H). ^13^C NMR (151 MHz, pyridine-*d*_5_) δ 177.05, 161.01, 134.56, 130.75, 123.39, 116.58, 116.07, 70.70, 59.07, 56.76, 55.99, 52.58, 43.34, 40.37, 36.29, 36.19, 35.48, 32.39, 32.32, 24.32, 19.10, 12.71. HRMS (ESI-MS) *m*/*z* for C_33_H_52_NO_4_^+^ [M + H]^+^ calc. 526.3891; found 526.3896.

##### 2.1.5.4 1-((3*S*,5*R*,10*S*,13*R*)-17-((*R*)-4-carboxybutan-2-yl)-3-hydroxy-10,13-dimethylhexadecahydro-1*H*-cyclopenta[a]phenanthren-3-yl)-*N*-(4-methoxybenzyl)methanaminium Chloride (**6d**)

White solid. Yield 44%. Mp. 132–135 °C. ^1^H NMR (600 MHz, pyridine-*d*_5_) δ 7.92 (*d*, *J* = 8.0 Hz, 2H), 6.93 (d, *J* = 8.0 Hz, 2H), 4.67 (s, 2H), 3.62 (s, 3H), 3.42 (q, *J* = 12.6 Hz, 2H), 2.66 (m, 2H), 0.98 (d, *J* = 6.1 Hz, 3H), 0.95 (s, 3H), 0.58 (s, 3H). ^13^C NMR (151 MHz, CDCl_3_) δ 176.87, 160.92, 132.96, 124.49, 114.99, 70.49, 58.51, 56.60, 55.50, 51.82, 43.17, 40.20, 38.47, 36.12, 36.02, 35.32, 32.21, 32.15, 28.74, 27.36, 26.91, 24.15, 18.93, 12.54. HRMS (ESI-MS) *m*/*z* for C_33_H_52_NO_4_^+^ [M + H]^+^ calc. 526.3891; found 526.3902.

##### 2.1.5.5 1-((3*S*,5*R*,10*S*,13*R*)-17-((*R*)-4-carboxybutan-2-yl)-3-hydroxy-10,13-dimethylhexadecahydro-1*H*-cyclopenta[a]phenanthren-3-yl)-*N*-(3-methylbenzyl)methanaminium Chloride (**6e**)

White solid. Yield 25%. Mp. 249–250 °C. ^1^H NMR (500 MHz, DMSO-*d*_6_) δ 12.13 (s, 1H), 9.22 (s, 1H), 7.57 (d, *J* = 7.7 Hz, 2H), 7.48 (t, *J* = 7.4 Hz, 1H), 7.39 (d, *J* = 7.2 Hz, 1H), 5.03 (s, 1H), 4.25 (s, 2H), 4.13 (s, 1H), 2.91 (s, 2H), 2.68 (s, 3H), 2.35 (m, 2H), 1.07 (s, 3H), 1.03 (d, *J* = 6.3 Hz, 3H), 0.78 (s, 3H). ^13^C NMR (126 MHz, DMSO-*d*_6_) δ 174.81, 137.73, 137.26, 131.39, 130.97, 129.49, 128.49, 127.43, 125.94, 68.85, 56.91, 55.78, 55.63, 50.82, 39.52, 36.93, 35.15, 34.79, 30.73, 30.68, 23.31, 20.96, 20.65, 18.14, 11.83. HRMS (ESI-MS) *m*/*z* for C_33_H_52_NO_3_^+^ [M + H]^+^ calc. 510.3942; found 510.3943.

##### 2.1.5.6 1-((3*S*,5*R*,10*S*,13*R*)-17-((*R*)-4-carboxybutan-2-yl)-3-hydroxy-10,13-dimethylhexadecahydro-1*H*-cyclopenta[a]phenanthren-3-yl)-*N*-(4-methylbenzyl)methanaminium Chloride (**6f**)

White solid. Yield 22%. Mp. 255 °C. ^1^H NMR (500 MHz, DMSO-*d*_6_) δ 11.92 (s, 1H), 9.02 (s, 1H), 7.46 (d, *J* = 8.0 Hz, 2H), 7.22 (d, *J* = 7.8 Hz, 2H), 4.83 (s, 1H), 4.07 (s, 2H), 2.70 (s, 2H), 2.31 (s, 3H), 2.15 (m, 2H), 0.89 (s, 3H), 0.85 (d, *J* = 6.5 Hz, 3H), 0.60 (s, 3H). ^13^C NMR (126 MHz, DMSO-*d*_6_) δ 174.81, 138.29, 130.60, 129.31, 128.56, 68.98, 56.85, 55.94, 55.79, 50.68, 37.09, 35.32, 34.96, 30.89, 30.85, 23.93, 23.48, 20.97, 18.31, 12.0. HRMS (ESI-MS) *m*/*z* for C_33_H_52_NO_3_^+^ [M + H]^+^ calc. 510.3942; found 510.3943. Complete NMR characterization: see Table S2.

##### 2.1.5.7 1-((3*S*,5*R*,10*S*,13*R*)-17-((*R*)-4-carboxybutan-2-yl)-3-hydroxy-10,13-dimethylhexadecahydro-1*H*-cyclopenta[a]phenanthren-3-yl)-*N*-(3-fluorobenzyl)methanaminium Chloride (**6g**)

White solid. Yield 40%. Mp > 300 °C. ^1^H NMR (600 MHz, pyridine-*d*_5_) δ 7.89 (d, *J* = 9.7 Hz, 1H), 7.71 (d, *J* = 7.5 Hz, 1H), 7.29 (m, 1H), 7.06 (t, *J* = 8.1 Hz, 1H), 5.44 (s, 2H), 4.61 (s, 2H), 3.37 (q, *J* = 12.4 Hz, 2H), 2.61 (m, 2H), 0.97 (d, *J* = 6.3 Hz, 3H), 0.94 (s, 3H), 0.58 (s, 3H). ^13^C NMR (151 MHz, pyridine-*d*_5_) δ 172.21, 157.83, 126.79, 123.71, 122.43, 113.33, 111.83, 66.0, 54.82, 51.95, 47.43, 38.49, 33.84, 31.34, 30.68, 24.33, 24.06, 22.68, 22.07, 19.99, 19.45, 16.97, 14.24, 10.80. HRMS (ESI-MS) *m*/*z* for C_32_H_49_FNO_3_^+^ [M + H]^+^ calc. 514.3691; found 514.3696.

##### 2.1.5.8 1-((3*S*,5*R*,10*S*,13*R*)-17-((*R*)-4-carboxybutan-2-yl)-3-hydroxy-10,13-dimethylhexadecahydro-1*H*-cyclopenta[a]phenanthren-3-yl)-*N*-(4-fluorobenzyl)methanaminium Chloride (**6h**)

White solid. Yield 52%. Mp. 234–236 °C. ^1^H NMR (600 MHz, DMSO-*d*_6_) δ 12.03 (s, 1H), 9.30 (d, *J* = 74.8 Hz, 1H), 7.69 (s, 1H), 7.24 (d, *J* = 1.1 Hz, 2H), 4.90 (s, 1H), 4.12 (s, 2H), 3.41 (S, 2H), 2.72 (s, 2H), 0.88 (s, 3H), 0.84 (d, *J* = 6.2 Hz, 3H), 0.58 (s, 3H). ^13^C NMR (126 MHz, DMSO-*d*_6_) δ 174.88, 161.6, 133.04, 132.98, 127.88, 115.48, 115.38, 68.93, 56.82, 55.80, 55.63, 49.89, 36.98, 35.21, 34.85, 34.25, 30.79, 30.72, 26.34, 25.85, 23.83, 23.39, 20.71, 18.19, 11.88. HRMS (ESI-MS) *m*/*z* for C_32_H_49_FNO_3_^+^ [M + H]^+^ calc. 514.3691; found 514.3696.

##### 2.1.5.9 1-((3*S*,5*R*,10*S*,13*R*)-17-((*R*)-4-carboxybutan-2-yl)-3-hydroxy-10,13-dimethylhexadecahydro-1*H*-cyclopenta[a]phenanthren-3-yl)-*N*-(3-chlorobenzyl)methanaminium Chloride (**6i**)

White solid. Yield 79%. Mp. 249–251 °C ^1^H NMR (600 MHz, pyridine-*d*_5_) δ 7.64 (s, 1H), 7.41 (s, 1H), 7.29 (s, 1H), 7.26 (d, *J* = 7.3 Hz, 1H), 5.17 (s, 1H), 3.98 (s, 2H), 2.87 (s, 2H), 1.04 (s, 3H), 1.00 (d, *J* = 3.9 Hz, 3H), 0.64 (s, 3H). ^13^C NMR (151 MHz, pyridine-*d*_5_) δ 177.55, 135.15, 131.04, 129.72,128.21, 127.99, 72.10, 57.35, 57.15, 54.73, 43.74, 41.21, 40.85, 39.29, 36.70, 36.54, 32.71, 29.27, 28.11, 27.49, 25.25, 24.88, 22.27, 19.44, 13.10. HRMS (ESI-MS) *m*/*z* for C_32_H_49_ClNO_3_^+^ [M + H]^+^ calc. 530.3396; found 530.3379.

##### 2.1.5.10 1-((3*S*,5*R*,10*S*,13*R*)-17-((*R*)-4-carboxybutan-2-yl)-3-hydroxy-10,13-dimethylhexadecahydro-1*H*-cyclopenta[a]phenanthren-3-yl)-*N*-(4-chlorobenzyl)methanaminium Chloride (**6j**)

White solid. Yield 82%. Mp. 255–256 °C. ^1^H NMR (600 MHz, pyridine-*d*_5_) δ 7.92–7.91 (d, *J* = 8.1 Hz, 2H), 7.34 (d, *J* = 8.2 Hz, 2H), 4.6 (s, 2H), 3.37 (d, *J* = 12.3 Hz, 2H), 2.65 (m, 2H), 0.98 (d, *J* = 6.2 Hz, 3H), 0.96 (s, 3H), 0.59 (s, 3H). ^13^C NMR (151 MHz, pyridine-*d*_5_) δ 177.37, 133.36, 130.07, 71.13, 59.71, 57.13, 52.33, 41.07, 40.70, 39.02, 35.50, 36.61, 36.52, 35.85, 24.65, 22.15, 19.43, 13.05. HRMS (ESI-MS) *m*/*z* for C_32_H_49_ClNO_3_^+^ [M + H]^+^ calc. 530.3396; found 530.3388.

##### 2.1.5.11 *N*-(3-carboxybenzyl)-1-((3*S*,5*R*,10*S*,13*R*)-17-((*R*)-4-carboxybutan-2-yl)-3-hydroxy-10,13-dimethylhexadecahydro-1*H*-cyclopenta[a]phenanthren-3-Yl)methanaminium Chloride (**6k**)

White solid. Yield: 22%. Mp. 220–223 °C. ^1^H NMR (600 MHz, DMSO-*d*_6_) δ 12.39 (s, 1H), 8.92 (s, 1H), 8.17 (s, 1H), 7.98–7.97 (d, *J =* 7.7 Hz, 1H), 7.83–7.82 (d, *J =* 7.6 Hz 1H), 7.56 (t, *J =* 7.7 Hz 1H), 4.79 (s, 1H), 4.2 (s, 2H), 2.77 (s, 2H), 0.9 (s, 3H) 0.86–0.85 (d, *J* = 6.4 Hz, 3H), 0.6 (s, 3H). ^13^C NMR (151 MHz, DMSO-*d*_6_) δ 174.83, 166.97, 134.94, 131.14, 129.77, 128.88, 68.84, 55.79, 55.65, 50.49, 39.52, 38.79, 36.91, 35.14, 34.90, 34.79, 34.23, 30.73, 26.29, 23.30, 18.15, 11.84. HRMS (ESI-MS) *m*/*z* for C_33_H_50_NO_5_^+^ [M + H]^+^ calc. 540.3684; found 540.3516.

##### 2.1.5.12 *N*-(4-carboxybenzyl)-1-((3*S*,5*R*,10*S*,13*R*)-17-((*R*)-4-carboxybutan-2-yl)-3-hydroxy-10,13-dimethylhexadecahydro-1*H*-cyclopenta[a]phenanthren-3-yl)methanaminium Chloride (**6l**)

White solid. Yield 39%. Mp. 231–234 °C. ^1^H NMR (600 MHz, DMSO-*d*_6_) δ 7.95–7.94 (d, *J* = 7.9 Hz, 2H), 7.62–7.61 (d, *J* = 7.9 Hz, 2H), 4.18 (s, 2H), 2.7 (s, 2H), 0.85 (s, 3H), 0.79 (d, *J* = 5.8 Hz, 3H), 0.53 (s, 3H). ^13^C NMR (151 MHz, DMSO-*d*_6_) δ 175.83, 167.66, 136.42, 131.89, 131.06, 130.12, 69.33, 57.57, 56.34, 56.06, 51.0, 42.74, 37.39, 35.62, 35.26, 34.69, 29.68, 28.19, 26.69, 26.27, 23.80, 18.59, 12.29. HRMS (ESI-MS) *m*/*z* for C_33_H_50_NO_5_^+^ [M + H]^+^ calc. 540.3684; found 540.3688.

##### 2.1.5.13 1-((3*S*,5*R*,10*S*,13*R*)-17-((*R*)-4-carboxybutan-2-yl)-3-hydroxy-10,13-dimethylhexadecahydro-1*H*-cyclopenta[a]phenanthren-3-yl)-*N*-(3-(trifluoromethyl)benzyl)methanaminium Chloride (**6m**)

White solid. Yield 26%. Mp. 248–250 °C. ^1^H NMR (600 MHz, pyridine-*d*_5_) δ 8.34 (d, *J* = 7.6 Hz, 1H), 8.28 (s, 1H), 7.72 (s, 1H), 7.45 (d, *J* = 7.7 Hz, 1H), 4.75 (s, 2H), 3.44 (q, *J* = 12.4 Hz, 2H), 2.60 (m, 2H), 0.98 (d, *J* = 6.4 Hz, 3H), 0.95 (s, 3H), 0.58 (s, 3H). ^13^C NMR (151 MHz, pyridine-*d*_5_) δ 176.82, 136.11, 134.98, 130.16, 127.90, 126.06, 124.1, 70.65, 59.43, 56.54, 52.05, 43.10, 40.49, 40.13, 38.45, 36.04, 35.95, 35.28, 32.14, 32.09, 31.13, 27.29, 26.83, 24.09, 18.87, 12.48. HRMS (ESI-MS) *m*/*z* for C_33_H_49_F_3_NO_3_^+^ [M + H]^+^ calc. 564.3660; found 564.3665.

##### 2.1.5.14 1-((3*S*,5*R*,10*S*,13*R*)-17-((*R*)-4-carboxybutan-2-yl)-3-hydroxy-10,13-dimethylhexadecahydro-1*H*-cyclopenta[a]phenanthren-3-yl)-*N*-(4-(trifluoromethyl)benzyl)methanaminium Chloride (**6n**)

White solid. Yield 25%. Mp. 228 °C. ^1^H NMR (600 MHz, DMSO-*d*_6_) δ 7.77–7.76 (d, *J* = 8.2 Hz, 2H), 7.73 (d, *J* = 8.2 Hz, 2H), 4.21 (s, 1H), 2.74 (s, 2H), 0.85 (s, 3H), 0.80 (d, *J* = 6.4 Hz, d, 3H), 0.55 (s, 3H). ^13^C NMR (151 MHz, DMSO-*d*_6_) δ 175.83, 136.25, 131.76, 130.18, 126.03, 126.01, 125.43, 123.62, 69.35, 57.64, 56.33, 56.09, 50.80, 42.75, 35.63, 35.26, 34.69, 31.24, 31.17. 29.63, 28.18, 26.68, 26.28, 23.77, 18.58, 12.29. HRMS (ESI-MS) *m*/*z* for C_33_H_49_F_3_NO_3_^+^ [M + H]^+^ calc. 564.3660; found 564.3652.

##### 2.1.5.15 *N*-(3,5-bis(trifluoromethyl)benzyl)-1-((3*S*,5*R*,10*S*,13*R*)-17-((*R*)-4-carboxybutan-2-yl)-3-hydroxy-10,13-dimethylhexadecahydro-1*H*-cyclopenta[a]phenanthren-3-yl)methanaminium Chloride (**6o**)

White solid. Yield 10%. Mp. 222 °C. ^1^H NMR (600 MHz, DMSO-*d*_6_) δ 11.79 (s, 1H), 9.20 (s, 1H), 8.35 (d, *J* = 24.5 Hz, 2H), 8.16 (s, 1H), 4.84 (s, 1H), 4.34 (s, 2H), 2.85 (s, 2H), 2.16 (m, 2H), 0.9 (s, 3H), 0.86 (d, *J* = 6.5 Hz, 3H), 0.61 (s, 3H). ^13^C NMR (126 MHz, DMSO-*d*_6_) δ 174.83, 131.74, 130.26, 129.99, 122.64, 122.16, 68.94, 57.77, 55.83, 55.65, 49.83, 36.91, 35.15, 34.79, 30.73, 30.68, 25.81, 23.76, 23.31, 18.14, 11.83. HRMS (ESI-MS) *m*/*z* for C_34_H_48_F_6_NO_3_^+^ [M + H]^+^ calc. 632.3533; found 632.3545.

### 2.2 In vitro Assays

#### 2.2.1 PTP1B Inhibition Assay

A spectrocolorimetric method previously described^3^ was employed to test the inhibitory effect of the new compounds against PTP1B. Briefly, the compounds and controls were carried out in a final volume of 100 μL of TRIS 50 mM, pH 6.8 containing purified *h*PTP1B 66 nM (*h*PTP1B_1–285_ and *h*PTP1B_1–400_) and pNPP 0.5 mM. Enzyme solutions containing pNPP and inhibitors were incubated at 37 °C for 15 min. The hydrolysis product’s *para*-nitrophenol (pNP) absorbance was measured at 405 nm using a 96-microplate absorbance reader (Accuris SmartReader 96). The percentage of inhibition was plotted as a function of the inhibitor concentrations. IC_50_ was calculated by regression analysis using eq 1) (OriginPro 2018 (64 bit) SR1).
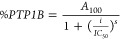
1where *%PTP1B* is the percentage of inhibition, *A*_100_ is the maximum inhibition, *i* is the inhibitor concentration, *IC*_50_ is the concentration required to inhibit the enzyme’s activity by 50%, and *s* is the cooperative degree.

#### 2.2.2 Enzyme Kinetics

Some compounds were selected for enzyme kinetics assay to determine their inhibition mechanism. The test was conducted similarly to the IC_50_, in the same experimental conditions but varying the concentrations of pNPP by 0.1 mM increments (0.1–0.5 mM) and using five increasing concentrations of each inhibitor according to previously determined IC_50_ values. The experiments were performed in duplication.^3^ The Supporting Information contains detailed eqs (E1–E5) used to determine the kinetic parameters and mechanisms underlying PTP1B inhibition.

#### 2.2.3 Selectivity Assay

Similarly to the IC_50_, the assays to determine the selectivity were carried out in the same experimental conditions using *h*PTP1B_1–400_ and *h*TCPTP.

#### 2.2.4 Expression and Purification of *h*TCPTP

The full-length of human T-cell protein tyrosine phosphatase (*h*TCPTP) coding gene sequence *PTPN2* (NCBI Reference Sequence: NP_002819.2, 415 aa) codon optimized for*Escherichia coli* was obtained from Gene Universal Inc. (Newark, DE, USA). The expression vector pET-28a(+)-TEV was obtained from Invitrogen (Waltham, MA, USA). *E. coli* strain DH5α and BL21 (DE3) were purchased from Invitrogen (Waltham, MA, USA). The gene *PTPN2* was subcloned into the expression vector pET-28a (+)-TEV, using the *NDEI* and *BAMHI* cloning sites. The construction pET-28A(+)-TEV–*PTPN2* was transformed into *E. coli* strain BL21 (DE3). Transformed *E. coli* cells were grown in LB media containing kanamycin (30 mg/mL) at 37 °C for 6 h with continuous agitation (250 rpm). Once the cultures reached an *A*_600_ of 0.6 (about 6 h), protein expression was induced with 1 mM IPTG at 37 °C, 250 rpm for 8 h. After centrifuge (4500 rpm, 15 min, 4 °C), the grown and IPTG-induced cultures and bacterial pellet were resuspended in Tris buffer (pH 6.8) and lysed by sonication (10 cycles, intervals of 30 s) in an ice–water bath with an ultrasonic processor (Cole-Parmer, Vernon Hills, IL, USA). The cell lysate was centrifuged (13000 rpm, 15 min, 4 °C), the supernatant was filtered with a PVDF membrane (pore size 0.45 μm) (Argos, Vernon Hills, IL, USA) and loaded onto a HisTrap HP immobilized metal affinity chromatography (IMAC) column (Cytiva, Marlborough, MA, USA). The column was equilibrated with three column volumes of 50 mM Tris, pH 6.8. His-tagged protein was eluted with Tris 50 mM and 300 mM imidazole with one column volume. Phosphatase activity of the collected fractions was confirmed by the pNPP activity assay (see below). Fractions containing *h*TCPTP were dialyzed in 50 mM Tris, pH 6.8. The purity of the protein was followed by SDS-polyacrylamide gel electrophoresis (SDS-PAGE) using a 12% resolving gel.

### 2.3 Structural Model of *h*PTP1B_1–400_ and *h*TCPTP_1–415_

Structural models of the proteins *h*PTP1B_1–400_ and *h*TCPTP_1–415_ were obtained from the AlphaFold Protein Structure Database developed by DeepMind and EMBL-EBI (https://alphafold.ebi.ac.uk/). The UniProt code P18031 corresponds to the *PTPN1* gene, and P17706 to *PTPN2* gene, which corresponds to the proteins *h*PTP1B_1–400_ and *h*TCPTP_1–415_, respectively. The PDB file was downloaded from the following link: https://alphafold.ebi.ac.uk/entry/P18031 for *h*PTP1B_1–400_ and https://alphafold.ebi.ac.uk/entry/P17706 for *h*TCPTP_1–415_. Subsequently, these models were subjected to a MDS for 200 ns to obtain a folding with biological relevance of the intrinsically structural zone (300 to 400 amino acids) in the case of *h*PTP1B_1–400_^33^ and to *h*TCPTP_1–415_ (314 to 415 amino acids).

### 2.4 Molecular Docking

Docking analysis was done using the structural model of the *h*PTP1B_1–400_ and *h*TCPTP_1–415_ obtained after the MDS. PTP1B_1–298_ was retrieved from Protein Data Bank (https://www.rcsb.org/.) (PDB ID: 1C83),^34^ and the water molecules were removed using PyMOL 2.4.0. Structures **6c**–**6f**, **6k**–**6o**, LA, TCS401, pNPP, and UA were constructed and minimized using AVOGRADRO software.^35^ AutoDockTools 1.5.4 was used to prepare the pdb files of the protein and compounds. Polar hydrogen atoms and the Kollman united-atom partial charges were added to the protein structures. In contrast, Gasteiger–Marsili charges and rotatable groups were automatically assigned to the structures of the ligands. We used idock to run all the docking simulations. The grid box size was 68 Å × 94 Å × 84 Å, 100 Å × 100 Å × 100 Å, and 100 Å × 100 Å × 100 Å in the *x*, *y*, and *z* dimensions and central coordinates of 25.6, 65.8, 42.1; 48.61, 14.04, 4.97; and 90.47, 52.41, 76.27 for *x*, *y*, *z*, to *h*PTP1B_1–400_, PTP1B_1–298_, and *h*TCPTP_1–415_, respectively, with exhaustiveness of 25. The best conformational states were visualized with PyMOL version 2.4.0 and Maestro Visualizer v.21.1.020298.

### 2.5 Molecular Dynamics Simulation Studies

The coordinates of the ligands resulting from the docking study were processed with antechamber to generate suitable topologies for the LEaP module from AmberTools22.^36,37^ Each structure and complex was subjected to the following protocol: hydrogen and other missing atoms were added using the LEaP module with the parm99 parameter set (ff19SB), Na^+^ counterions were added to neutralize the system, the complexes were then solvated in an octahedral box of explicit TIP3P model water molecules localizing the box limits at 12 Å from the protein surface. MDS was performed at 1 atm and 310 K, maintained with the Berendsen barostat and thermostat, using periodic boundary conditions and particle mesh Ewald sums (grid spacing of 1 Å) for treating long-range electrostatic interactions with a 10 Å cutoff for computing direct interactions. The SHAKE algorithm was used to satisfy bond constraints, allowing the employment of a 2 fs time step to integrate Newton’s equations as recommended in the Amber package.^36,37^ Amber f99SB force field^37−39^ parameters were used for all residues, and Gaff force field^40^ parameters were used for the ligands. All calculations were made using graphics processing units (GPU, NVIDIA GeForce RTX-4090) accelerated MDS engine in AMBER (pmemd.cuda), a program package that runs entirely on CUDA-enabled GPUs.^41^ The protocol consisted in performing an optimization of the initial structure, followed by 50 ps heating step at 298 K, 50 ps for equilibration at constant volume, and 500 ps for equilibration at constant pressure. Several independent 200 ns MDS were performed. Frames were saved at 100 ps intervals for subsequent analysis.

#### 2.5.1 Trajectory Analysis

The analyses used CPPTRAJ^33^ as part of AmberTools22 utilities and Origin 9.0. First, the root mean square deviation (RMSD) and root mean square fluctuation (RMSF) calculations were made, considering the C, CA, and N; only the CA was used for the distances. The graphics were built with Origin 9.0, and the trends were adjusted with smooth function processing (method Lowess). Binding free energies calculated by molecular mechanics/Poisson–Boltzmann surface area (MM/PBSA) were calculated using a technique that combines molecular mechanics’ energy with implicit solvation models to estimate binding free energies.^42,43^

## 3 Results and Discussion

### 3.1 Chemistry

The newly designed compounds (**6a**–**6o**) based on lithocholic acid skeleton were synthesized according to Scheme 1. Briefly, lithocholic acid was esterified to its methyl ester using methanol and SOCl_2_ to give compound **2** in 97% of yield. This intermediate was then oxidized with pyridinium dichromate^5^ to the corresponding 3-keto derivative (**3**) in high yield (90%). The key oxirane intermediate **4** was prepared in moderate yield (86%) by Corey–Chaykovsky epoxidation^44^ from **3** using a method previously reported for androsterone derivatives.^45,46^ Afterward, compounds **6a**–**6o** were prepared through a nucleophilic opening of oxirane **4** with *para* or *meta*-substituted benzylamines in refluxing ethanol.^47^ The resulting intermediates (**5a**–**5o**) underwent purification via a chromatography column before being hydrolyzed under alkaline conditions and then acidified to give the final compounds (**6a**–**6o**) in varying yields of 10 to 80%.

#### 3.1.1 X-ray and NMR Characterization

Due to the A/B cis ring junction, 5β steroids have a more significant steric hindrance on the alpha face of the carbonyl in the C3 position. As a result, they produce 3β-oxiranes when exposed to Corey–Chaykovsky reaction conditions with dimethylsulfoxonium methylide. A previous study^48^ has confirmed this observation using single crystal X-ray diffraction for methyl (3β-oxirane)-12α-hydroxy-5β-cholan-24-oate synthesized from its 3-keto derivative using the same reaction conditions. However, the stereochemistry of 3-oxirane **4** is presently unknown and needs to be determined to deduce the stereochemistry of the final compounds **6a**–**6o**. To determine it, we carried out a single crystal X-ray diffraction of this intermediate. As anticipated, the data from this study verified the 3β-oxirane group of steroid 4 (Figure 2).

Upon analyzing the ^1^H NMR spectra of the final compounds (**6a**–**6o**), we observed that the methylene signal from the oxirane group (located at 5.0 ppm) of compound **4** disappeared. Instead, two new signals emerged around δ_H_ = 2.7 and 4.1 ppm, corresponding to the 25-CH_2_– and 26-CH_2_– methylenes groups, respectively. Furthermore, the signals related to the benzylic ring appeared between δ_H_ = 7 and 8 ppm. The broad single signal around δ_H_ = 4.8 ppm was assigned to 3-OH, while the broad single signal at δ_H_ = 9 ppm (integrating for two hydrogens and exchanging with D_2_O) was assigned to the Bz–NH_2_^+^–CH_2_– group. It is worth noting that this signal integrates for only one proton when deuterated pyridine is used, suggesting that the final compounds were obtained as a hydrochloride salt. Conversely, in ^1^H NMR, chemical shifts from δ_H_ = 8 to 7 ppm with coupling constants around 8 Hz were assigned to the benzylic moiety of these compounds, while a broad singlet around δ_H_ = 11.8 ppm was assigned to 24-COOH. For ^13^C NMR, chemical shifts from δ_C_ = 135 to 120 ppm were assigned to the benzene ring. Signals near to δ_C_ = 174 and 70 ppm belong to 24-COOH and quaternary C3 carbon, respectively.

Since intermediate **4** in this study is a 3β-oxirane, the final compounds **6a**–**6o** are expected to have a tertiary 3β–OH group. Nevertheless, to determine the spatial orientation of the benzylaminomethyl substituent at the C3 position, 2D experiments, including HSQC, HMBC, COSY, and NOESY, were performed (Figures S30–33). The chemical shift data of lithocholic acid, previously reported by Waterhouse et al.,^49^ were also used for the analysis. In addition, these experiments allowed us to determine the complete assignment of carbon and proton signals for compound **6f** (Table S2). The position 19-CH_3_ was determined, with a chemical shift of δ_H_ = 0.90 ppm and δ_C_ = 23.28 ppm. Using HMBC and HSQC experiments, the positions of carbons C10, C5, and C1 were determined, with chemical shifts of δ_C_ = 34.19, 38.77, and 30.55 ppm, respectively. The protons 5β-CH and 1-CH_2_ were found in chemical shifts of δ_H_ = 1.29, 1.69, and 1.16 ppm, respectively. The position of C25 was determined using the same experiments. This group was crucial in determining the orientation of the group attached to C3 of the steroid. The CH_2_ group at position 25 showed a simple signal that integrated for 2 protons and had a chemical shift of δ_H_ = 2.71 ppm and δ_C_ = 56.66 ppm. However, it is worth noting that the multiplicity of this signal changes depending on the solvent. In DMSO-*d*_6_, it appears as a single signal, but in pyridine-*d*_5_, it splits into a quadruple at a chemical shift of δ_H_= 3.37 ppm. The HMBC experiment showed that the same methylene group interacted with the carbons of positions C2, C3, and C4, which had chemical shifts of δ_C_= 35.13, 68.79, and 29.29 ppm, respectively. Using the HSQC and COSY experiments, the protons corresponding to methylenes 2-CH_2_ and 4-CH_2_ were assigned to δ_H_ = 1.68 and 1.17 ppm (for 2-CH_2_) as well as δ_H_ = 1.33 and 1.25 ppm (for 4-CH_2_). With the NOESY experiment, the orientation of the positions’ protons was determined. Specifically, an interaction through the space of the proton 5β-H with the signal at 1.25 ppm of the 4-CH_2_ was observed using the NOESY experiment. Therefore, this signal was assigned as 4β-H while the other one at 1.33 ppm was assigned as 4α-H. Furthermore, the 19-CH_3_ signal, oriented toward the beta side of the steroid, was observed to have a NOE interaction with the 1.16 ppm signal of the 1-CH_2_ group. Thus, this signal was assigned as 1β-H, while the other signal at 1.69 ppm was assigned as 1α-H. The COSY and NOESY experiments and the data from positions 1-CH_2_ and 4-CH_2_ determined that the chemical shift at 1.68 and 1.17 ppm correspond to 2α-H and 2β-H, respectively. Finally, it was observed that only signals with a chemical shift of 1.68 and 1.33 ppm, corresponding to the 2α-H and 4α-H positions, showed an NOE correlation with the 25-CH_2_ group. Therefore, it is assumed that the benzylaminomethyl group attached to the C3 position of lithocholic acid is oriented toward the α-face of the steroidal skeleton, while 3-OH is oriented toward the β-face.

### 3.2 Biological Assays

#### 3.2.1 PTP1B Inhibitory Effect of 3-Benzylaminomethyl Lithocholic Acid Derivatives

Compounds **6a**–**6o** were assessed as potential inhibitors of the PTP1B enzyme. The experiment used two variants of the enzyme, *h*PTP1B_1–285_ and *h*PTP1B_1–400_ (short and long form, respectively). Different isoforms of the enzyme were used to infer the possible binding site of the compounds. The short form only contains the catalytic domain, while the long form consists of both the catalytic domain and the unstructured zone. The enzyme was obtained, and the assay was conducted using a previously reported method.^3^ Coronell-Tovar et al. reported an analysis highlighting the importance of *h*PTP1B_1–400_ in PTP1B inhibition because this form is mainly expressed *in vivo*.^3^ Thus, we first tested different concentrations of reference inhibitors LA, UA, SUR, TCS401, and CGA. These inhibitors provide IC_50_ values of 14.0, 6.8, 2.6, 8.1, and 392.3 μM, respectively (Table 1). The first interesting finding of this study is that incorporating various benzylaminomethyl moieties in the C3 position of lithocholic acid yielded compounds that were more potent than LA itself. For example, compound **6n** (IC_50_ = 5.3 μM) is 3-fold more potent than LA. Nonetheless, compounds **6g**–**6j** showed no activity against the *h*PTP1B_1–400_ enzyme at the concentrations tested (100 μM). The second interesting finding is that LA derivatives having a trifluoromethyl group at the aromatic ring (**6m** and **6n**) were the most potent inhibitors in this series, with IC_50_ values of 7.3 and 5.3 μM, respectively. Furthermore, these inhibitors are more potent than TCS401 and CGA, but have a similar potency to UA.

**Table 1:** PTP1B Inhibitory Effect of New Lithocholic Derivatives^a^

		IC_50_± SD (μM)	
compound	R	*h*PTP1B_1–285_	*h*PTP1B_1–400_
**6a**	H	ND	33.1 ± 2.7
**6b**	4’-OH	ND	26.9 ± 3.5
**6c**	3′-OCH_3_	ND	10.5 ± 0.2
**6d**	4′-OCH_3_	ND	26.6 ± 0.9
**6e**	3′-CH_3_	53.8 ± 2.7	16.3 ± 0.5
**6f**	4′-CH_3_	46.0 ± 1.7	34.3 ± 0.5
**6g**	3′-F	ND	>100
**6h**	4′-F	ND	>100
**6i**	3′-Cl	ND	>100
**6j**	4′-Cl	ND	>100
**6k**	3′-COOH	ND	32.3 ± 1.2
**6l**	4′-COOH	ND	12.6 ± 0.7
**6m**	3′-CF_3_	34.9 ± 0.4	7.3 ± 0.5
**6n**	4′-CF_3_	16.3 ± 0.9	5.3 ± 0.2
**6o**	3′,5′-bis-CF_3_	43.0 ± 1.3	11.0 ± 0.2
**LA**	–	22.2 ± 0.9	14 ± 0.9
UA^b^	–	26.59 ± 1.46	6.83 ± 0.07
SUR^b^	–	9.95 ± 0.39	2.59 ± 0.15
**TCS401**	–	12.4 ± 1.0	8.1 ± 0.9
**CGA**	–	718.6 ± 93.4	392.3 ± 16.8

a ND = Not determined

b IC_50_values retrieved from reference.^3^

We have found that adding fluorinated groups to the aromatic rings of semisynthetic PTP1B inhibitors plays a crucial role in their ability to inhibit PTP1B. For example, Mao et al.^5^ discovered that fused *N*-phenylpyrazoles on ring A of lithocholic acid could act as PTP1B inhibitors. Among the compounds they tested, the *para*-fluoro substituted one was the most potent (IC_50_ = 0.42 μM) and was found to be a competitive inhibitor. The trifluoromethyl group is an electron-withdrawing substituent and can form nonclassical hydrogen bonds. According to Table S1, considering electronic (Hammet constant, σ), lipophilic (Hansch constant, π), and steric (Taft constant, E_S_) parameters, compounds **6m**–**6o** have the highest values of σ and π constants and more negative values of E_S_ constant. This suggests that these compounds can bind to lipophilic and electropositive sites within the PTP1B enzyme. On the other hand, the presence of polar groups (COOH and OH for compounds **6b**, **6k**, and **6l**) reduces their potency since these decrease the lipophilic character of the molecule. The steric effect also plays an essential role in the inhibitory effect of the PTP1B enzyme. For instance, compound **6o**, which contained two trifluoromethyl groups in the aromatic ring, decreased potency, indicating that the disubstitution of the benzylic ring with bulky groups is not well-tolerated to inhibit the enzyme.

LA derivatives, **6e**, **6f**, **6m**, **6n**, and reference inhibitors LA, TCS401, CGA, SUR, and UA were assessed to determine their affinity for short (*h*PTP1B_1–285_) versus long (*h*PTP1B_1–400_) PTP1B forms. Moreover, this experiment will help us to understand if residues 286–400 are important for interacting with the proposed inhibitors and newly synthesized compounds. IC_50_ values (Table 1) revealed that TCS401 and CGA had a similar or 2-fold better potency for *h*PTP1B_1–400_ over *h*PTP1B_1–285_, indicating that the presence or absence of residues 286–400 of PTP1B does not have a significant impact on the inhibitory activity of both inhibitors. This can be explained by the fact that both interact within the catalytic domain of PTP1B, thus showing a competitive inhibitory activity. Interestingly, SUR and UA have a 4-fold higher potency for *h*PTP1B_1–400_ than *h*PTP1B_1–285_. This suggests that residues 286–400 of PTP1B may be involved in the interaction with both inhibitors, increasing their affinity. When LA was tested, we found that this steroid showed 1.5-fold more potency against *h*PTP1B_1–400_ than *h*PTP1B_1–285_. However, LA derivatives **6m** and **6n** showed around 5- and 3-fold more potent inhibitory effects on *h*PTP1B_1–400_, respectively, whereas **6e** displayed less inhibitory potency for *h*PTP1B_1–400_. The study results indicate that if benzylaminomethyl group is added to LA at C3 position, it will enhance the affinity for the long PTP1B form. Additionally, this structural modification to LA may help to interact with residues 286 to 400 of PTP1B, which is crucial for their inhibitory effect. This interaction is particularly significant when these compounds also contain a trifluoromethyl group at the benzyl moiety, as it helps to position them in a conformation within the long PTP1B form that further increases the inhibitory effect.

#### 3.2.2 Enzymatic Kinetic Studies

**Figure 3:**
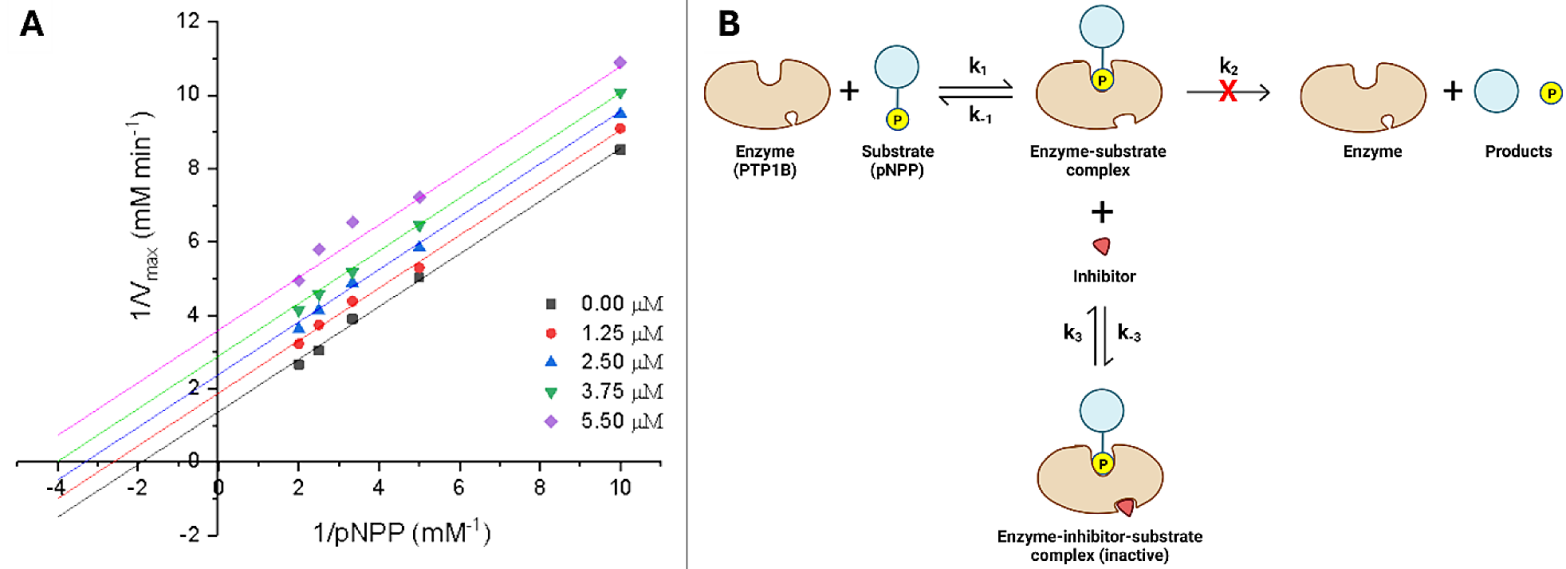
Enzyme kinetics of *h*PTP1B_1–400_ inhibition. Lineweaver–Burk plot shows the mechanism of inhibition of **6m** (A). The plot represents the reciprocal of the reaction velocity (1/V) as a function of the reciprocal pNPP concentration (1/pNPP). Data are representative of two independent experiments. The inhibitory mechanism for each compound was determined by fitting data to the equations defined for competitive, noncompetitive, uncompetitive, and mixed inhibition models. Data displayed in the graphs correspond to the best fit for each inhibition model (based on the *R*^*2*^ coefficient) (OriginPro 2018 (64 bit) SR1)). (B) Uncompetitive inhibition model. E: enzyme; S: substrate; I: inhibitor; P: product. Adapted from “Uncompetitive inhibition”, by BioRender.com (2024). Retrieved from https://app.biorender.com/biorender-templates.

To determine the inhibition type of the newly synthesized compounds, we selected derivatives **6c**, **6d**, and **6k**–**6o** and LA for enzymatic kinetic studies. We performed this assay as described previously.^3^Table 2 and Figure 3A show the results of kinetic studies for new lithocholic derivatives. *K*_i_ values reported for positive controls, SUR, UA, and CGA are 3.0, 4.0, and 1030 μΜ, respectively. SUR and CGA exhibit competitive inhibition,^3,27,28^ while UA displays mixed inhibition.^3^ Conversely, TCS401 has been reported as a competitive inhibitor with a *K*_i_ value of 4.7 μM.^11,50^ In our study, LA exhibited a *K*_i_ value of 5.5 μM and acted as an uncompetitive inhibitor of PTP1B. However, when a benzylaminomethyl moiety was added to C3 position of LA, the potency against PTP1B was enhanced. This was shown by the *K*_i_ values of **6m**, **6n**, and **6o**, which were 2.5, 3.4, and 3.6 μM, respectively. These compounds displayed a better affinity than UA (4.03 μM). Among this series, **6m** had the lowest *K*_i_ value and thus better affinity, even better than that of SUR (3.0 μM), the most potent positive control in this study. Additionally, the compounds **6c**–**6o** displayed uncompetitive inhibition (Figure 3A) similar to LA.

**Table 2:** Kinetic Parameters and Inhibition Type of New Lithocholic Derivatives against *h*PTP1B_1-400_

compound	*V*_max_ (mM/min)	*K*_m_ (mM)	*K*_i_ (μM)	inhibition mechanism
**6c**	0.64 ± 0.02	0.38 ± 0.02	4.0 ± 0.2	Uncompetitive
**6d**	1.10 ± 0.09	0.78 ± 0.09	19.7 ± 2.6	Uncompetitive
**6k**	0.80 ± 0.06	0.48 ± 0.06	11.0 ± 1.0	Uncompetitive
**6l**	1.41 ± 0.17	0.95 ± 0.15	10.7 ± 1.6	Uncompetitive
**6m**	1.05 ± 0.18	0.78 ± 0.2	2.5 ± 0.4	Uncompetitive
**6n**	0.73 ± 0.06	0.52 ± 0.07	3.4 ± 0.3	Uncompetitive
**6o**	2.7 ± 0.4	1.9 ± 0.3	3.9 ± 0.7	Uncompetitive
**LA**	1.7 ± 0.3	1.2 ± 0.3	5.5 ± 1.1	Uncompetitive
**UA**^a^	0.63 ± 0.08	1.70 ± 0.33	4.03 ± 0.8	Mixed
**SUR**^a^	0.64 ± 0.02	0.29 ±0.02	3.0 ± 0.2	Competitive
**TCS401**	ND	ND	4.7^b^	Competitive^c^
**CGA**^a^	6.71 ± 3.13	3.63 ± 1.88	1030 ± 70	Competitive

a *V*_max_, *K*_m_, and *K*_i_ and inhibition mechanism data retrieved from reference 3.^3^

b *K*_i_ value retrieved from reference 50.^50^

c Inhibition mechanism using *h*PTP1B_1–285_ according to reference 11.^11^

This is a noteworthy result, as most reported PTP1B inhibitors are competitive by binding to the catalytic site. However, due to the high conservation of this domain among the PTP family, the selectivity of these inhibitors is often low. Based on their uncompetitive inhibitory activity and preferred inhibition of *h*PTP1B_1–400_ by LA inhibitors, it can be inferred that **6a**–**6o** have a high potential for selective inhibition of PTP1B. This is because they do not bind to the catalytic site, but only to PTP1B when its substrate binds (Figure 3B).

#### 3.2.3 Selectivity Assays

To develop effective PTP1B inhibitors, it is essential to ensure their selectivity, as protein tyrosine phosphatases share a highly conserved catalytic domain and a significant sequence homology. For instance, PTP1B and TCPTP have a 74% amino acid sequence identity in the catalytic.^5,51,52^ Therefore, developing new PTP1B inhibitors is challenging due to the potential adverse effects resulting from the lack of TCPTP activity. Thus, it is desirable to inhibit PTP1B rather than TCPTP. To determine the selectivity for PTP1B versus TCPTP, we tested reference inhibitors LA, UA, CGA, sodium orthovanadate (SO), TCS401, and compounds **6m**–**6o** (Table 3). As expected, SO lacks selectivity as it inhibits both PTP1B and TCPTP enzymes (IC_50_ = 0.12 and 0.18 μM, respectively), consistent with previously reported data.^53^ In the same experiment, it was observed that TCS401 showed similar results with IC_50_ values of 9.1 and 6.57 μM, respectively, whereas LA and UA only inhibited PTP1B but not TCPTP (Figure S2). Interestingly, the compounds **6m**–**6o** were found to be inactive against TCPTP in the range of concentrations tested (1 to 400 μM), making them up to 75 times more selective toward PTP1B over TCPTP (Figure S1). These findings suggest that the excellent selectivity of compounds **6m**–**6o** is mainly due to their binding to the disordered C-terminus of PTP1B. This observation is based on the preferential inhibition of the long PTP1B form by these compounds and the findings reported for trodusquemine. This aminosterol has been extensively studied as an inhibitor of PTP1B.^54^ Trodusquemine is a noncompetitive inhibitor with an IC_50_ of 1.0 μM against PTP1B and an IC_50_ of 224 μM against TCPTP.^55^ This selectivity is attributed to trodusquemine’s binding to the disordered C-terminus of PTP1B, a segment not structurally related to TCPTP or any other member of the PTP family.^56^

**Table 3:** Selectivity of the Inhibition for PTP1B over TCPTP

compound	PTP1B IC_50_ ± SD (μM)	TCPTP IC_50_± SD (μM)	selectivity
**6m**	7.3 ± 0.5	>400	Yes
**6n**	5.3 ± 0.2	>400	Yes
**6o**	11.0 ± 0.2	>400	Yes
**LA**	14.0 ± 0.9	>400	Yes
**UA**	6.83 ± 0.07	>400	Yes
**SO**	0.12 ± 0.3	0.18 ± 0.1	No
**TCS401**	8.15 ± 0.91	6.7 ± 0.6	No

#### 3.2.4 Molecular Docking Studies

**Figure 4:**
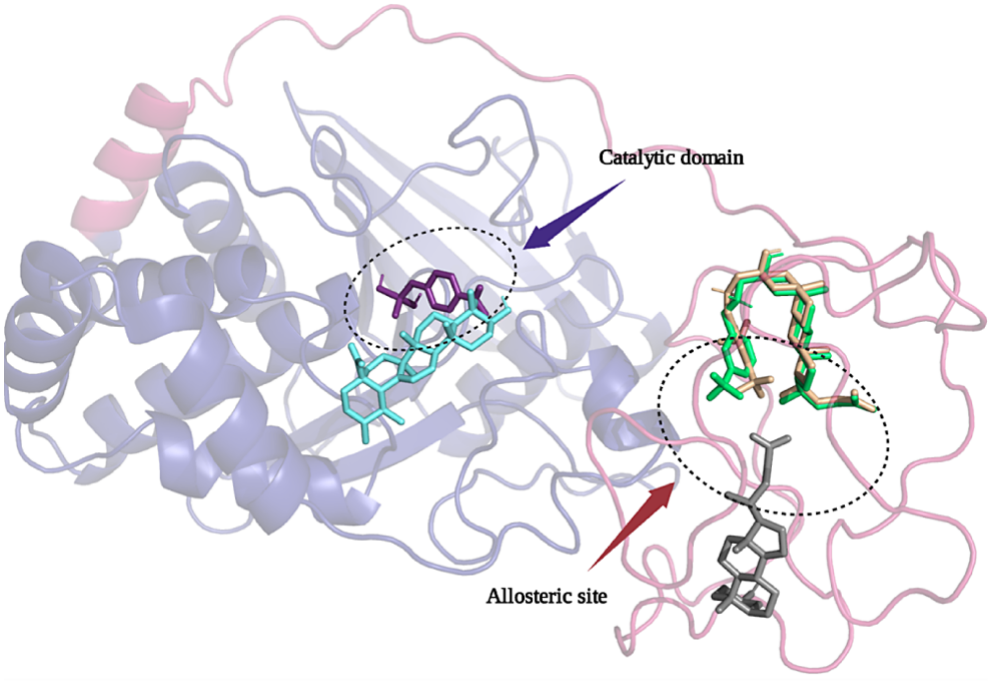
3D-structural model of *h*PTP1B_1–400_ indicating catalytic domain (blue cartoon) and a possible allosteric site of inhibition in the C-disordered terminus region (red cartoon). The predicted binding mode of UA in cyan sticks, LA in gray sticks, **6m** in green sticks, **6o** in gold sticks, and substrate pNPP in purple sticks.

**Figure 5:**
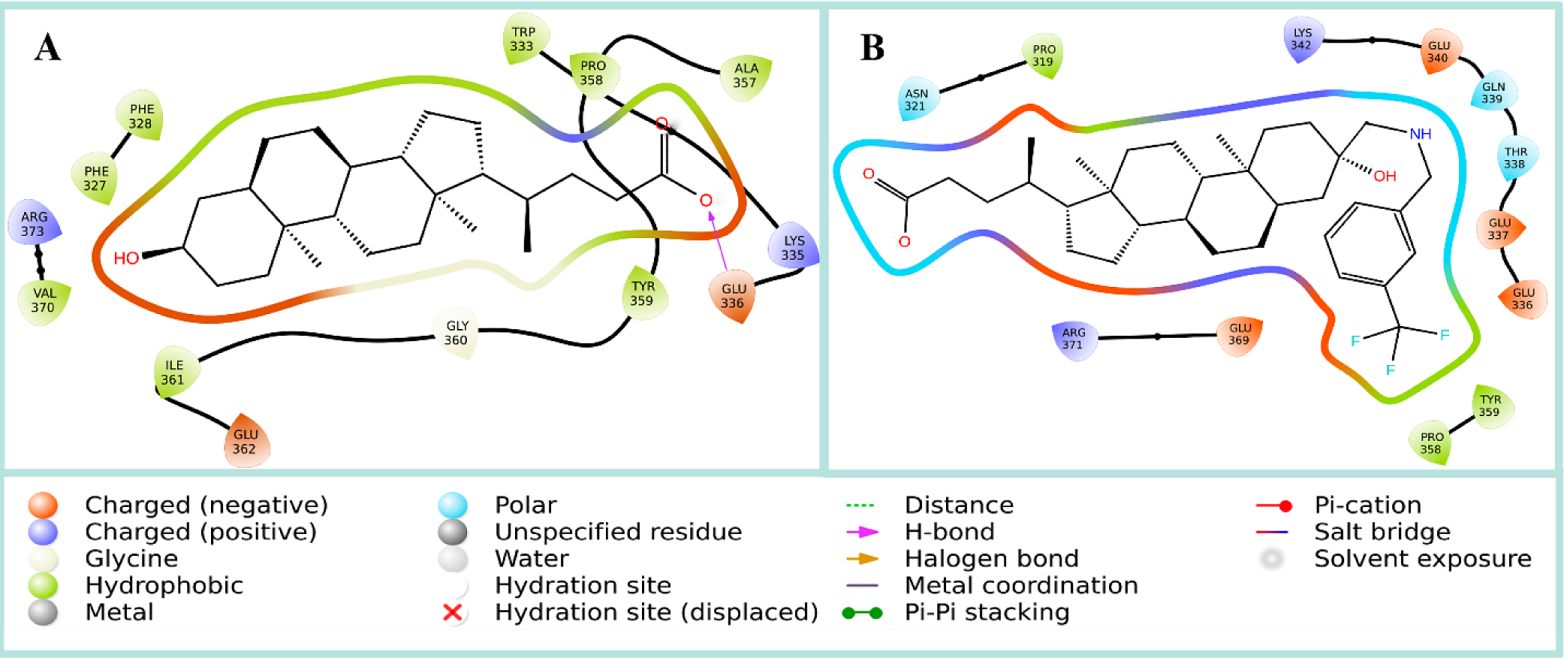
2D interacting model of LA (A) and **6m** (B) with amino acid residues in the unstructured region of *h*PTP1B_1–400_. The analysis of the interactions with residues at 4 Å is shown on the periphery.

We have designed an uncompetitive docking model based on the results of the enzymatic kinetic assay. The docking simulations were carried out using iDock^57^ and the previously reported three-dimensional structure of *h*PTP1B_1–400_.^3^ First, we docked the *para*-nitrophenylphosphate (pNPP) substrate with *h*PTP1B_1–400_. Then, we docked the ligands **6e**, **6f**, **6m**, **6n**, and **6o**, as well as LA to the unstructured region where possible inhibitor sites have also been reported. The controls UA and TCS401 were docked without pNPP. Both compounds bind to the secondary noncatalytic pTyr-binding site (Tyr^20^, Arg^24^, Arg^254^, and Gly^259^) via hydrophobic and polar interactions (Figure S4). In addition, UA presents interactions with Tyr^46^ and Asp^48^, which are involved in substrate recognition^54^ while a polar interaction with Gln^262^ was also observed. On the other hand, LA and their derivatives displayed favorable binding energies (Table 4) even better than TCS401 (−5.89 kcal/mol). The interplay between LA and the intrinsically unstructured C-terminal domain was primarily characterized by the hydrophobic interactions with Phe^327,328^, Ile^361^, and Val^370^. Furthermore, a polar interaction occurred between the 3-OH group of LA and Arg^373^ (Figures 4, 5A). These interactions are noteworthy due to the reported presence of two alpha helices, residues 320–327 (α8’) and 360–377 (α9’) in the C-terminal domain. These helices have been reported to play a crucial role in PTP1B inhibition.^56,58^ For **6e**, the steroid backbone formed hydrophobic interactions with Trp^333,^ Val^334^, and Ile^346^. Interactions between the NH group and residues of α8’ helix (Lys^323^, Arg^325^), and α9’ helix (Arg^371^), an electropositive site of the unstructured region, were also observed, as well as an H-bond interaction between 3-OH group and Asn^321^ (Figure S3). Ligand **6f** resulted in three hydrophobic interactions near the catalytic domain with Val^49^, Phe^182^, and Ile^219^. Polar interactions were also formed between Ser^28^, Gln^262^, and C24 carboxylate (Figure S3). **6m** (**6e** isostere) showed hydrophobic interactions between its trifluoromethyl group and the side chain of residues Pro^358^ and Tyr^359.^ In addition, the ammonium group showed H-bond interactions with Gln^339^ and Thr^338^, while the C24 carboxylate group of this compound served as an HBA with Asn^321^. Furthermore, **6m** formed interactions with electropositive and electronegative regions formed by Lys^342^, Arg^371^, Glu^336, 337, 340, 369^, and Thr^338^ (Figures 4 and 5B). For **6n** the benzylaminomethyl group is bound to the second pTyr recognition site, exhibiting hydrophobic interactions (Tyr^20^, Ala^27^, and Met^258^) and nonclassical H-bond interactions between the trifluoromethyl group and Arg^24, 254^. Furthermore, it exhibited hydrophobic interactions with catalytic site residues (Val^49^, Phe^182^, and Ile^219^) and a polar interaction with Gln^262^ (Q-loop) (Figure S3). On the other hand, the C24 carboxylate group of **6o** is an HBA of Asn^321^. Moreover, one trifluoromethyl group shows a hydrophobic interaction with Tyr^359^, while the second shows a nonclassical H-bond with Gln^339^ and Thr^338^. Interactions with the side chains of residues Glu^369^ and Glu^340^ are formed by the ammonium group and the 3-OH group, respectively (Figure S3). Notably, LA and its derivatives **6m** and **6o** exhibit similar binding properties in the C-disordered terminus domain of the PTP1B enzyme (Figure 4).

**Table 4:** Molecular Docking and Molecular Dynamics Simulations Results for New LA Derivatives

complex	molecular docking idock score (kcal/mol)	MDS^a^Δ*G* (kcal/mol)	docking interactions residues	MDS interactions residues
PTP1B-pNPP-**6e**	–8.50	–52.71± 3.4	His^320^, Asn^321^, Lys^323^, Arg^325^, Gln^332^, Trp^333^, Val^334^, Glu^337^, Ile^346^, Arg^371^	Asn^44^, Arg^45^, Tyr^46^, Leu^88^, Pro^89^, Ser^118^, Leu^119^, Lys^120^, Gln^339^, Glu^340^, Asp^341^, Lys^342^, Asp^343^
PTP1B-pNPP-**6f**	–6.74	–57.32 ± 2.2	Arg^24^, Ser^28^, Asp^48^, Val^49^, Phe^182^, Ile^219^, Gly^259^, Gln^262^	Tyr^20^, Arg^24^, Asp^48^, Val^49^, Ile^219^, Arg^254^, Met^258^, Gly^259^, Leu^260^, Ile^261^, Gln^262^, Ile^361^, Glu^362^, Ser^363^
PTP1B-pNPP-**6m**	–6.63	–57.73 ± 8.7	Pro^319^, Asn^321^, Glu^336^, Glu^337^, Thr^338^, Gln^339^, Glu^340^, Lys^342^, Pro^358^, Tyr^359^, Glu^369,^ Arg^371^	Asn^42^, Leu^88^, Pro^89^, Asn^90^, Arg^311^, Pro^312^, Pro^313^, Lys^314^, Arg^315^, Gln^339^, Glu^340^, Asp^341^, Lys^342^, Glu^399^, Asp^400^
PTP1B-pNPP-**6n**	–7.20	–27.34 ± 5.9	Tyr^20^, Arg^24^, Ala^27^, Ser^28^, Asp^29^, Asp^48^, Val^49^, Ser^50^, Phe^182^, Ile^219^, Arg^254^, Met^258^, Gly^259^, Gln^262^	Asp^29^, Phe^30^, Pro^31^, Cys^32^, Arg^33^, Lys^36^, Asp^48^, Ser^50^, Phe^52^, Met^258^,
PTP1B-pNPP-**6o**	–7.00	–54.93 ± 4.5	Pro^319^, Asn^321^, Glu^336^, Glu^337^, Thr^338^, Gln^339^, Glu^340^, Lys^342^, Tyr^359^, Glu^369^, Arg^371^	Arg^24^, Asp^48^, Val^49^, Ile^219^, Arg^254^, Met^258^, Gly^259^, Gln^262^, Asp^343^, Cys^344^, Pro^345^, Lys^350^, Gly^351^
PTP1B-pNPP-LA	–7.31	–29.56 ± 8.9	Phe^327^, Phe^328^, Trp^333^, Lys^335^, Glu^336^, Ala^357^, Pro^358^, Tyr^359^, Gly^360^, Ile^361^, Glu^362^, Val^370^, Arg^373^	Arg^33^, Lys^36^, Met^364^, Gln^383^
PTP1B-UA	–7.45	–28.44 ± 2.6	Tyr^20^, Arg^24^, Tyr^46^, Asp^48^, Val^49^, Ile^219^, Arg^254^, Gly^259^, Gln^262^	Arg^24^, Tyr^46^, Asp^48^, Phe^182^, Ala^217^, Ile^219^, Arg^254^, Met^258^, Gly^259^, Gln^262^
PTP1B-TCS401	–5.89	–22.57 ± 1.7	Tyr^20^, Arg^24^, Ala^27^, Val^49^, Ile^219^, Arg^254^, Met^258^, Gly^259^, Ile^261^	Tyr^20^, Arg^24^, Asp^48^, Val^49^, Ile^219^, Arg^254^, Met^258^, Gly^259^, Leu^260^ Ile^261^, Gln^262^, Thr^263^

a 200 ns of Molecular dynamics simulations (MDS).

Furthermore, the presence of the benzylaminomethyl and trifluoromethyl groups increases the number of polar interactions with residues within this region of PTP1B, suggesting that the charged area is of significant importance in the enzyme’s inhibition. These findings support the uncompetitive inhibition model observed in the enzyme kinetic studies.

**Figure 6:**
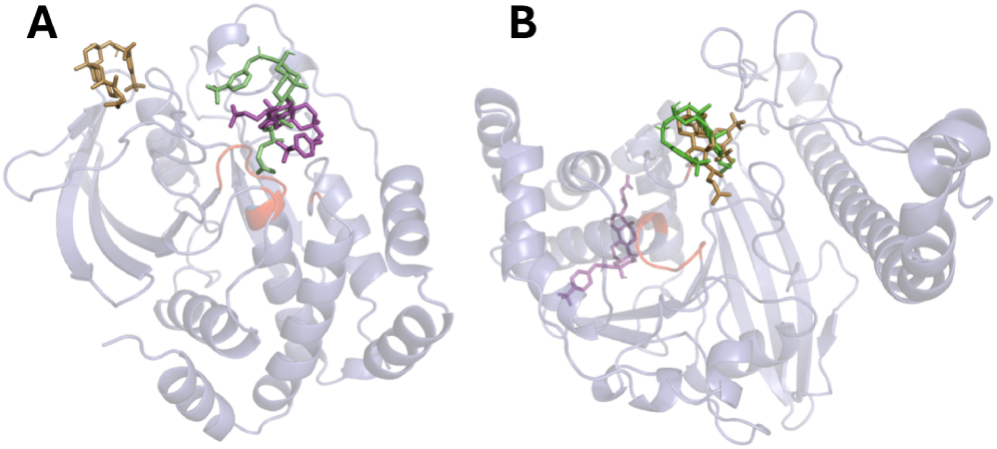
3D-structural model of PTP1B_1–298_ (PDB ID: 1C83) (**A**) and TCPTP_1–415_ (**B**) highlighting the catalytic site (red cartoon). The predicted binding mode of **6m** in green sticks, **6n** in magenta sticks, and **6o** in gold sticks.

Conversely, to understand the selectivity of LA derivatives **6m**–**6o**, between *h*PTP1B_1–285_ and *h*TCPTP_1–415_, we conducted blind docking simulations using the crystal structure of PTP1B_1–298_ (PDB ID: 1C83) and modeled structure of TCPTP_1–415_ (Figure 6). Interestingly, the compounds are bound with favorable energies by both enzymes (Table 5). In the case of the interactions carried out by these compounds in PTP1B, the carboxylate group of **6m** forms a salt bridge interaction with Cys^215^, which is the residue responsible for the catalytic activity of PTP1B. Moreover, the carboxylate group also exhibits a π interaction with the side chain of the Phe^182^ residue. The aromatic ring of the benzylamine group forms π interactions with Tyr^46^ while the CF_3_ group forms H-bonds with the Tyr^46^, Arg^45^, and Leu^119^ residues. This compound also forms hydrophobic interactions between the steroidal skeleton and Val^49^, Ala^217^, and Arg^47^ residues. On the other hand, compound **6n** has a different orientation, in which the benzylamine group carries out π interactions with Phe^182^, and the ammonium group forms an H-bond with the Gln^262^ residue, which is also involved in the catalytic activity of PTP1B. This compound also forms salt bridge interactions with Lys^120^ and an H-bond with Ser^118^. However, compound **6o** interacts in a region near the catalytic site, forming a salt bridge between the ammonium group and Asp^137^. In addition, this compound forms several H-bonds with Cys^92^, Gly^93^, Asp^137^, and Glu^136^.

**Table 5:** Molecular Docking LA Derivatives with *h*PTP1B_1-298_ and *h*TCPTP_1-415_

	molecular docking idock score (kcal/mol)	
compound	PTP1B_1–298_	TCPTP_1–415_
**6m**	–7.40	–7.92
**6n**	–6.85	–8.27
**6o**	–6.13	–8.44

When looking at the interactions of compounds **6m**–**6o** in TCPTP, it is evident that they do not interact within the catalytic site of this enzyme. For example, the carboxylate group in **6m** forms hydrogen bonds with Arg^114^, Thr^179^, and Trp^180^, while the benzylamine group interacts with Lys^118^ and Pro^181^ through hydrophobic interactions. Compound **6o** binds to a region similar to **6m**, where the ammonium group forms two salt bridge interactions with Glu^187^ and Asp^328^, and the CF_3_ groups form hydrogen bonds with Arg^114^, Asp^302^, and Arg^3^^29^. The steroidal skeleton of this compound performs hydrophobic interactions with Lys^118^ and Pro^181^ residues. Compound **6n** binds in a region far from the TCPTP catalytic site, forming salt bridge interactions between the ammonium group and Glu^237^, as well as hydrogen bonds of the carboxylate group with Gln^286^ and 3-OH with Asn^206^. Therefore, the better inhibitory activity of compounds **6m** and **6n** for PTP1B over TCPTP is likely due to their ability to interact within the catalytic site of PTP1B. As for compound **6o**, its inhibitory activity is likely due to its binding to an allosteric site of PTP1B.

#### 3.2.5 Molecular Dynamics Simulation Studies

Molecular dynamics simulations (MDS) were performed using AMBER software at 1 atm, 310 K, and 200 ns.^41^ Simulations were conducted for the complexes PTP1B-pNPP-**6e**, PTP1B-pNPP-**6f**, PTP1B-pNPP-**6m**, PTP1B-pNPP-**6n**, PTP1B-pNPP-**6o**, and controls PTP1B-pNPP-LA, PTP1B-UA and PTP1B-TCS4014, which resulted from the docking studies at the unstructured region of PTP1B. The RMSD and RMSF were analyzed during the entire MDS.

**Figure 7:**
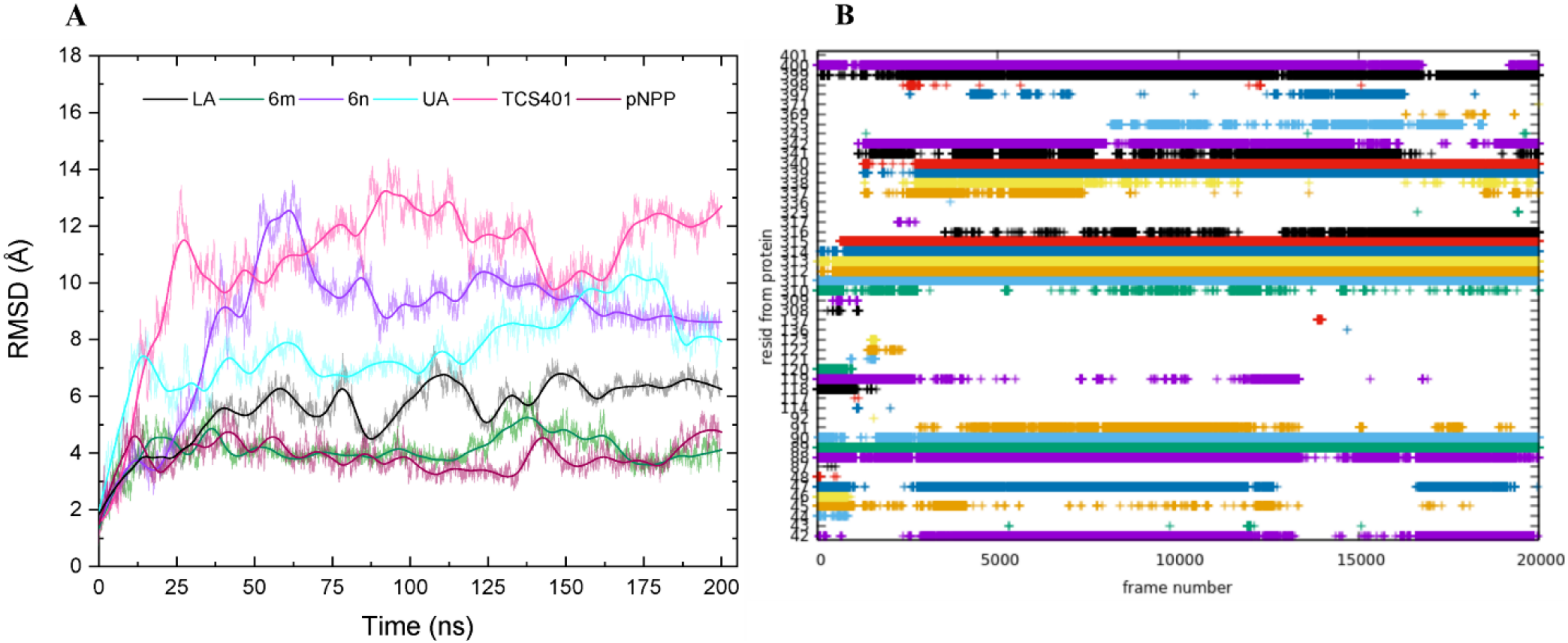
(**A**) Standard RMSD of new LA derivatives and PTP1B+pNPP systems; (**B**) Contact matrix generated from MD simulations for derivative **6m**.

Results of the RMSD analysis for overall the fluctuation of the PTP1B’s Cα carbons showed that the PTP1B-pNPP system tends to stabilize after 65 ns with an average RMSD value of 3.8 Å (Figure 7A). The TCS401 and UA controls were unable to achieve equilibrium in the system during the 200 ns MDS period, resulting in an average RMSD value of 10.7 and 6.8 Å, respectively (Figure 7A). In contrast, LA demonstrated a similar inability to stabilize the system, resulting in an average RMSD value of 7.5 Å (Figure 7A). Ligands **6e** and **6f** displayed an average RMSD similar to LA (7.8 and 7.5 Å, respectively) (Figure S5). Derivative **6o**, with an RMSD value of 8.6 Å, stabilized the system after 150 ns (Figure S5). Derivatives **6m** and **6o**, showed lower average RMSD values than the LA control. Notably, **6m** stabilized the system after 65 ns with an average RMSD value of 4.1 Å, indicating that it stabilizes the protein similarly to the pNPP substrate (Figure 7A). The evidence indicates that the binding of **6m** at the allosteric site on the unstructured region significantly stabilizes the overall conformation of PTP1B. Additionally, the binding energy for the PTP1B-pNPP-6m-system was the most favorable of all ligands (−57.73 kcal/mol). In addition, the RMSF value was used to analyze the fluctuations of amino acid residues caused by ligand binding. The chart shows that the PTP1B-pNPP-6m system had less fluctuation in the amino acid residue sequence of the unstructured region of PTP1B compared to its isostere **6e** and controls LA, UA, and TCS401 (RMSF: 3.2 Å, 2.3 Å, and 3.8 Å, respectively) (Figure S7). The average RMSF value of the PTP1B-pNPP-6m complex is equivalent to that of PTP1B-pNPP (1.8 Å).

The MDS trajectories were analyzed with CPPTRAJ and Origin 9.0. Figure 7B shows the amino acid residue contacts matrix for derivative **6m**. In the unstructured region of PTP1B, ligand **6m** forms numerous new interactions with other amino acid residues in the unstructured region of PTP1B (Table 4), which persist consistently throughout the entire MDS trajectory. In contrast, the other ligands and controls UA and TCS401 form fewer interactions (Table 4, Figures S7–S13). These findings provide insight into the uncompetitive nature of the inhibition and the lowest *K*_i_ value of compound **6m** observed in the enzyme kinetic studies. In addition, it also explains why this compound has a higher affinity for the long form of the PTP1B protein (*h*PTP1B_1–400_ IC_50_ = 7.3 μM) compared to the short form (*h*PTP1B_1–285_ IC_50_ = 34.9 μM).

## 4 Conclusions

Fifteen new 3α-benzylaminomethyl lithocholic acid derivatives (**6a**–**6o**) were synthesized in five steps from LA. The global yields of these derivatives were up to 61%. These compounds were tested against PTP1B and exhibited up to 4-fold higher affinity for *h*PTP1B_1–400_ (long form) than *h*PTP1B_1–285_ (short form). Among this series, compounds **6m** and **6n** were the most potent inhibitors, with IC_50_ values of 7.3 and 5.3 μM against *h*PTP1B_1–400_. We found that a trifluoromethyl group at the 3α-benzylaminomethyl moiety enhances the inhibitory effect of LA up to 2-fold (IC_50_ = 14 μM). Compounds **6m** and **6n** showed an uncompetitive inhibition with *K*_i_ values of 2.5 and 3.4 μM, respectively, where **6m** showed better potency than that of suramin (*K*_i_= 3.0 μM), which is the most potent positive inhibitor used in this study. Both **6m** and **6n** also exhibited a selectivity for PTP1B over TCPTP.

Based on our biological results, we have built a new uncompetitive inhibition docking model and MDS against PTP1B. Our findings indicate that hydrophobic interactions and interactions with electropositive regions of the unstructured domain of PTP1B are essential in maintaining the inhibitory activity of the LA derivatives against PTP1B. Our study’s affinity and enzyme kinetics results support this conclusion. Further research will be conducted to perform in vitro assays to evaluate whether **6m** and **6n** can enhance the glucose uptake rate in cells. Finally, we intend to conduct *in vivo* assays using a high-fat diet mouse model to evaluate the potential of these LA derivatives to treat diabetes and obesity.

## Data Availability

The data supporting this study’s findings are available from the corresponding authors, C.-B. F. and G.-A. M., upon reasonable request.

## Supporting Information Available

Experimental details: ^1^H and ^13^C NMR for compounds **6a**–**6o**, HSQC, HMBC, COSY and NOESY experiments for compound **6f**, HRMS data, X-ray crystallography data of intermediate 4, and additional figures and tables as noted in the text (PDF)
