# Supplementary material for: 3-Benzylaminomethyl Lithocholic Acid Derivatives Exhibited Potent and Selective Uncompetitive Inhibitory Activity Against Protein Tyrosine Phosphatase 1B (PTP1B)
Source: ACS Omega. 2024 Jul 22;9(30):33224–38. doi: 10.1021/acsomega.4c04948 (PMC11292843; doi:10.1021/acsomega.4c04948)
Supplement: Supplementary file 1 — ao4c04948_si_001.pdf [file ao4c04948_si_001.pdf]

Supporting Information

3-benzylaminomethyl lithocholic acid derivatives exhibited potent and selective uncompetitive inhibitory activity against Protein Tyrosine Phosphatase 1B (PTP1B)

María-Eugenia Mendoza-Jasso[a,b,d], Jaime Pérez-Villanueva[b], José G. Alvarado-Rodríguez[c], Martin González-Andrade[d,*], Francisco Cortés-Benítez[b,*]

[a] Doctorado en Ciencias Farmacéuticas, División de Ciencias Biológicas y de la Salud, Universidad Autónoma Metropolitana – Unidad Xochimilco, Ciudad de México 04960, Mexico.

[b] Laboratorio de Síntesis y Aislamiento de Sustancias Bioactivas, Departamento de Sistemas Biológicos, División de Ciencias Biológicas y de la Salud, Universidad Autónoma Metropolitana – Unidad Xochimilco, Ciudad de México 04960, Mexico.

[c] Área académica de Química, Universidad Autónoma del Estado de Hidalgo, Hidalgo 42184, Mexico.

[d] Laboratorio de Biosensores y Modelaje Molecular, Departamento de Bioquímica, Facultad de Medicina, Universidad Nacional Autónoma de México, Ciudad de México 04510, Mexico.

Table of content

Table S1. Electronic and lipophilic parameters for compounds 6a–6o 1

Table S2. Carbon numbering for ^{1}H NMR and ^{13}C NMR signal assignment of compound 6f 9

Table S3. Crystal data, structure solution, and refinement parameters for compound 4 40

Figure S1. Selectivity of the inhibition for PTP1B over TCPTP for LA derivatives at 400 μM 1

Figure S2. Selectivity of the inhibition for PTP1B over TCPTP for controls 2

Figure S3. 2D interacting model of LA derivatives in the unstructured region of *h*PTP1B$_{1\text{-}400}$ 3

Figure S4. 2D interacting model of Ursolic acid and TCS401 in the unstructured region of *h*PTP1B$_{1\text{-}400}$. 4

Figure S5. Standard RMSD (Å) of new LA derivatives and PTP1B+ pNPP system 4

Figure S6. Standard RMSF (Å) of new LA derivatives and PTP1B+ pNPP system 5

Figure S7. Contact matrix generated from MD simulations for LA 5

Figure S8. Contact matrix generated from MD simulations for derivative 6e 6

Figure S9. Contact matrix generated from MD simulations for derivative 6f 6

Figure S10. Contact matrix generated from MD simulations for derivative 6n 7

Figure S11. Contact matrix generated from MD simulations for derivative 6o 7
Figure S12. Contact matrix generated from MD simulations for UA 8
Figure S13. Contact matrix generated from MD simulations for TCS401 8
Figure S14. ^{1}H NMR (600 MHz) spectrum of intermediate **3** in CDCl 10
Figure S15. ^{13}C NMR (600 MHz) spectrum of intermediate **3** in $CDCl_3$ 10
Figure S16. ^{1}H NMR (600 MHz) spectrum of intermediate **4** in $CDCl_3$ 11
Figure S17. ^{13}C NMR (600 MHz) spectrum of intermediate **3** in $CDCl_3$ 11
Figure S18. ^{1}H NMR (600 MHz) spectrum of compound **6a** in Pyridine-d5 12
Figure S19. ^{13}C NMR (600 MHz) spectrum of compound **6a** in Pyridine-d5 12
Figure S20. ^{1}H NMR (600 MHz) spectrum of compound **6b** in DMSO-d6 13
Figure S21. ^{13}C NMR (600 MHz) spectrum of compound **6b** in DMSO-d6 13
Figure S22. ^{1}H NMR (600 MHz) spectrum of compound **6c** in Pyridine-d5 14
Figure S23. ^{13}C NMR (600 MHz) spectrum of compound **6c** in Pyridine-d5 14
Figure S24. ^{1}H NMR (600 MHz) spectrum of compound **6d** in Pyridine-d5 15
Figure S25. ^{13}C NMR (600 MHz) spectrum of compound **6d** in Pyridine-d5 15
Figure S26. ^{1}H NMR (500 MHz) spectrum of compound **6e** in DMSO-d6 16
Figure S27. ^{13}C NMR (500 MHz) spectrum of compound **6e** in DMSO-d6 16
Figure S28. ^{1}H NMR (500 MHz) spectrum of compound **6f** in DMSO-d6 17
Figure S29. ^{13}C NMR (500 MHz) spectrum of compound **6f** in DMSO-d6 17
Figure S30. HSQC spectra of 6f 18
Figure S31. HMBC spectra of 6f. 19
Figure S32. COSY spectra of 6f 20
Figure S33. NOESY spectra of 6f 21
Figure S34. ^{1}H NMR (600 MHz) spectrum of compound **6g** in Pyridine-d5 22
Figure S35. ^{13}C NMR (600 MHz) spectrum of compound **6g** in Pyridine-d5 22
Figure S36. ^{1}H NMR (600 MHz) spectrum of compound **6h** in DMSO-d6 23
Figure S37. ^{13}C NMR (600 MHz) spectrum of compound **6h** in DMSO-d6 23

Figure S38. ^{1}H NMR (600 MHz) spectrum of compound **6i** in Pyridine-d5 24
Figure S39. ^{13}C NMR (600 MHz) spectrum of compound **6i** in Pyridine-d5 24
Figure S40. ^{1}H NMR (600 MHz) spectrum of compound **6j** in Pyridine-d5 25
Figure S41. ^{13}C NMR (600 MHz) spectrum of compound **6j** in Pyridine-d5 25
Figure S42. ^{1}H NMR (600 MHz) spectrum of compound **6k** in DMSO-d6 26
Figure S43. ^{13}C NMR (600 MHz) spectrum of compound **6k** in DMSO-d6 26
Figure S44. ^{1}H NMR (600 MHz) spectrum of compound **6l** in DMSO-d6 27
Figure S45. ^{13}C NMR (600 MHz) spectrum of compound **6l** in DMSO-d6 27
Figure S46. ^{1}H NMR (600 MHz) spectrum of compound **6m** in Pyridine-d5 28
Figure S47. ^{13}C NMR (600 MHz) spectrum of compound **6m** in Pyridine-d5 28
Figure S48. ^{1}H NMR (600 MHz) spectrum of compound **6n** in DMSO-d6 29
Figure S49. ^{13}C NMR (600 MHz) spectrum of compound **6n** in DMSO-d6 29
Figure S50. ^{1}H NMR (500 MHz) spectrum of compound **6o** in DMSO-d6 30
Figure S51. ^{13}C NMR (500 MHz) spectrum of compound **6o** in DMSO-d6 30
Figure S52. Mass spectrum formula report of compound 4 31
Figure S53. Mass spectrum formula report of compound 6a 31
Figure S54. Mass spectrum formula report of compound 6b 32
Figure S55. Mass spectrum formula report of compound 6c 32
Figure S56. Mass spectrum formula report of compound 6d 33
Figure S57. Mass spectrum formula report of compound 6e 33
Figure S58. Mass spectrum formula report of compound 6f 34
Figure S59. Mass spectrum formula report of compound 6g 34
Figure S60. Mass spectrum formula report of compound 6h 35
Figure S61. Mass spectrum formula report of compound 6i 35
Figure S62. Mass spectrum formula report of compound 6j 36
Figure S63. Mass spectrum formula report of compound 6k 37
Figure S64. Mass spectrum formula report of compound 6l 37

Figure S65. Mass spectrum formula report of compound 6m 38

Figure S66. Mass spectrum formula report of compound 6n 38

Figure S67. Mass spectrum formula report of compound 6o 39

Table S1. Electronic and lipophilic parameters for compounds **6a–6o**.

Compound	R	[a] π	[a] σ_m	[a] σ_p	[a] E*s*	[b] MR	[b] Log P
6a	H	0.0	0.0	0.0	0.0	155.35	4.86
6b	4’-OH	-0.67	---	-0.37	-0.55	157.37	4.52
6c	3’-OCH_3	-0.02	0.12	---	-0.55	161.84	4.79
6d	4’-OCH_3	-0.02	---	-0.27	-0.55	161.84	4.79
6e	3’-CH_3	0.56	-0.07	---	-1.24	160.31	5.14
6f	4’-CH_3	0.56	---	-0.17	-1.24	160.31	5.14
6g	3’-F	0.14	0.34	---	-0.46	155.30	5.15
6h	4’-F	0.14	---	0.06	-0.46	155.30	5.15
6i	3’-Cl	0.71	0.37	---	-0.97	160.36	5.33
6j	4’-Cl	0.71	---	0.23	-0.97	160.36	5.33
6k	3’-COOH	-0.32	0.37	---	---	162.30	4.51
6l	4’-COOH	-0.32	---	0.45	---	162.30	4.51
6m	3’-CF_3	0.88	0.43	---	-2.40	160.35	5.83
6n	4’-CF_3	0.88	---	0.54	-2.40	160.35	5.83
6o	3’,5’-bis-CF_3	1.76	0.86	---	-4.80	165.35	6.81

[a] Wired chemist: https://www.wiredchemist.com/chemistry/data [b] SwissADME: http://www.swissadme.ch/

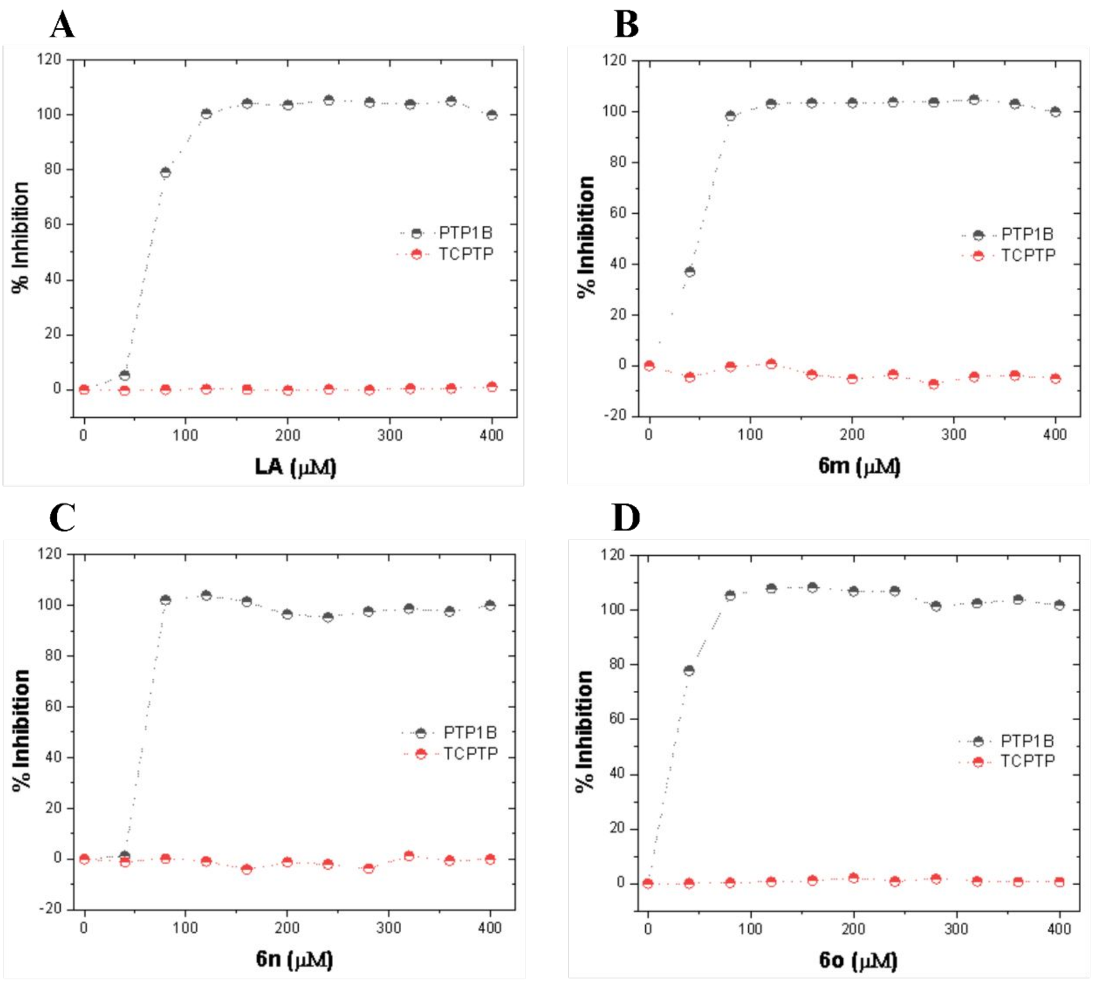

Figure S1. Selectivity of the inhibition for PTP1B over TCPTP for LA derivatives at 400 μM. **(A)** Lithocholic Acid; **(B)** Compound 6m; **(C)** Compound 6n and **(D)** Compound 6o.

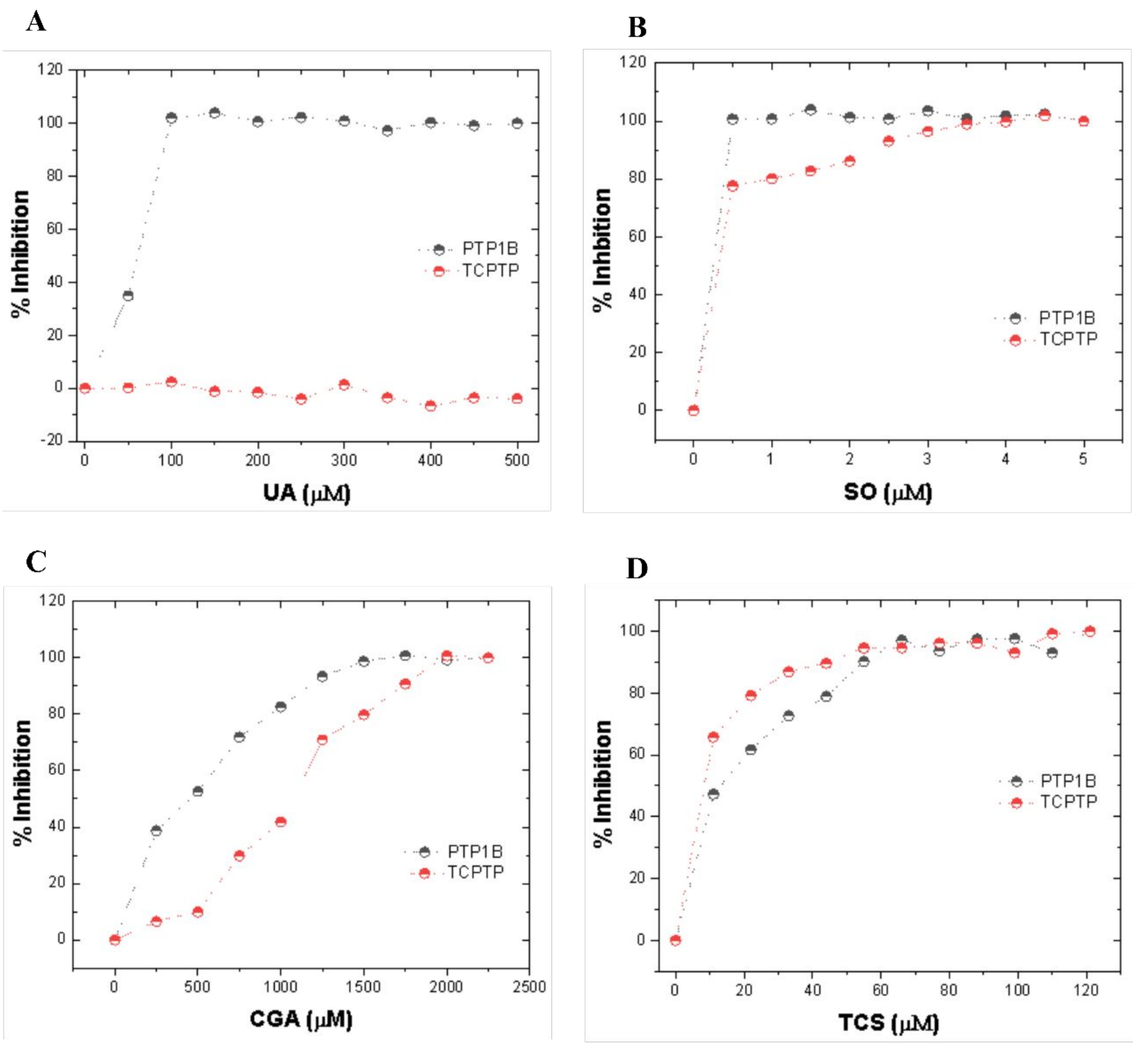

Figure S2. Selectivity of the inhibition for PTP1B over TCPTP for (**A**) Ursolic acid; (**B**) Sodium Orthovanadate; (**C**) Chlorogenic acid and (**D**) TCS401.

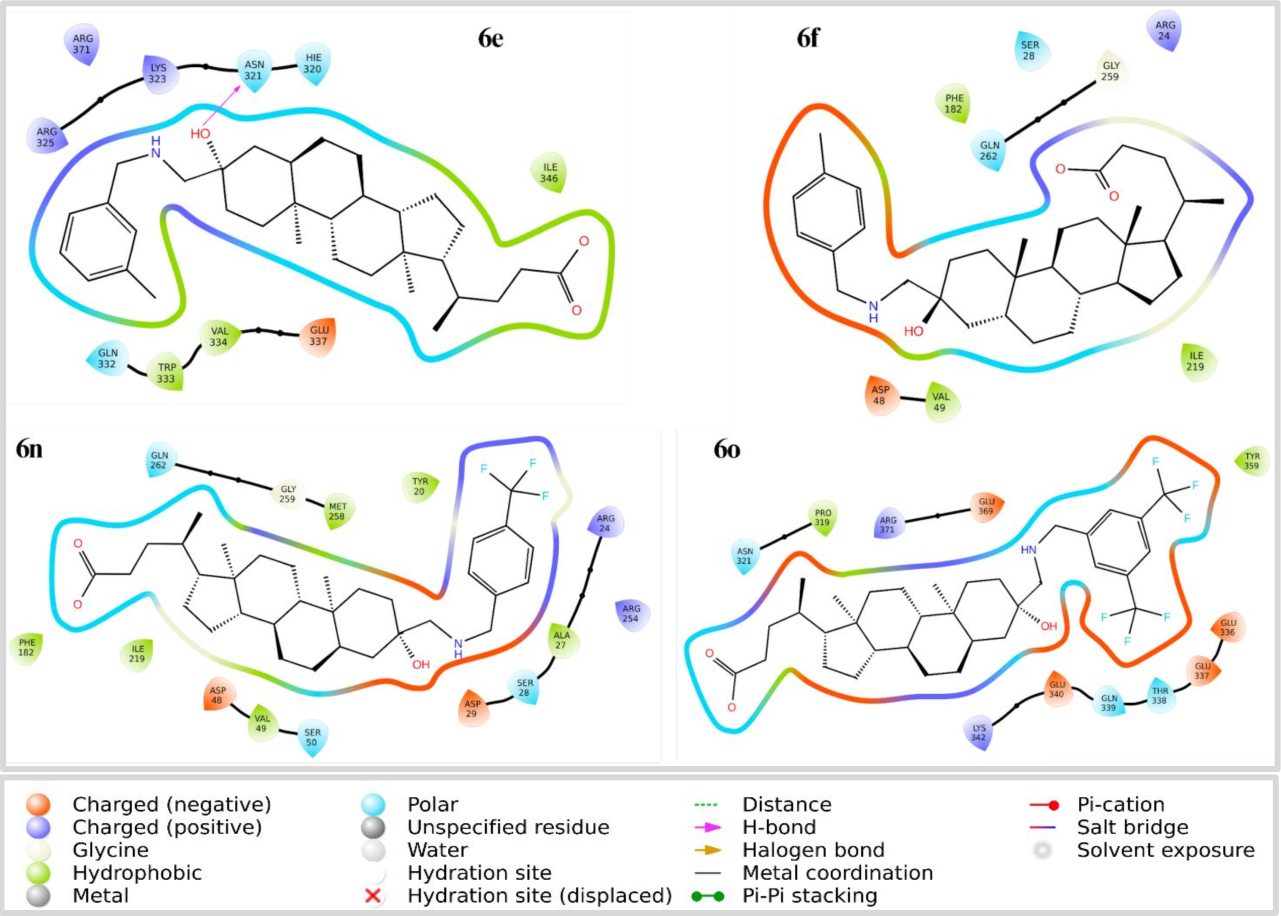

Figure S3. 2D interacting model of LA derivatives. 6e, 6f, 6n, and 6o with amino acid residues in the unstructured region of *h*PTP1B$_{1-400}$. The analysis of the interactions with residues at 4 Å is shown on the periphery.

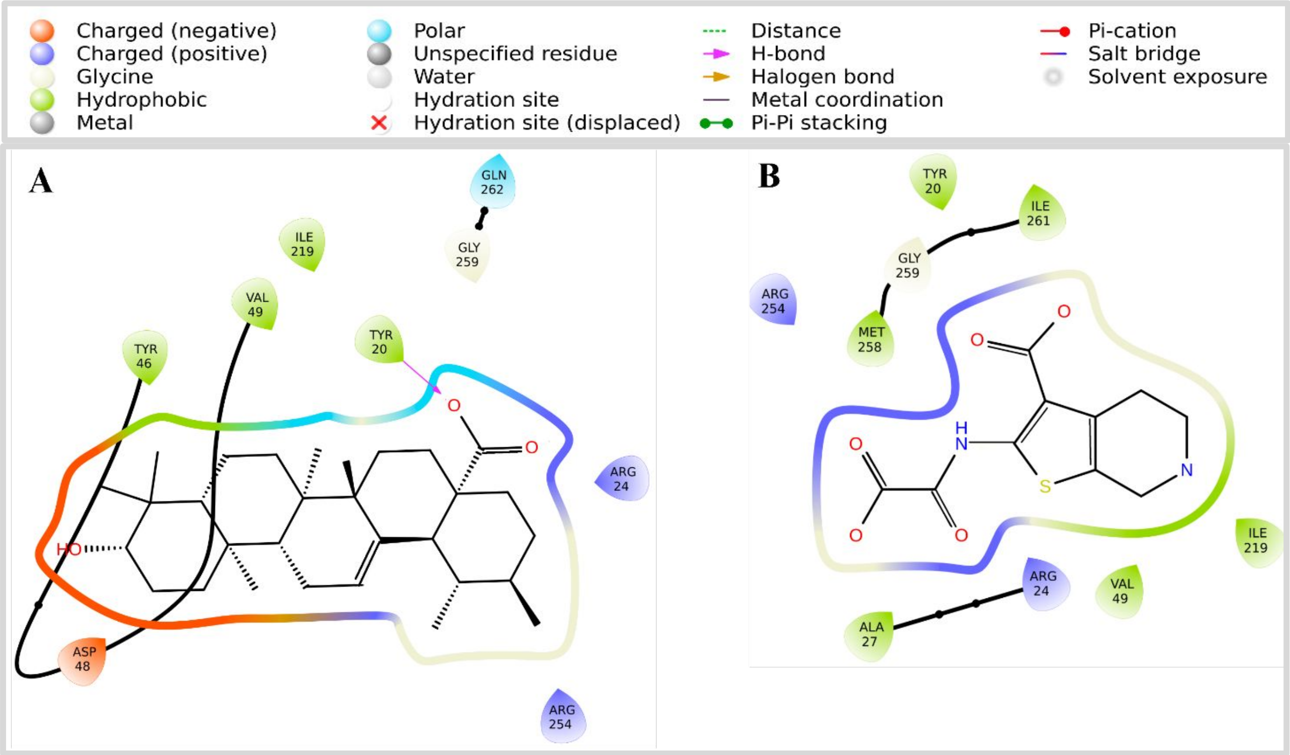

Figure S4. 2D interacting model of (A) Ursolic acid and (B) TCS401 with amino acid residues in the unstructured region of *h*PTP1B$_{1\text{-}400}$. The analysis of the interactions with residues at 4 Å is shown on the periphery.

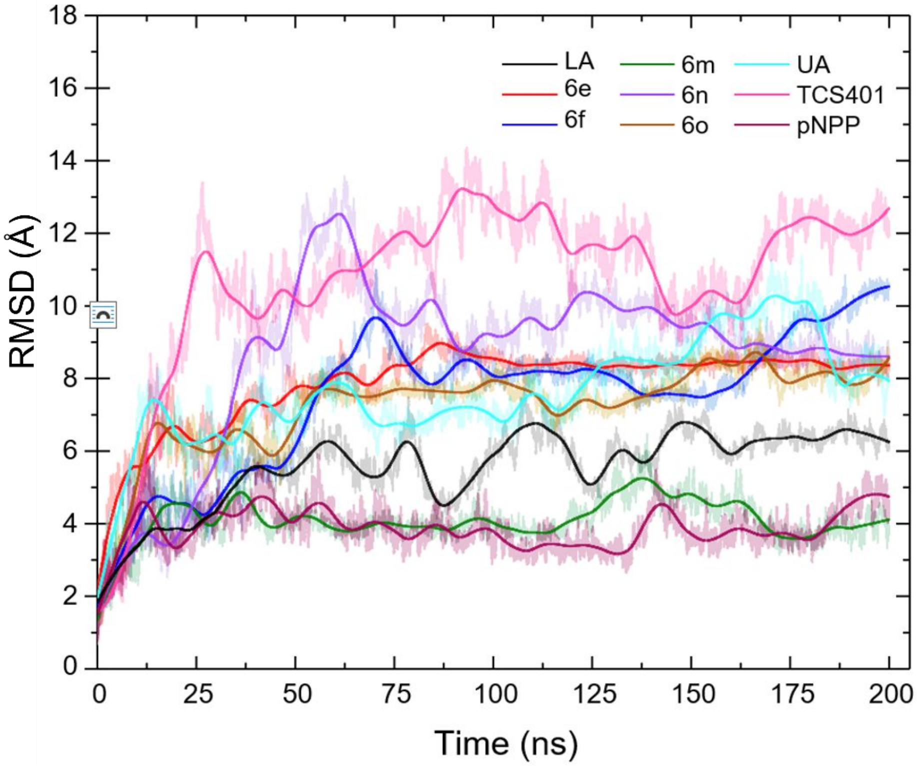

Figure S5. Standard RMSD (Å) of new LA derivatives and PTP1B+ pNPP system.

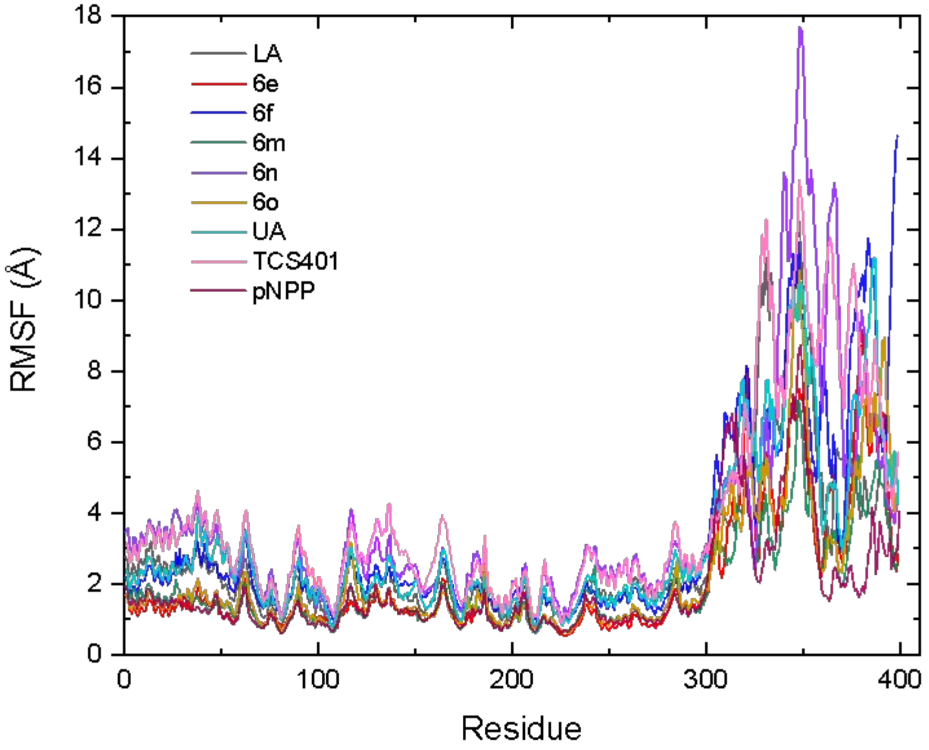

Figure S6. Standard RMSF (Å) of new LA derivatives and PTP1B+ pNPP system.

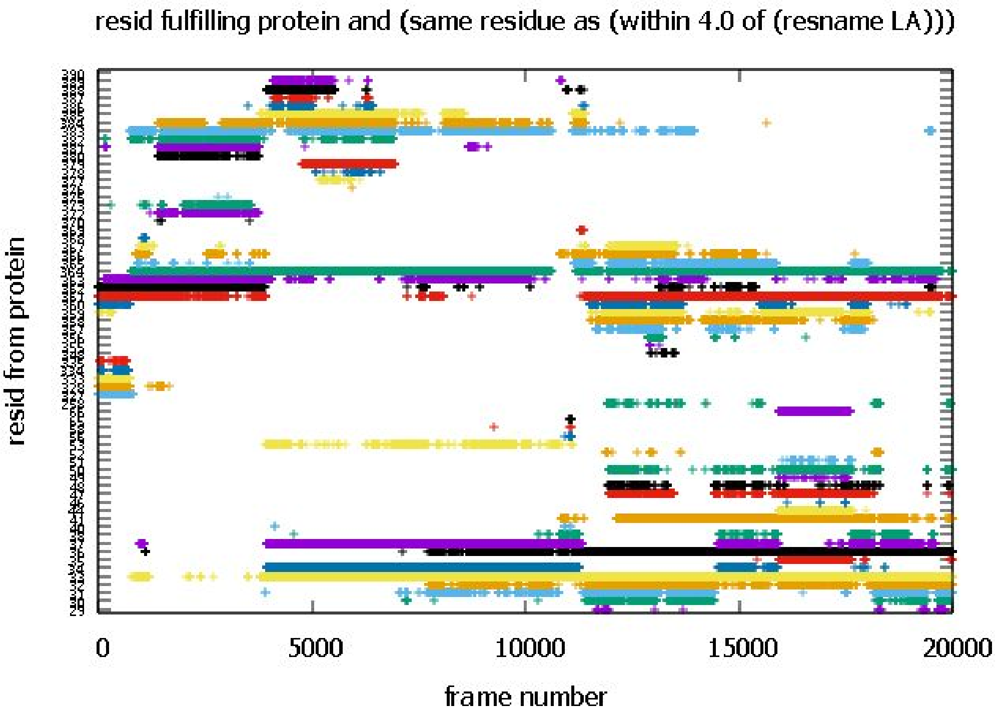

Figure S7. Contact matrix generated from MD simulations for LA.

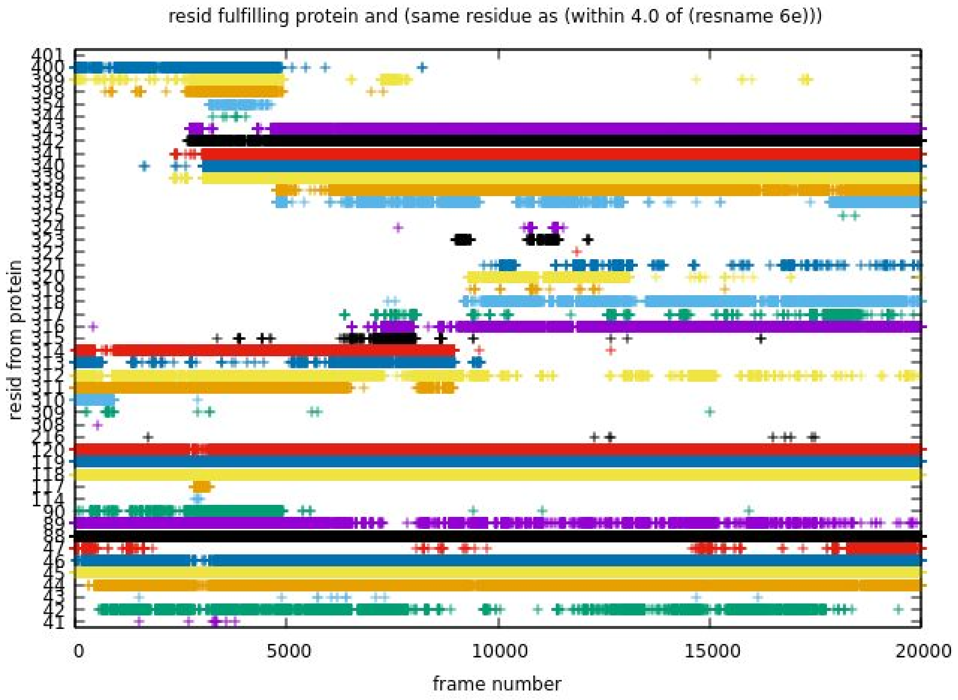

Figure S8. Contact matrix generated from MD simulations for derivative 6e.

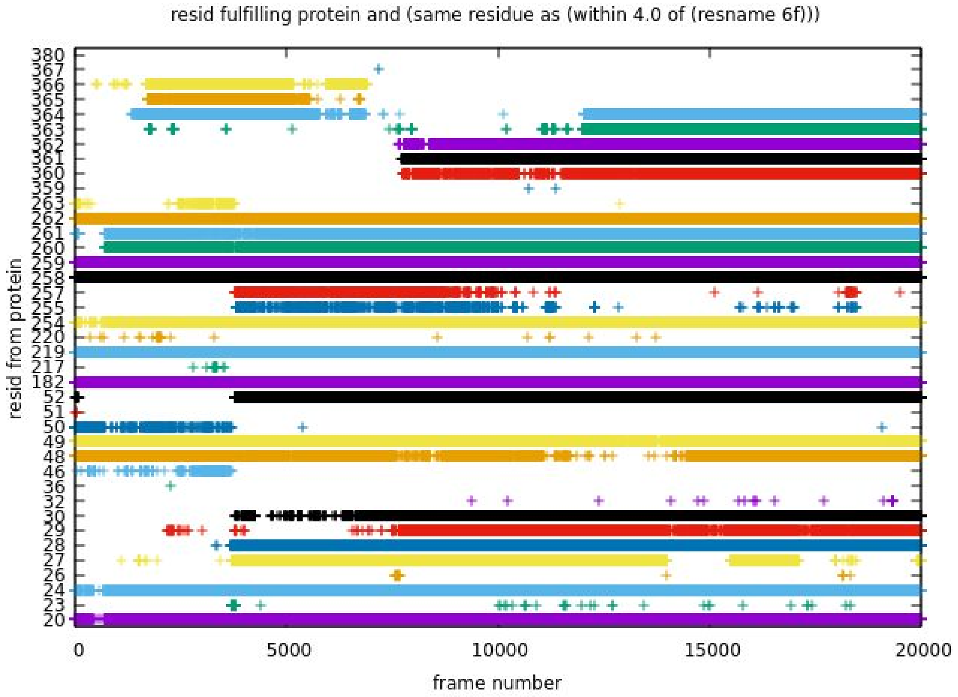

Figure S9. Contact matrix generated from MD simulations for derivative 6f.

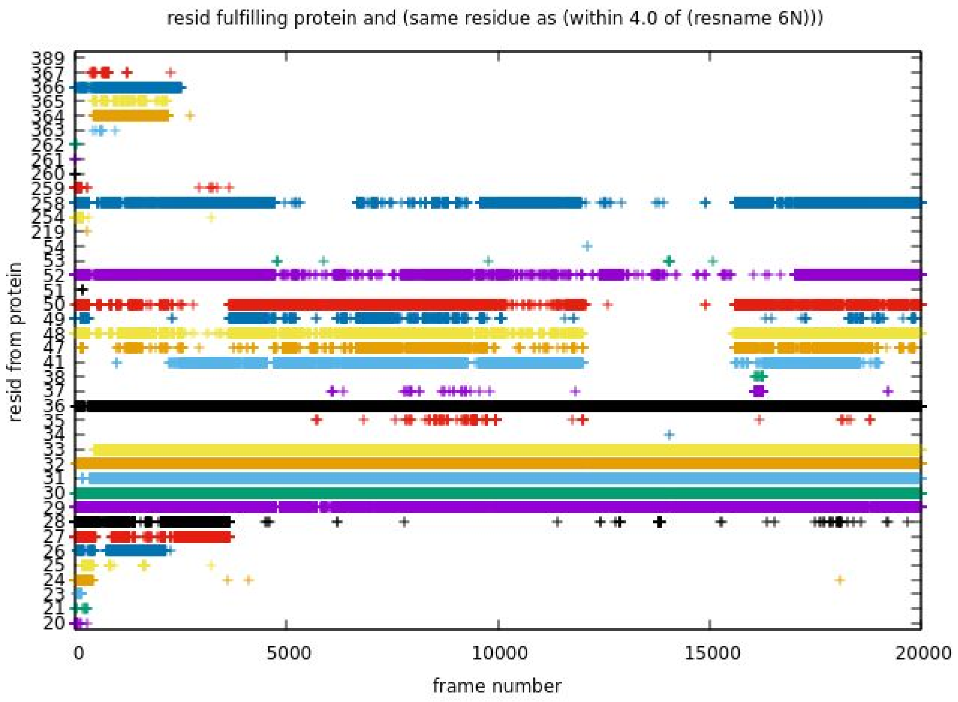

Figure S10. Contact matrix generated from MD simulations for derivative 6n.

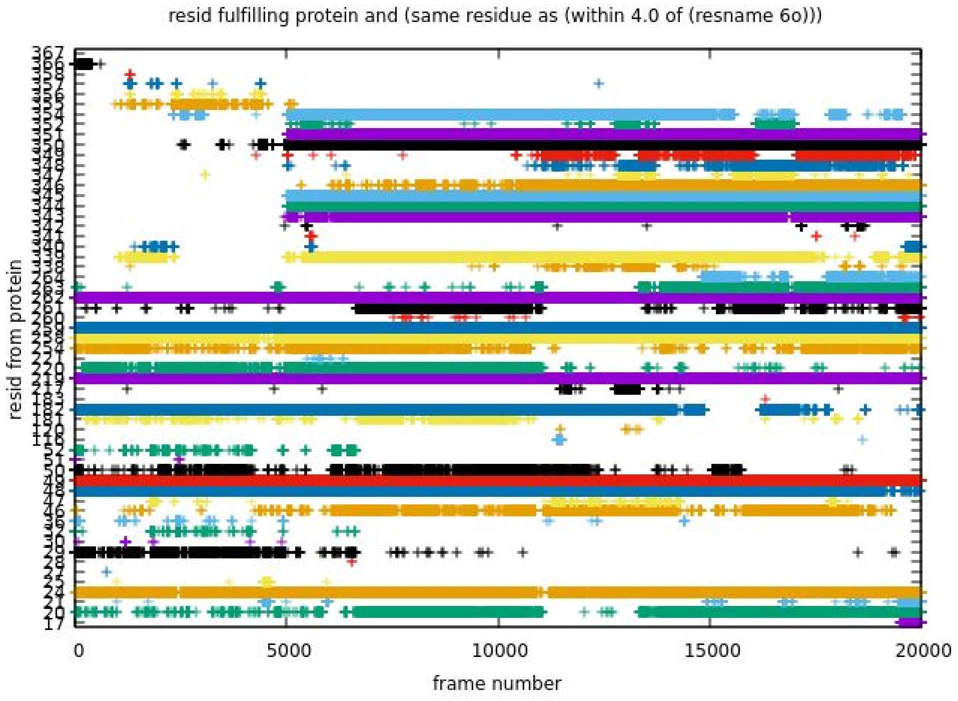

Figure S11. Contact matrix generated from MD simulations for derivative 6o.

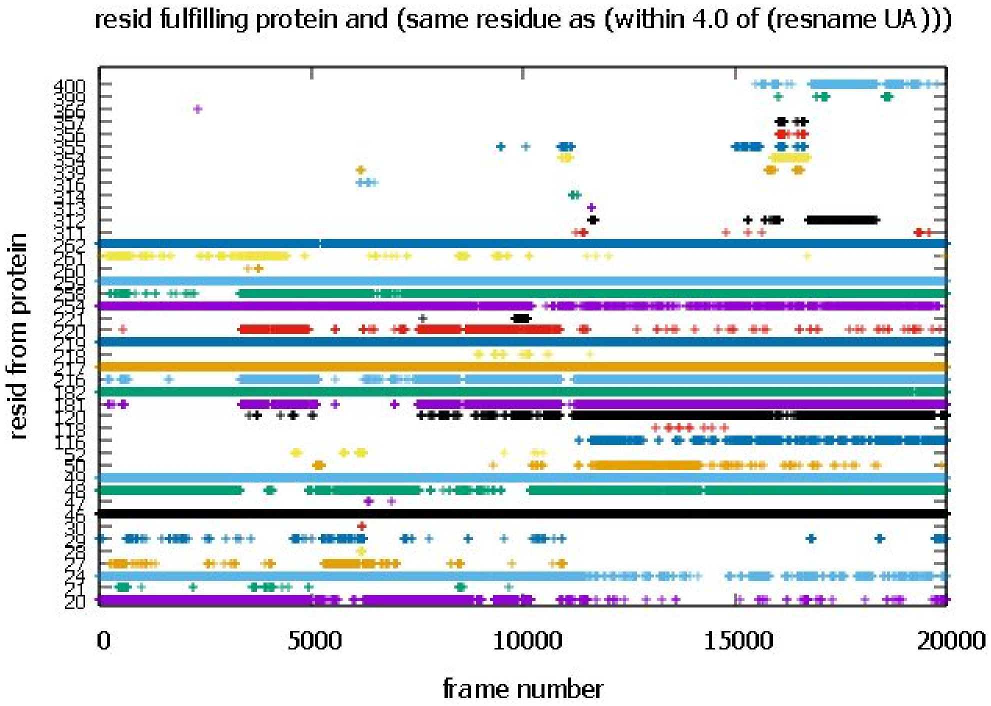

Figure S12. Contact matrix generated from MD simulations for UA.

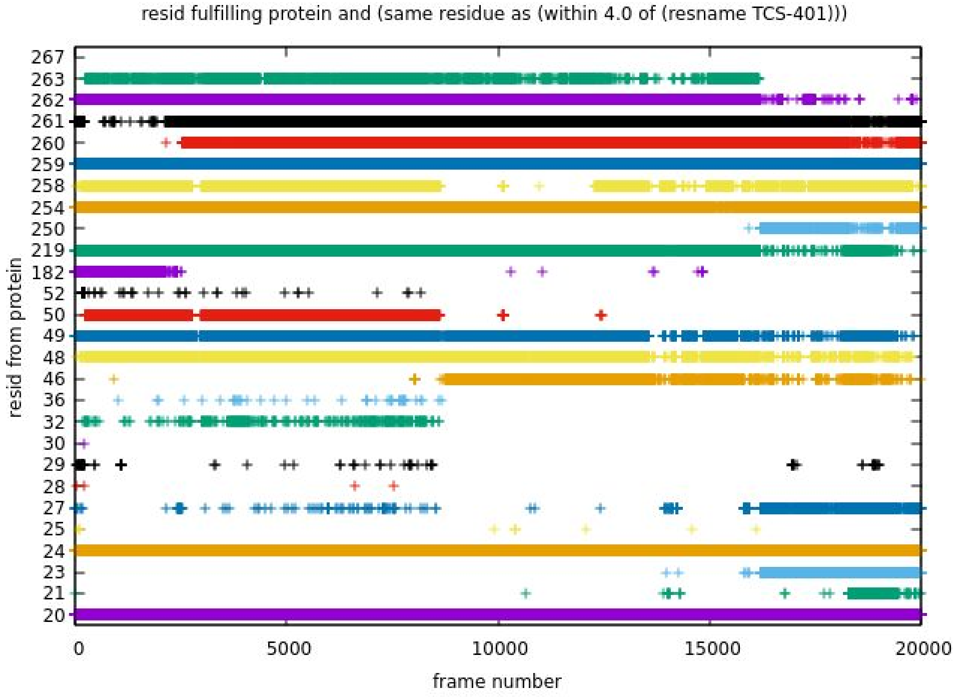

Figure S13. Contact matrix generated from MD simulations for derivative TCS401

Table S2. Carbon numbering for ^{1}H NMR and ^{13}C NMR signal assignment of compound 6f.

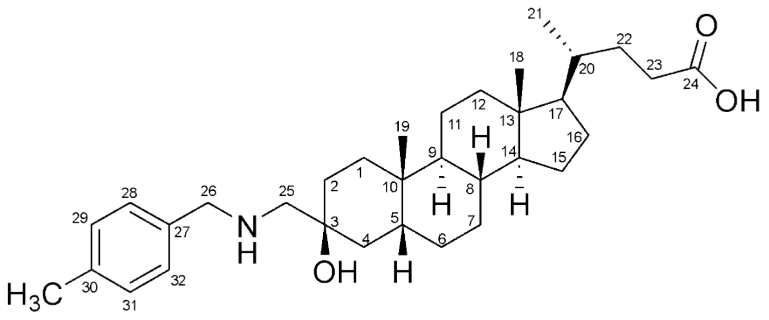

#	Type	Carbon	Proton	
			α	β
1	CH_2	30.55	1.69	1.16
2	CH_2	35.13	1.68	1.17
3	C	68.79	-	-
4	CH_2	29.29	1.33	1.25
5	CH	38.77	-	1.29
6	CH_2	26.27	1.01	1.81
7	CH_2	25.77	1.09	1.39
8	CH	34.95	-	1.30
9	CH	36.90	1.65	-
10	C	34.19	-	-
11	CH_2	20.63	1.28	1.14
12	CH_2	39.33	1.13	1.90
13	C	42.24	-	-
14	CH	55.60	1.03	-
15	CH_2	23.74	1.02	1.50
16	CH_2	27.66	1.77	1.22
17	CH	55.76	1.17	-
18	CH_3	11.81	0.60	
19	CH_3	23.28	0.90	
20	CH	34.77	1.33	-
21	CH_3	18.12	0.84	
22	CH_2	30.66	1.31	1.42
23	CH_2	30.70	2.21	2.10
24	COOH	174.81	11.92	
25	CH_2	56.66	2.70	
26	CH_2	50.49	4.07	
27	Ar-C	128.37	-	
28 and 32	Ar-CH	129.12	7.45	
29 and 31	Ar-CH	130.41	7.23	
30	Ar-C	138.29	-	
Ar-CH_3	Ar-CH_3	20.78	2.31	
3-OH	OH	-		4.84
-NH_2-	NH_2	-	9.02	

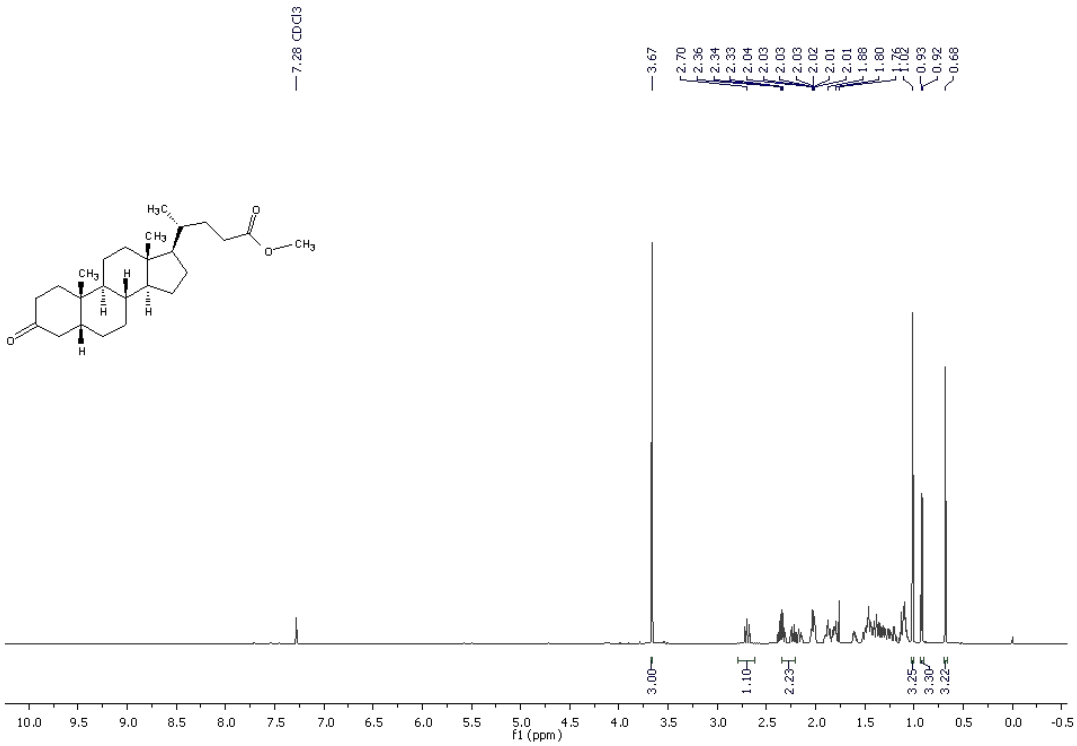

Figure S14. ^{1}H NMR (600 MHz) spectrum of intermediate **3** in $CDCl_3$

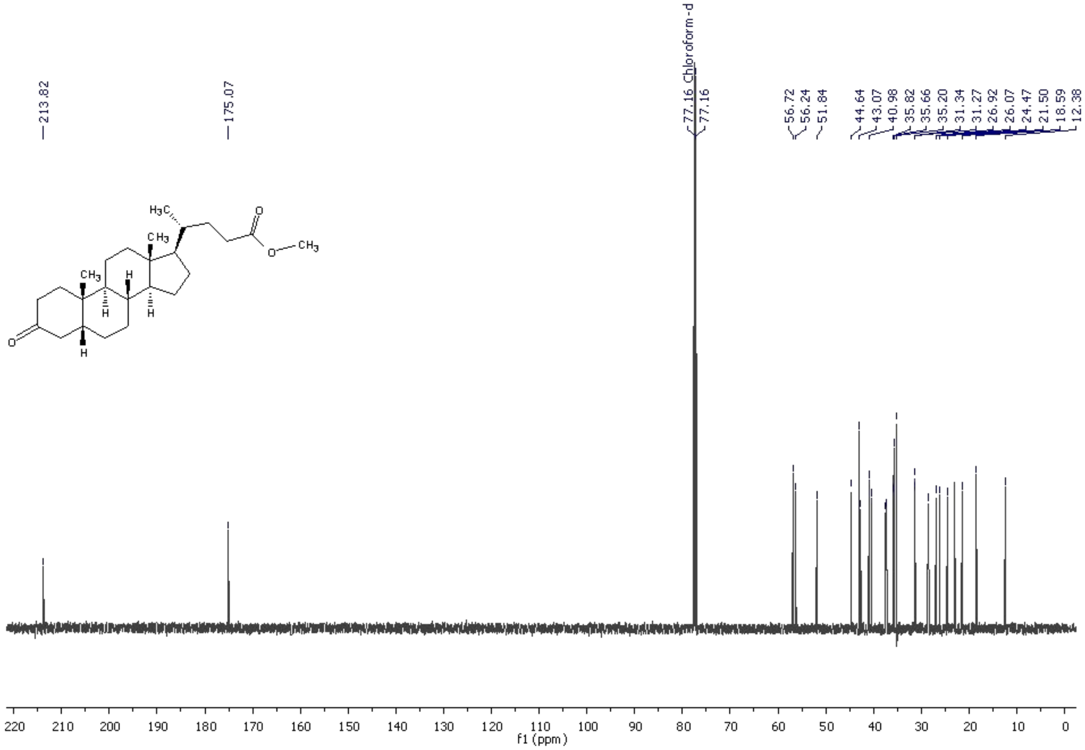

Figure S15. ^{13}C NMR (600 MHz) spectrum of intermediate **3** in $CDCl_3$

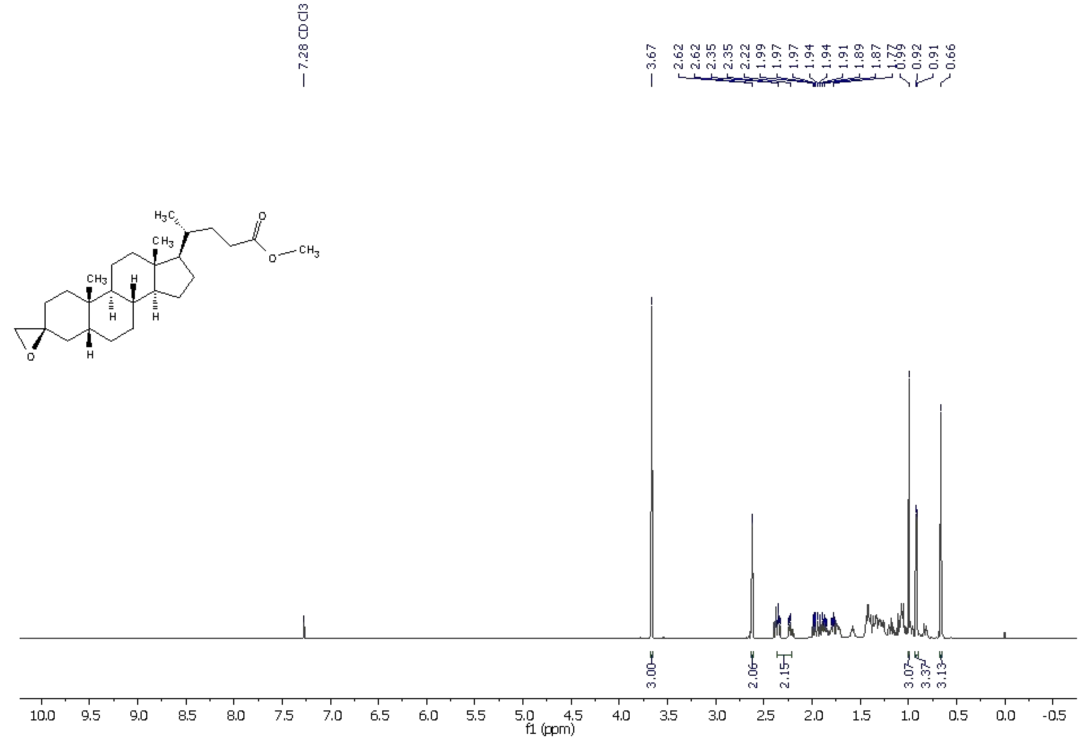

Figure S16. ^{1}H NMR (600 MHz) spectrum of intermediate **4** in $CDCl_3$

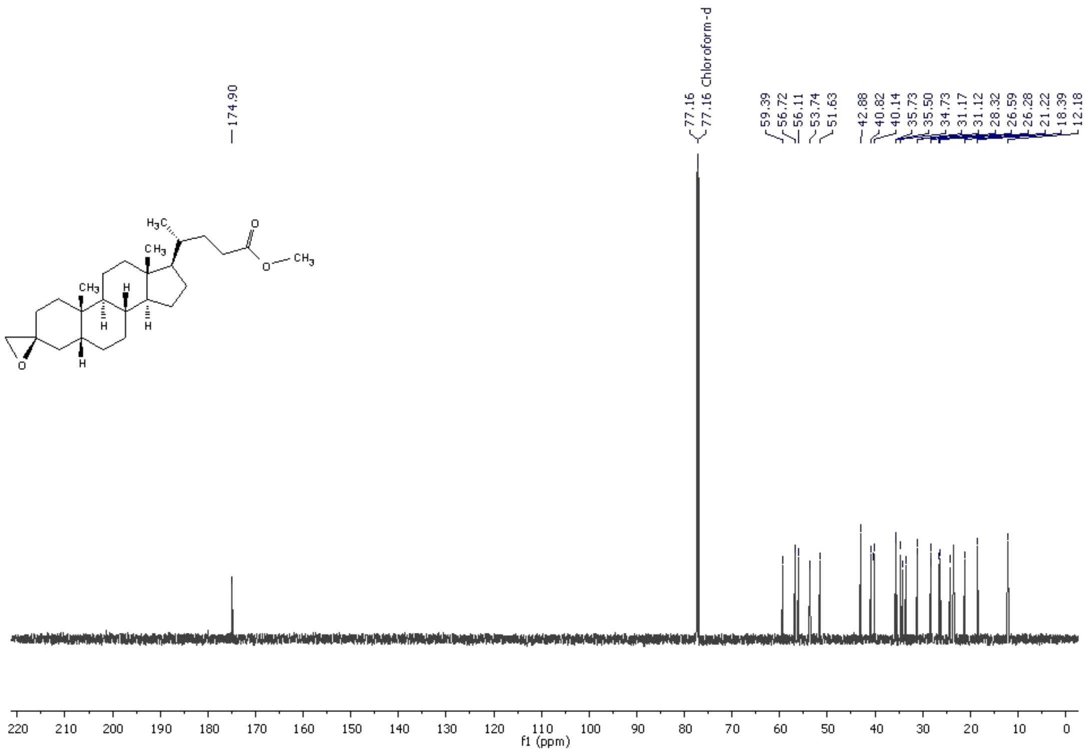

Figure S17. ^{13}C NMR (600 MHz) spectrum of intermediate **3** in $CDCl_3$

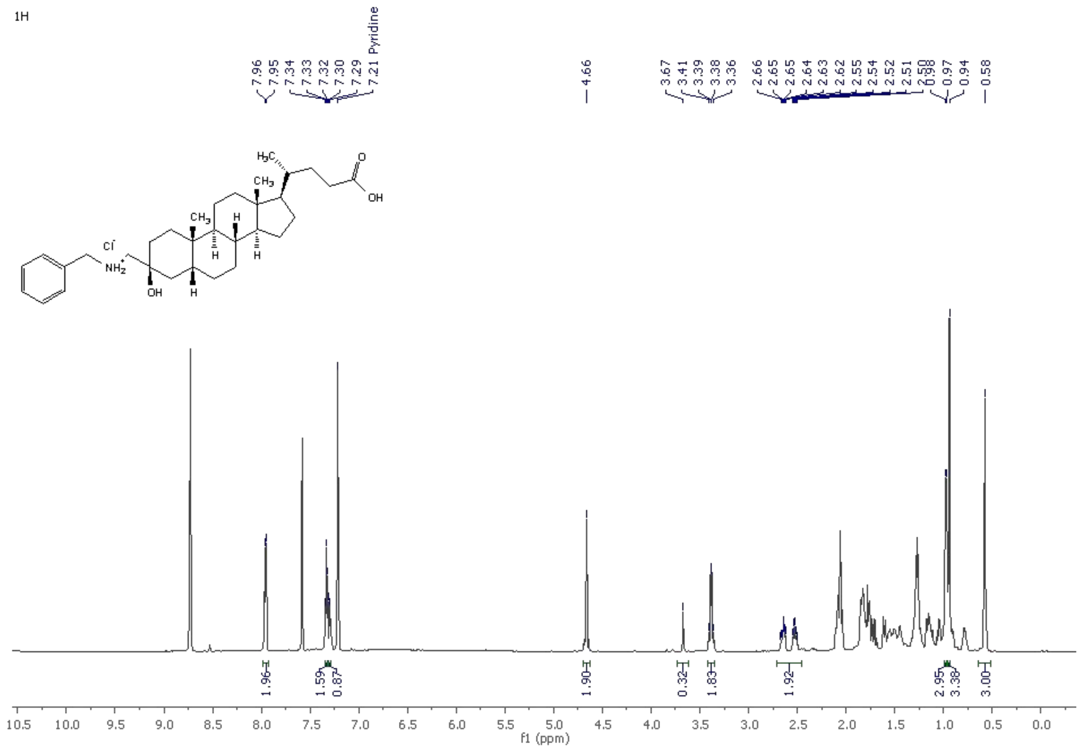

Figure S18. ^{1}H NMR (600 MHz) spectrum of compound **6a** in Pyridine-d5

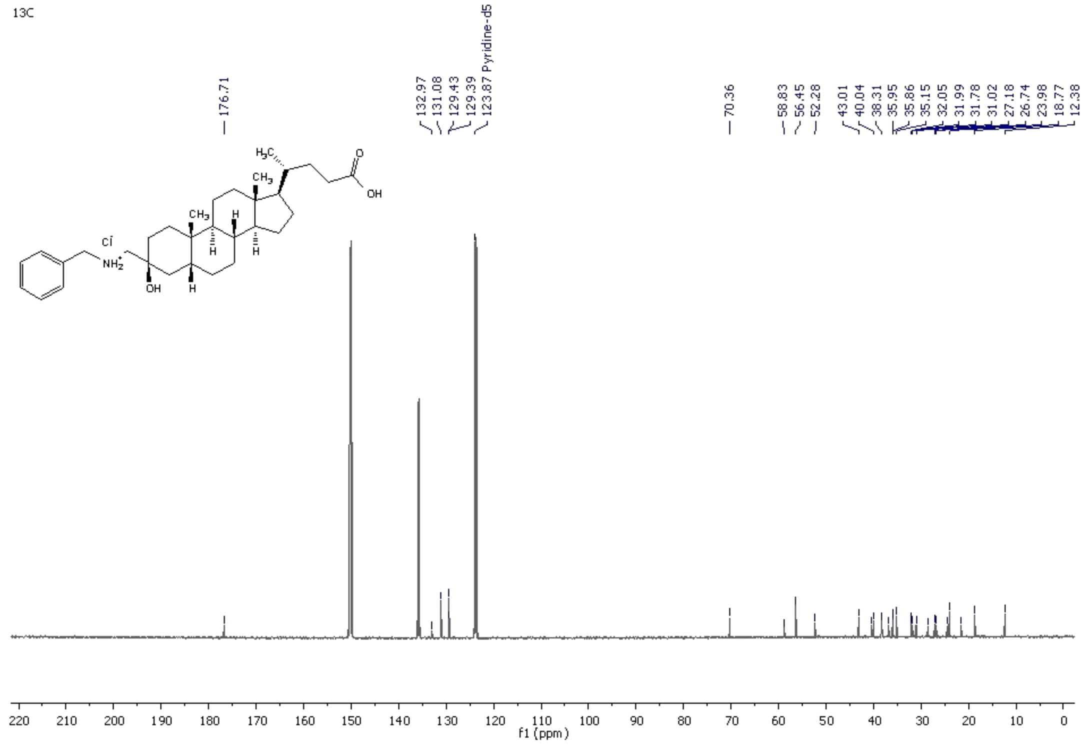

Figure S19. ^{13}C NMR (600 MHz) spectrum of compound **6a** in Pyridine-d5

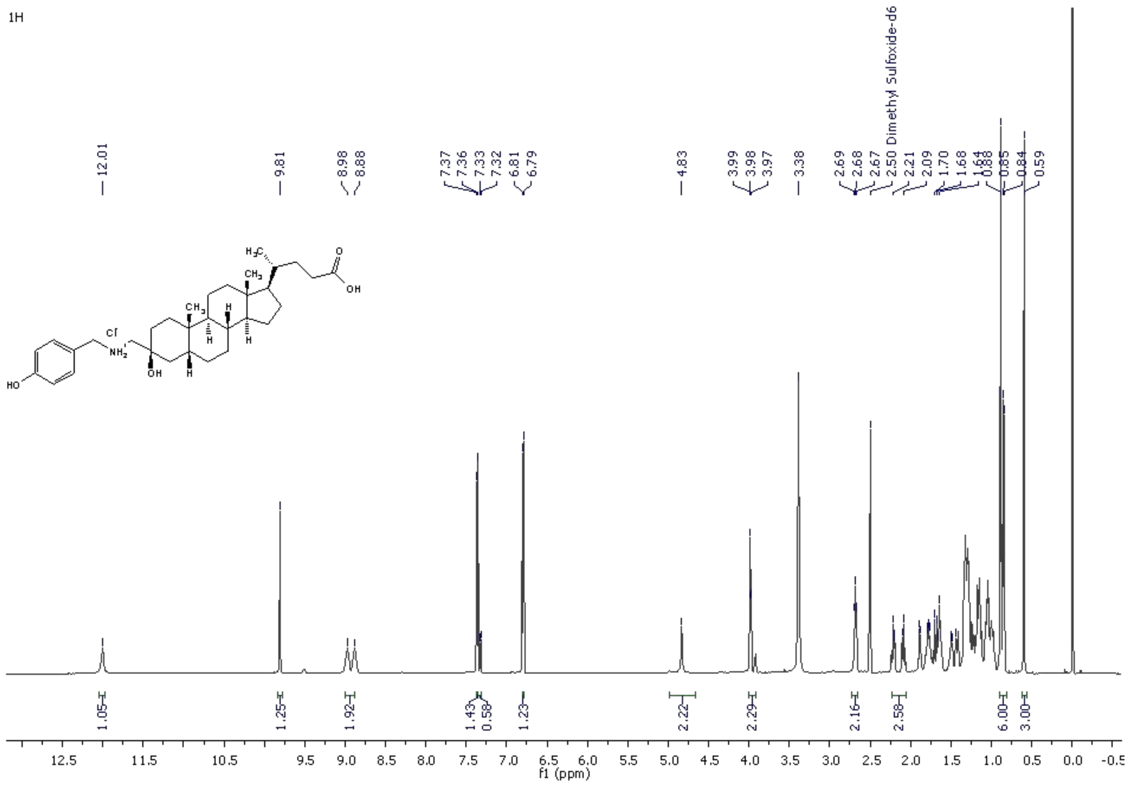

Figure S20. ^{1}H NMR (600 MHz) spectrum of compound **6b** in DMSO-d6

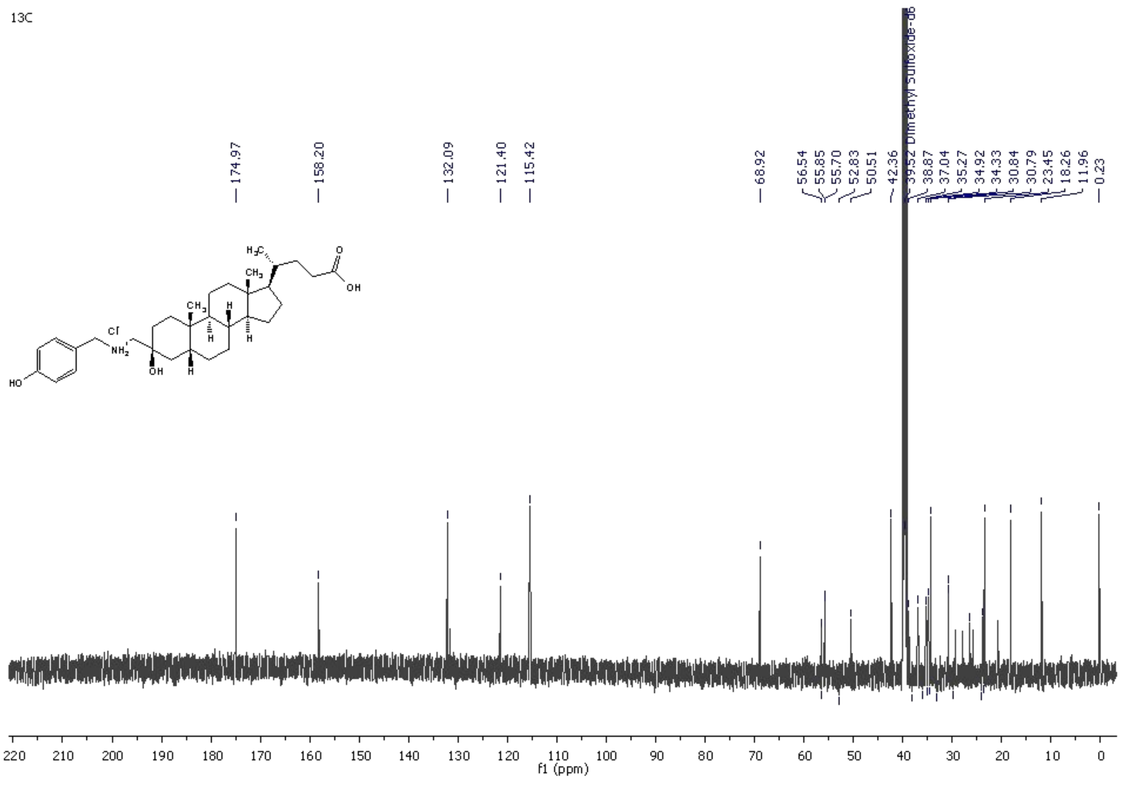

Figure S21. ^{13}C NMR (600 MHz) spectrum of compound **6b** in DMSO-d6

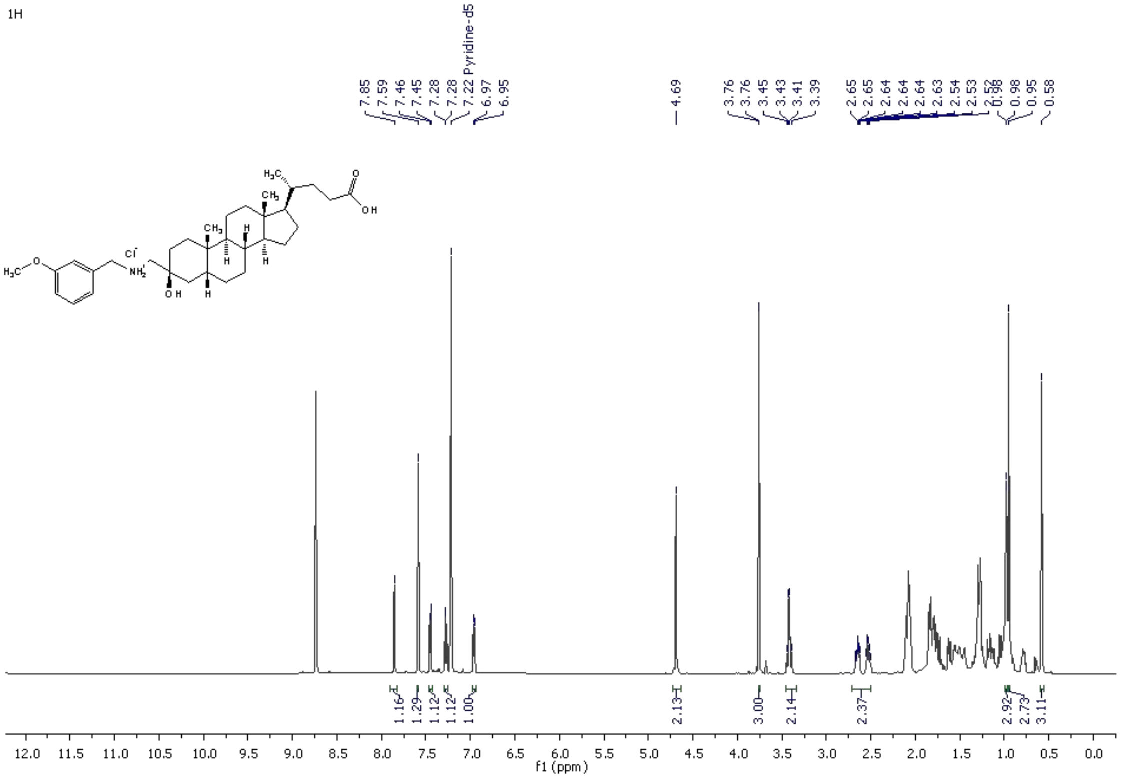

Figure S22. ^{1}H NMR (600 MHz) spectrum of compound **6c** in Pyridine-d5

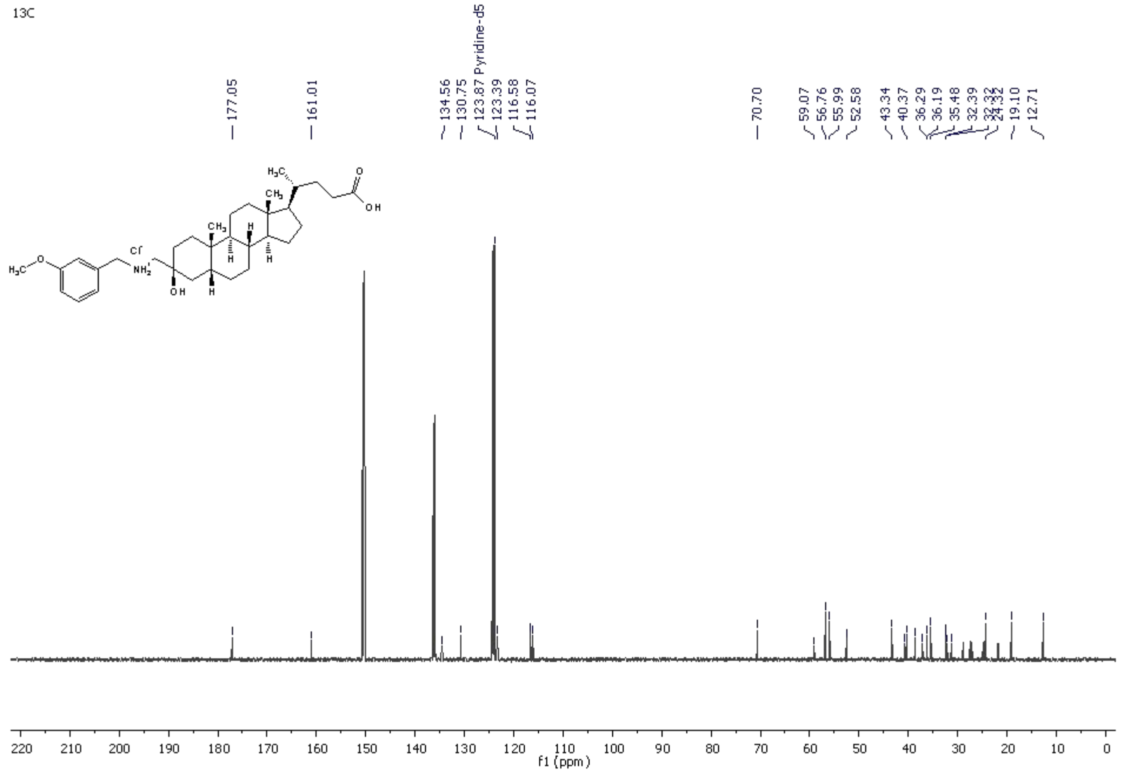

Figure S23. ^{13}C NMR (600 MHz) spectrum of compound **6c** in Pyridine-d5

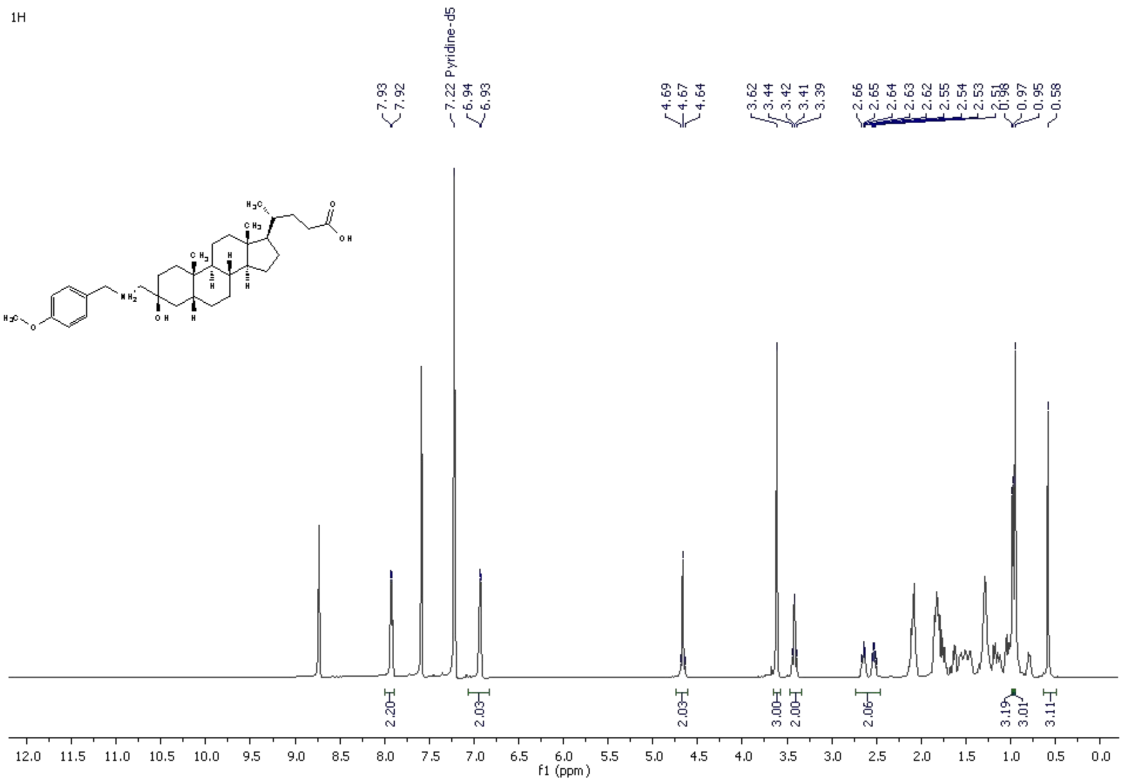

Figure S24. ^{1}H NMR (600 MHz) spectrum of compound **6d** in Pyridine-d5

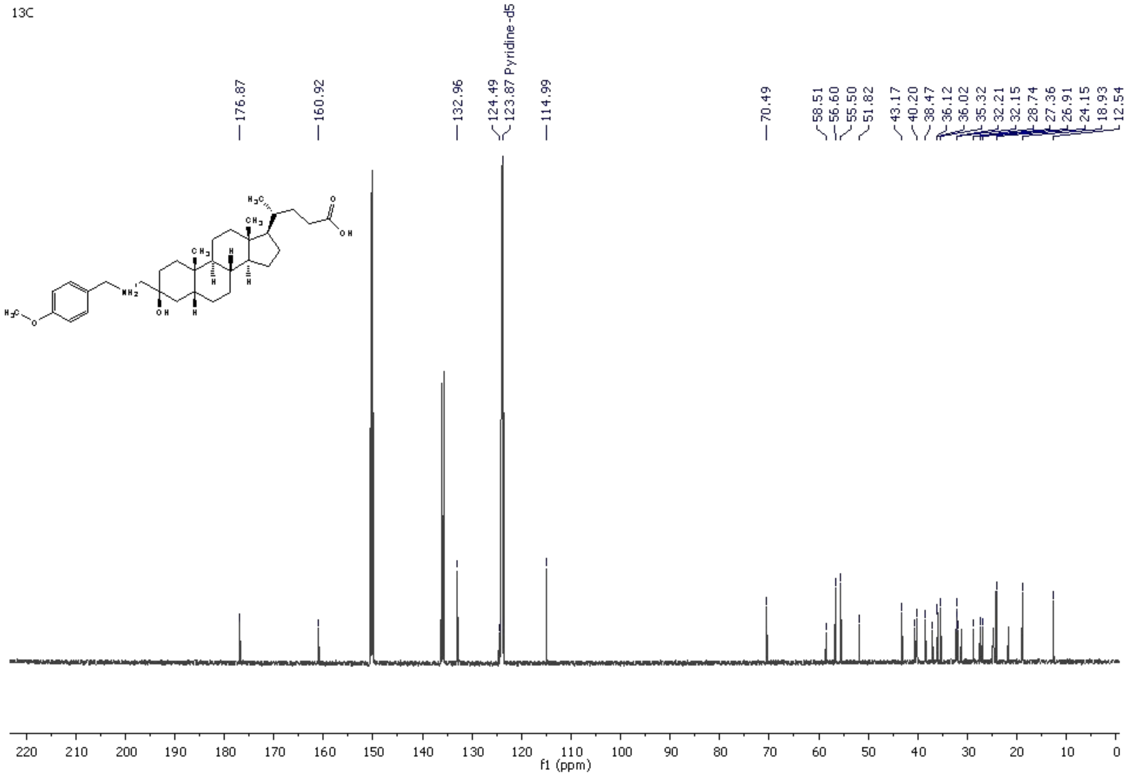

Figure S25. ^{13}C NMR (600 MHz) spectrum of compound **6d** in Pyridine-d5

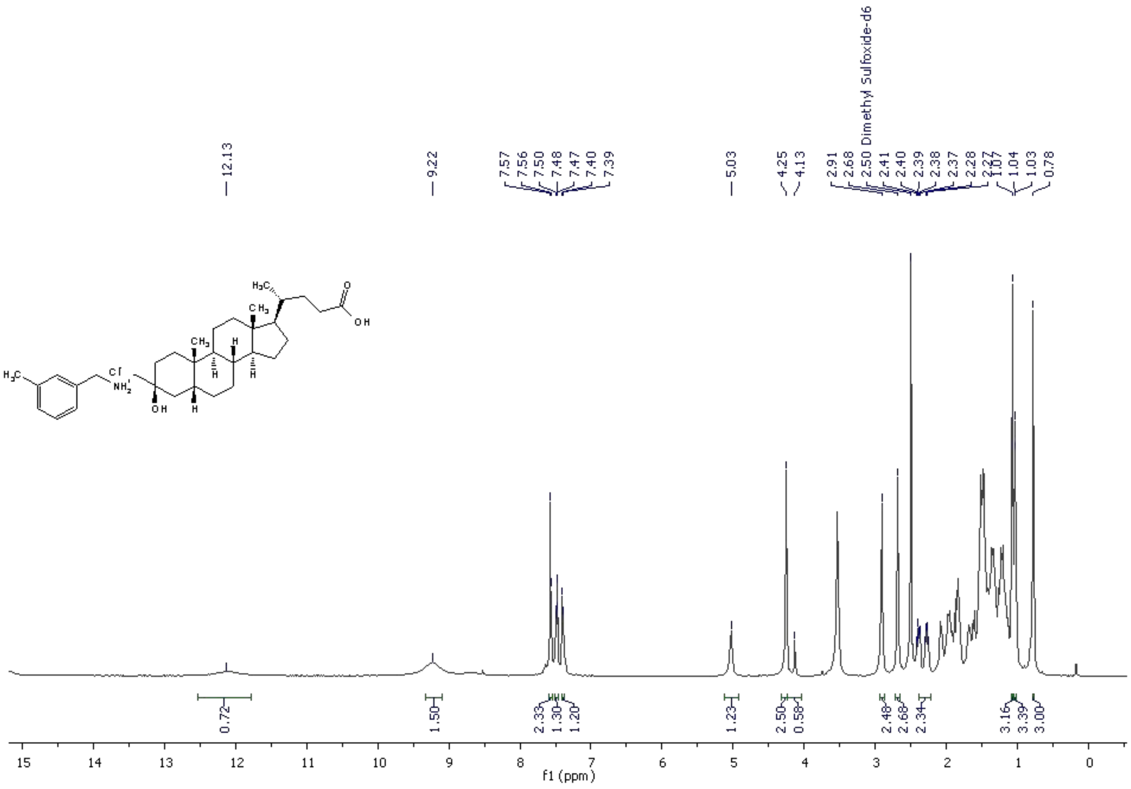

Figure S26. ^{1}H NMR (500 MHz) spectrum of compound **6e** in DMSO-d6

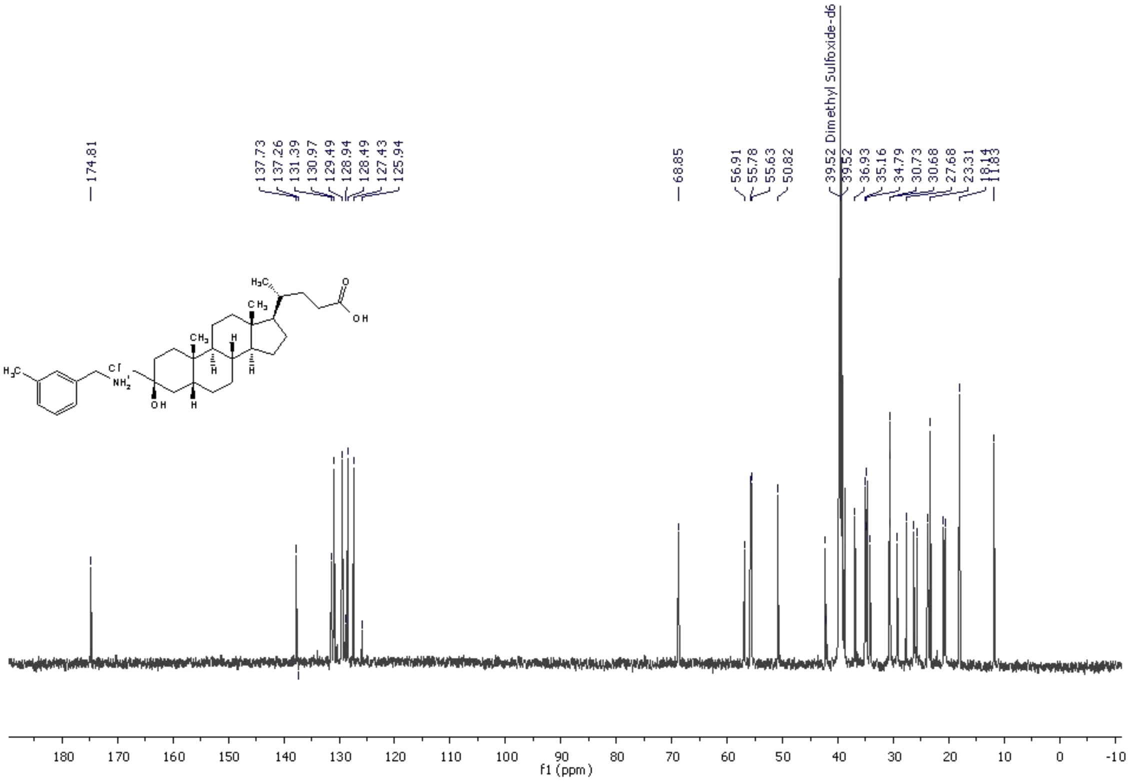

Figure S27. ^{13}C NMR (500 MHz) spectrum of compound **6e** in DMSO-d6

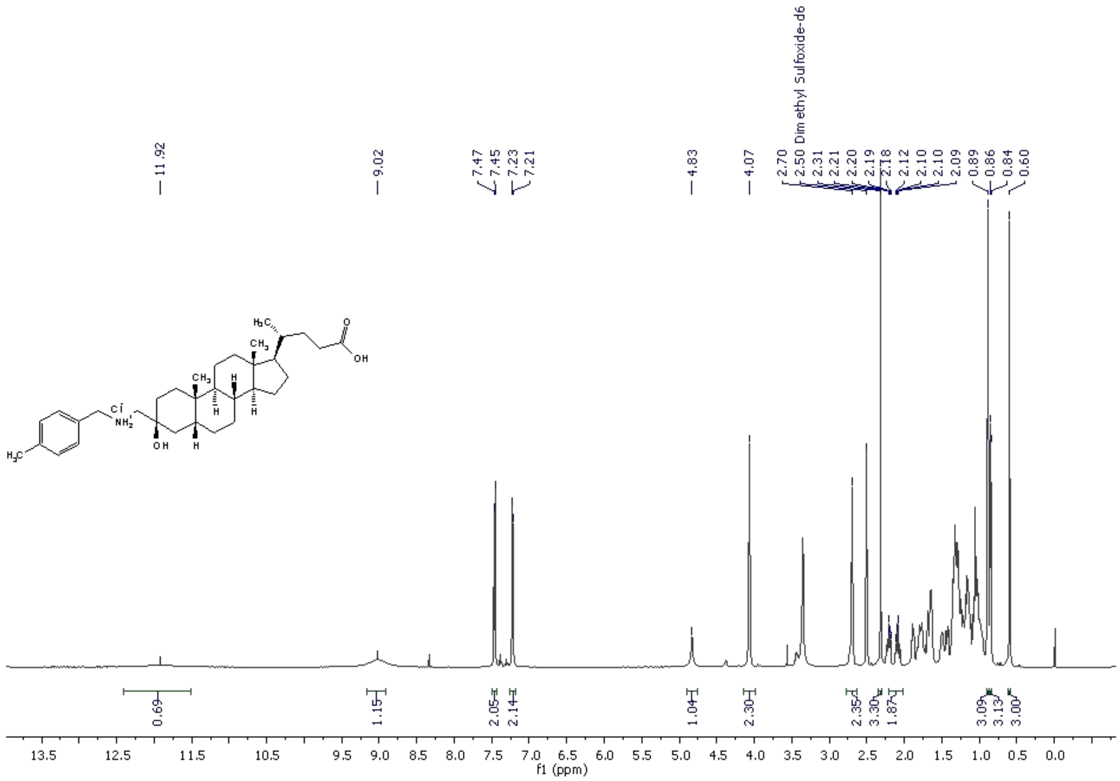

Figure S28. ^{1}H NMR (500 MHz) spectrum of compound **6f** in DMSO-d6

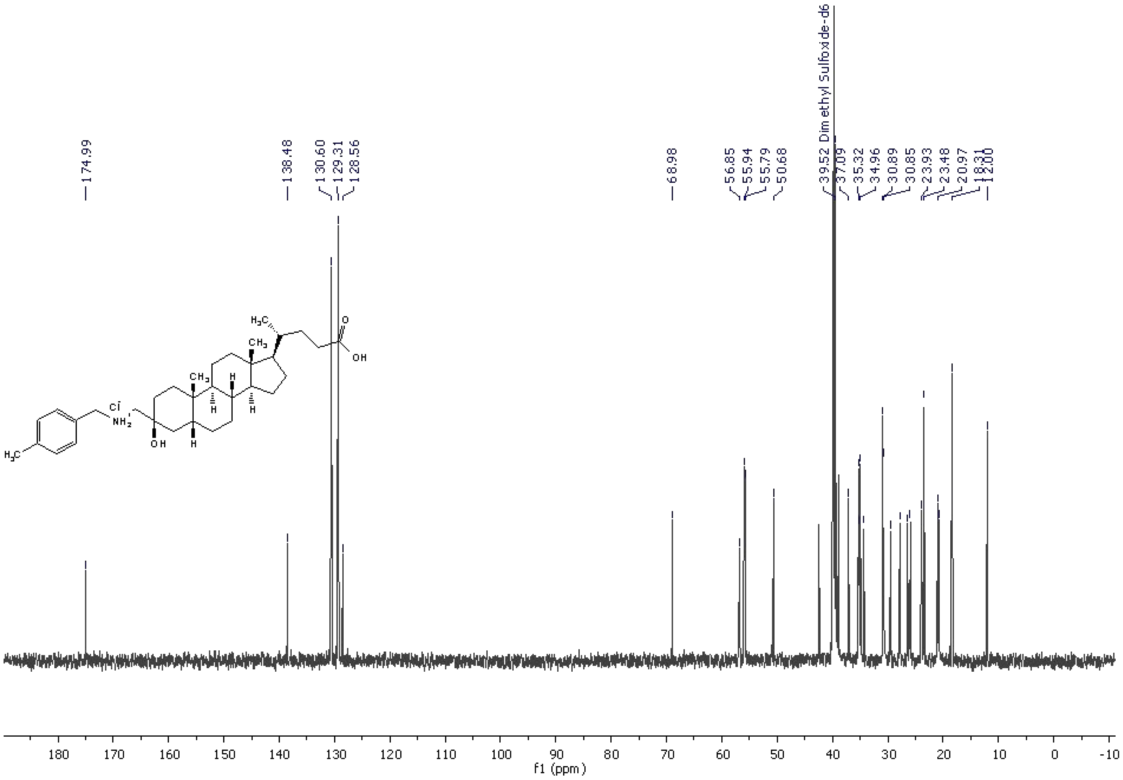

Figure S29. ^{13}C NMR (500 MHz) spectrum of compound **6f** in DMSO-d6

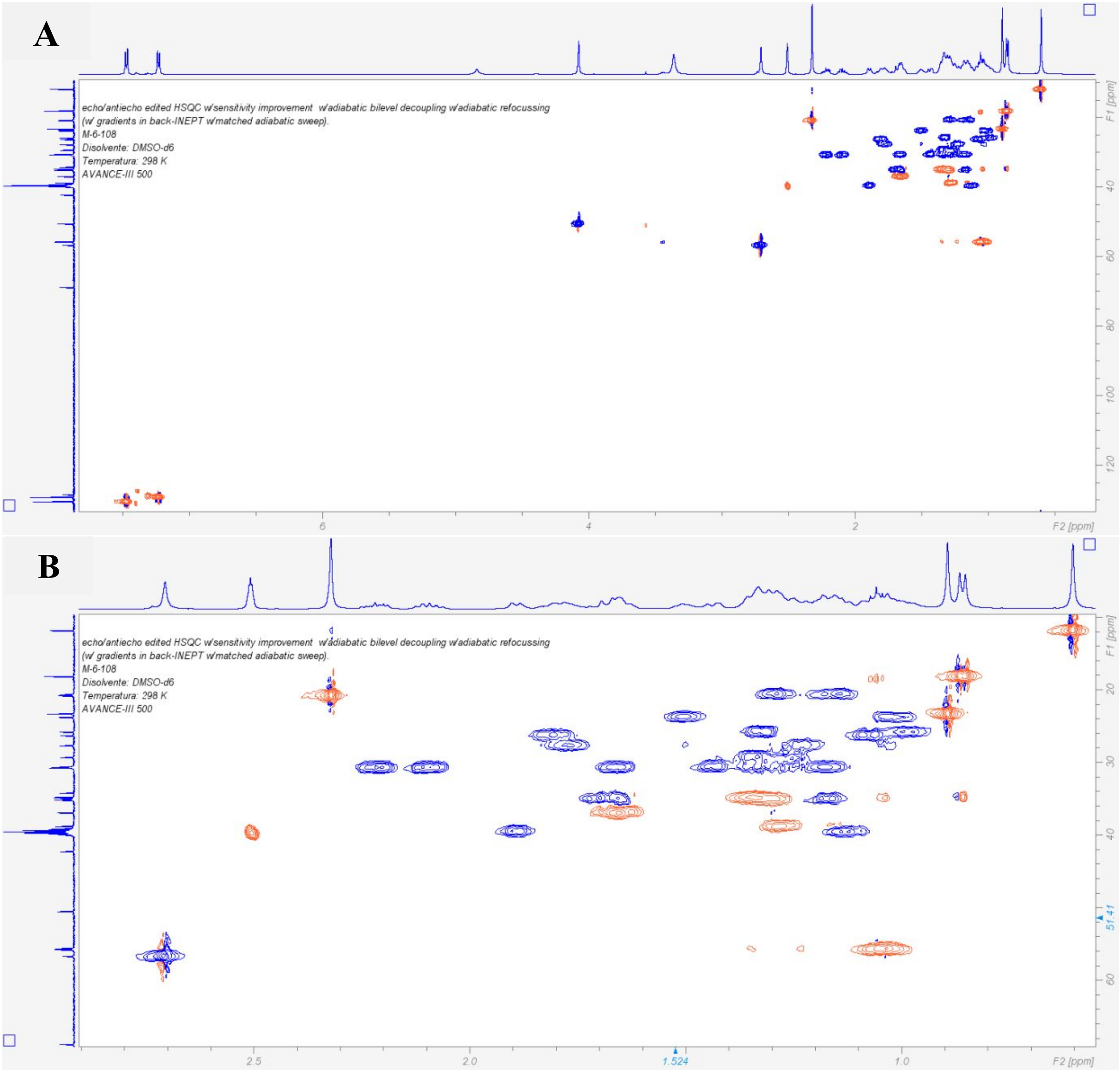

Figure S30. HSQC spectra of 6f. **A**. Full spectra. **B**. Highfield spectra.

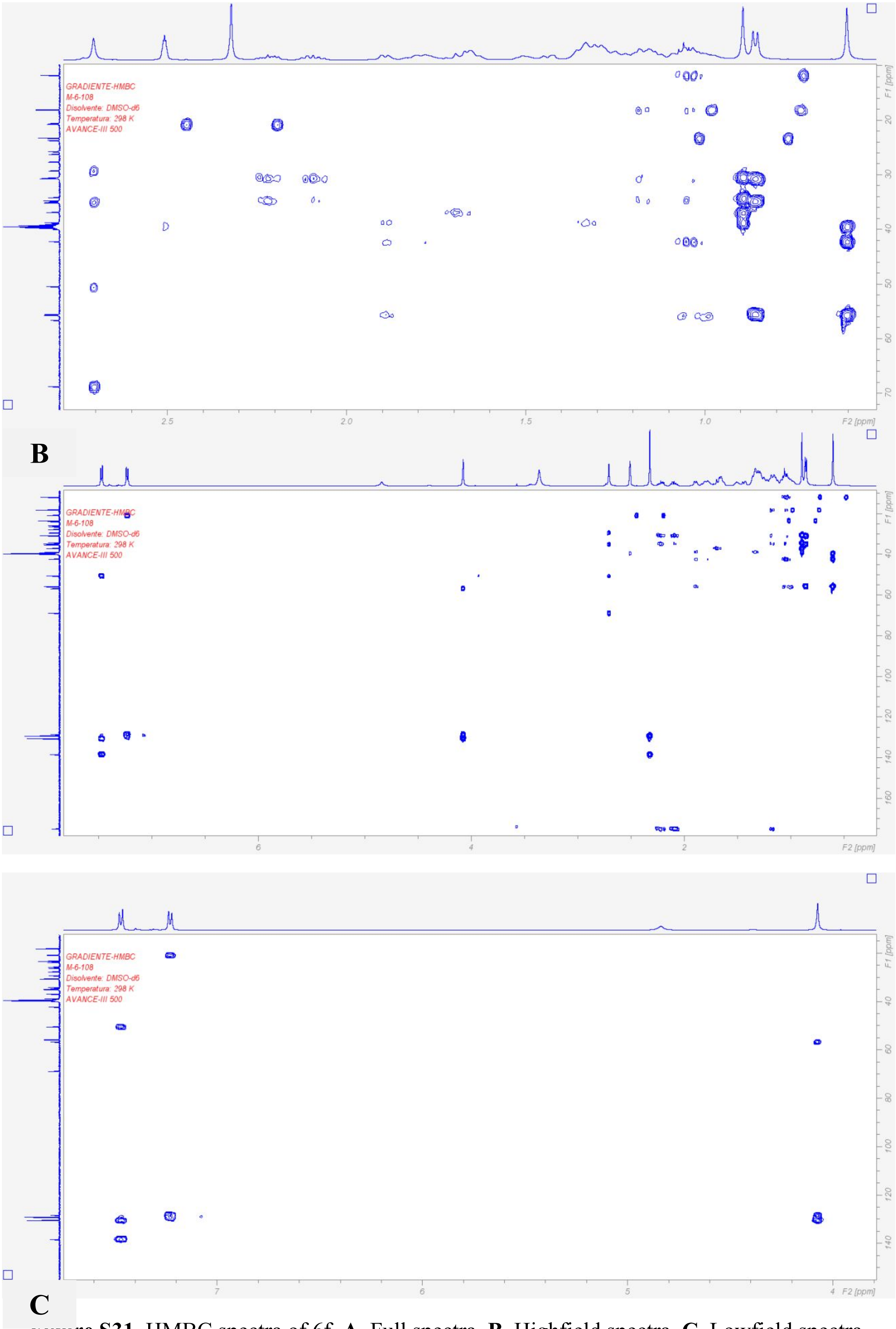

Figure S31. HMBC spectra of 6f. **A**. Full spectra. **B**. Highfield spectra. **C**. Lowfield spectra.

.

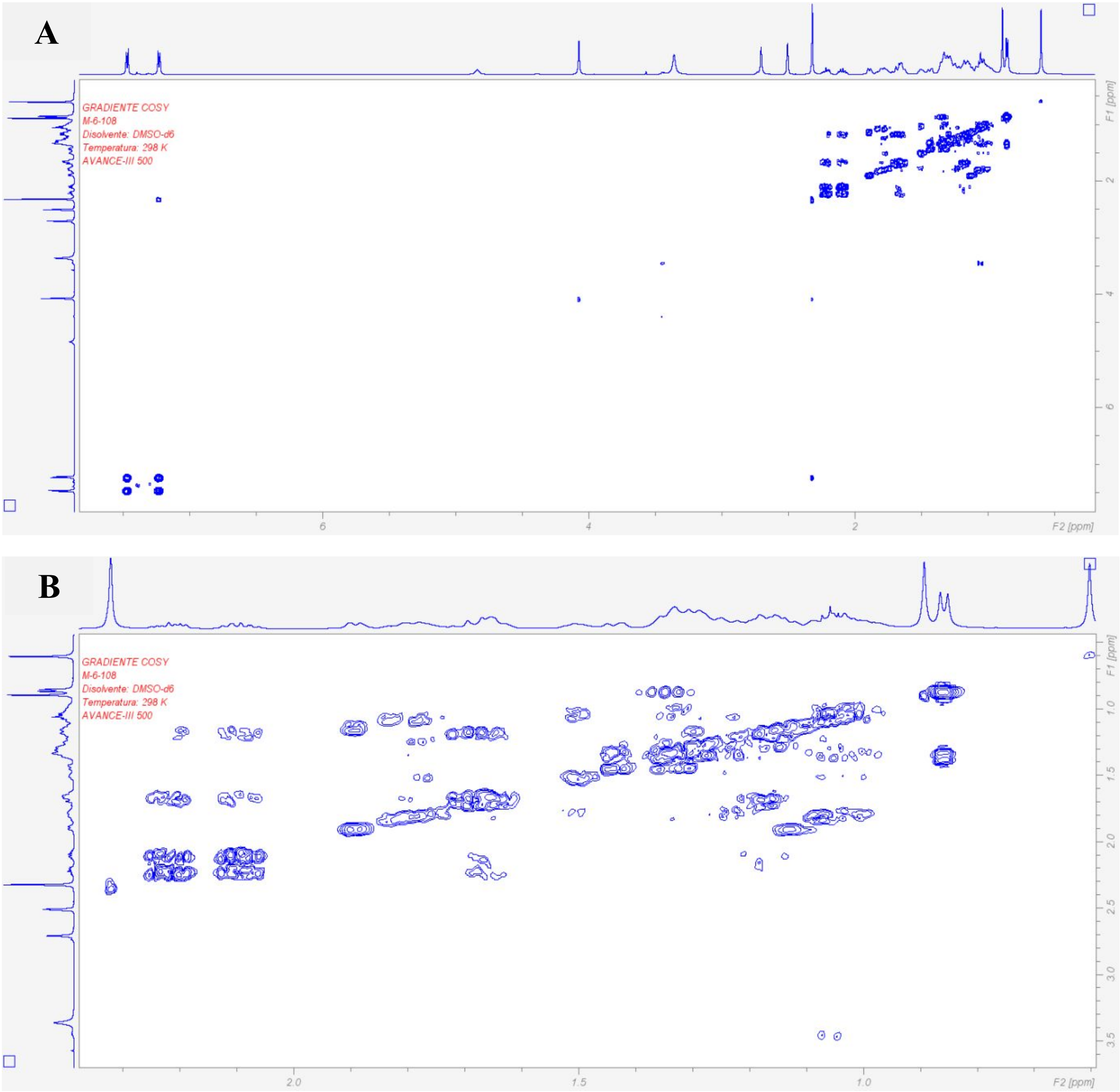

Figure S32. COSY spectra of 6f. **A**. Full spectra. **B**. Highfield spectra.

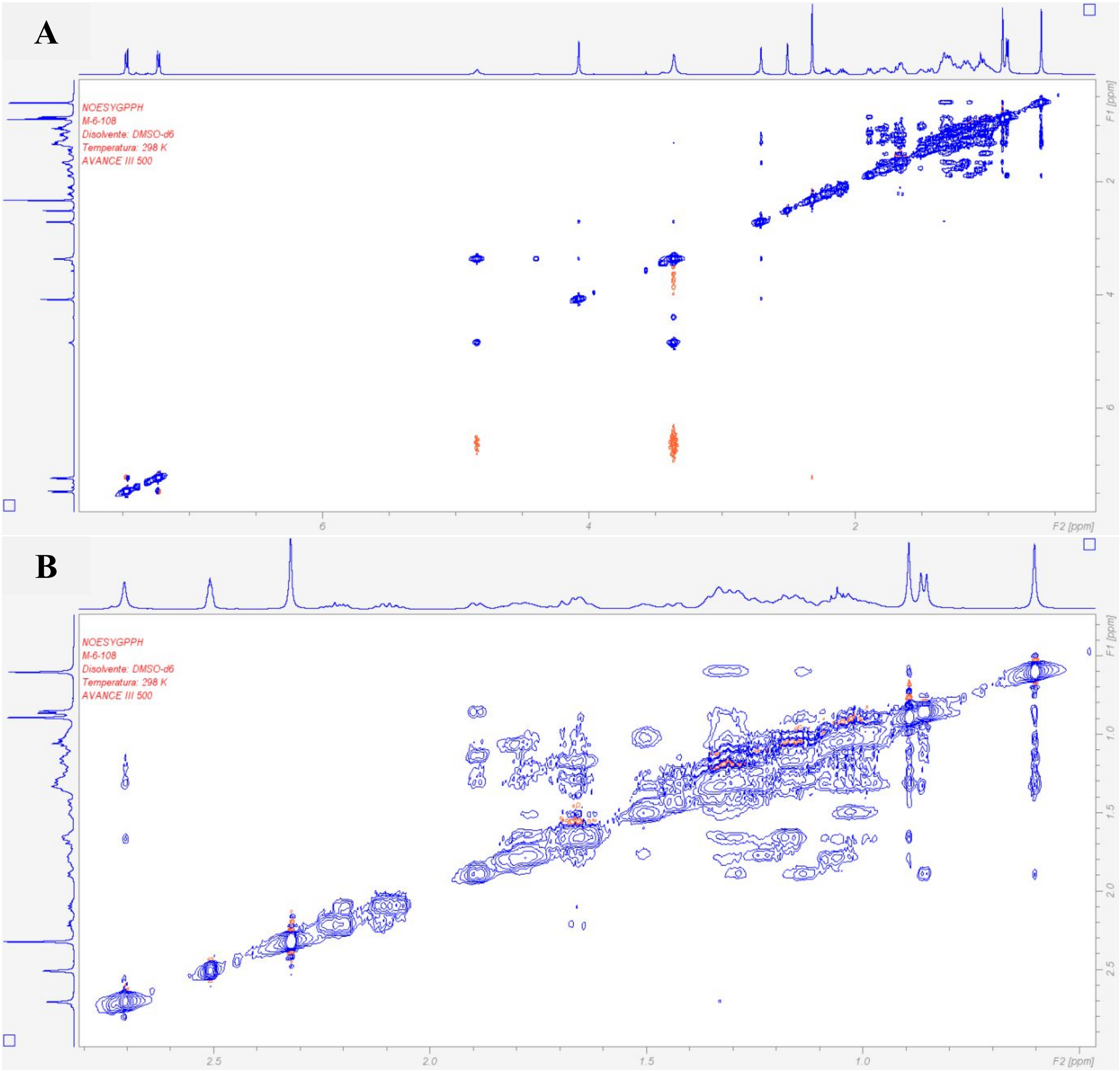

Figure S33. NOESY spectra of 6f. **A**. Full spectra. **B**. Highfield spectra.

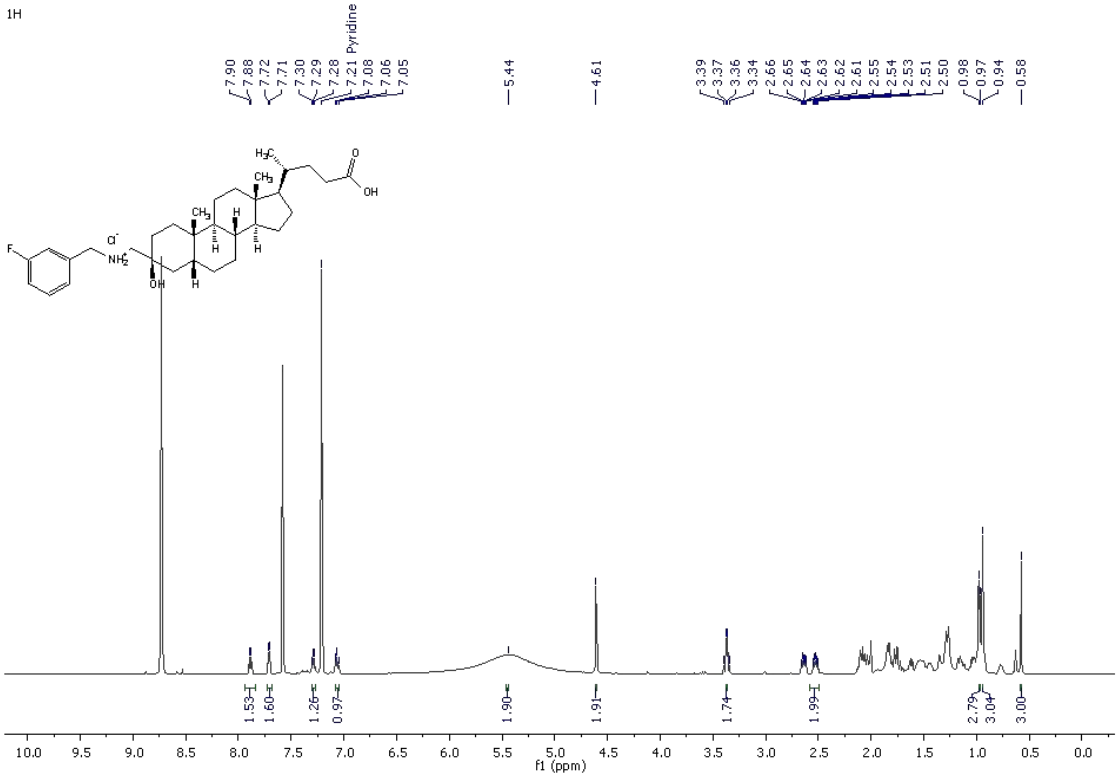

Figure S34. ^{1}H NMR (600 MHz) spectrum of compound **6g** in Pyridine-d5

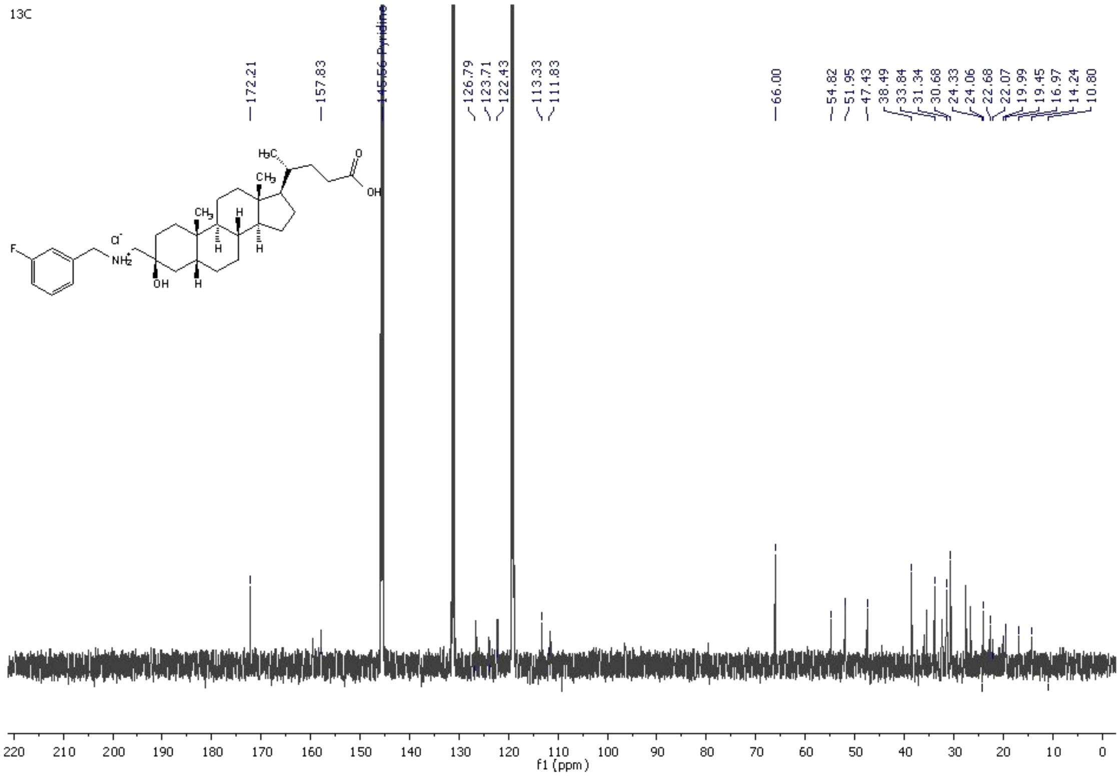

Figure S35. ^{13}C NMR (600 MHz) spectrum of compound **6g** in Pyridine-d5

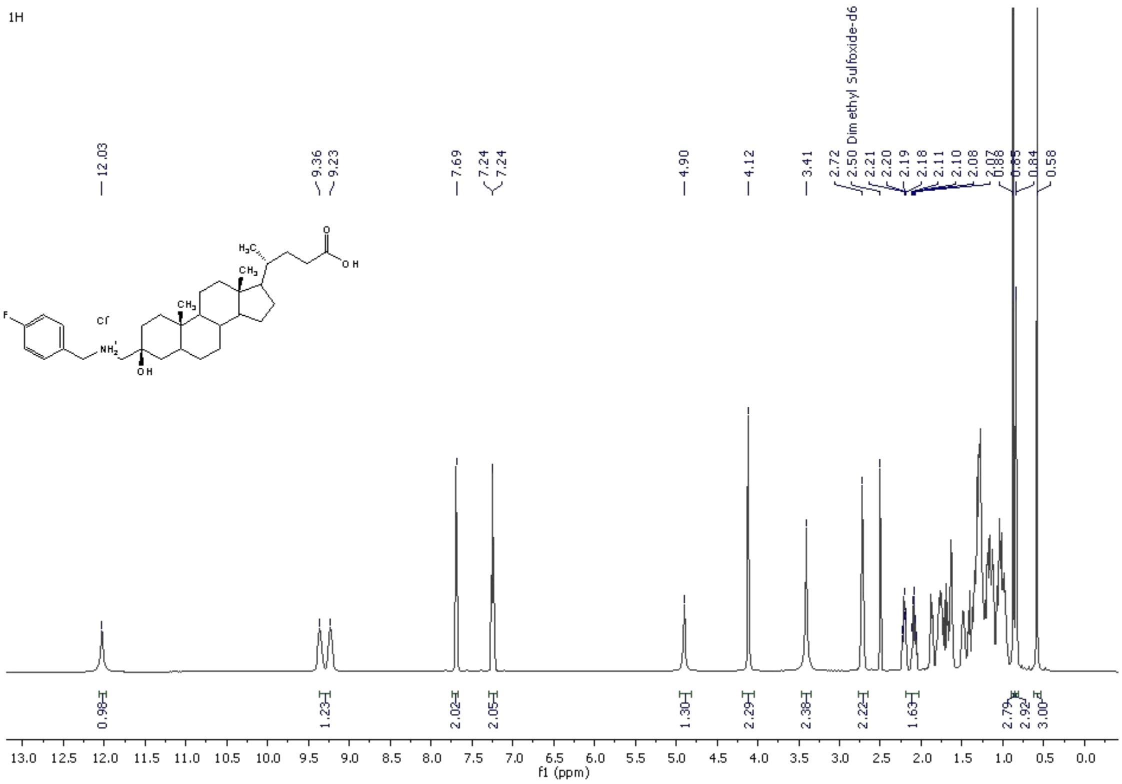

Figure S36. ^{1}H NMR (600 MHz) spectrum of compound **6h** in DMSO-d6

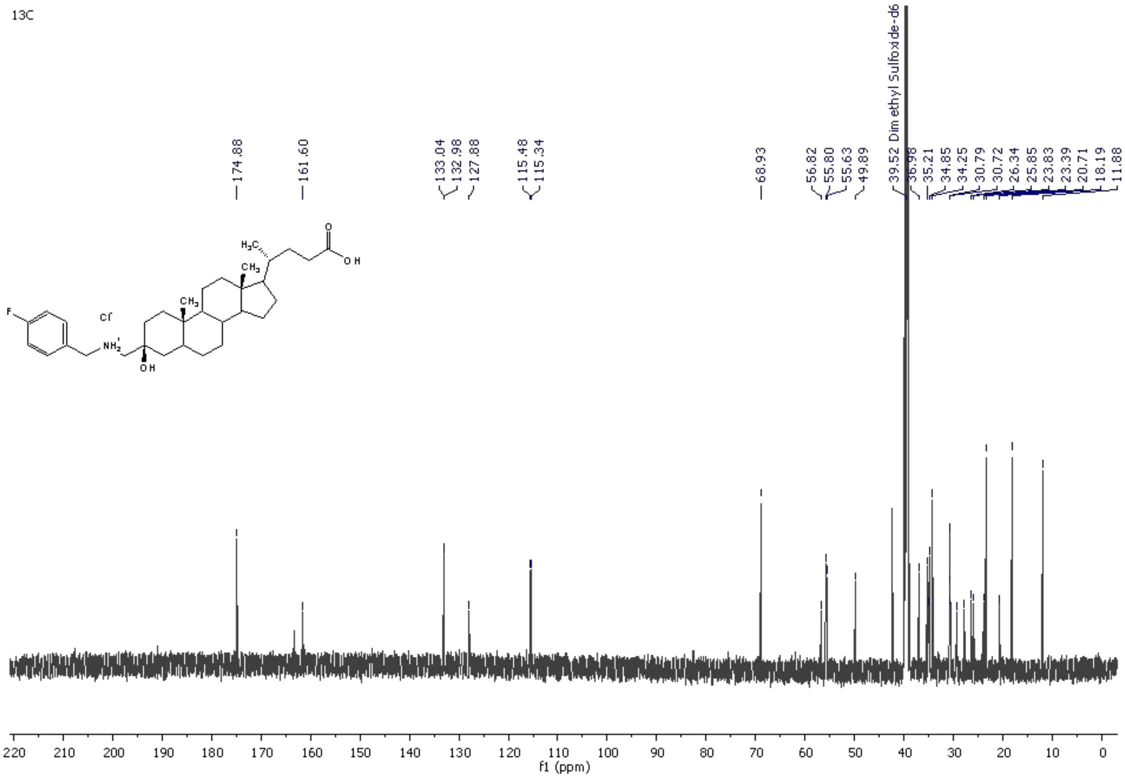

Figure S37. ^{13}C NMR (600 MHz) spectrum of compound **6h** in DMSO-d6

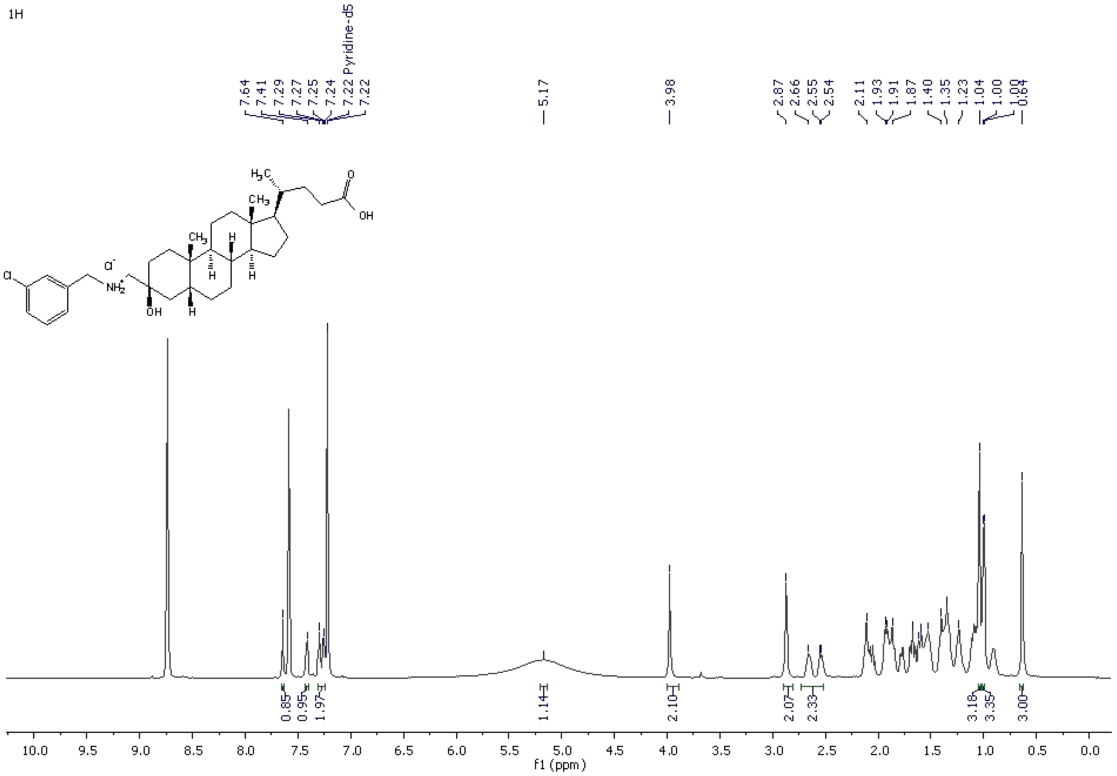

Figure S38. ^{1}H NMR (600 MHz) spectrum of compound **6i** in Pyridine-d5

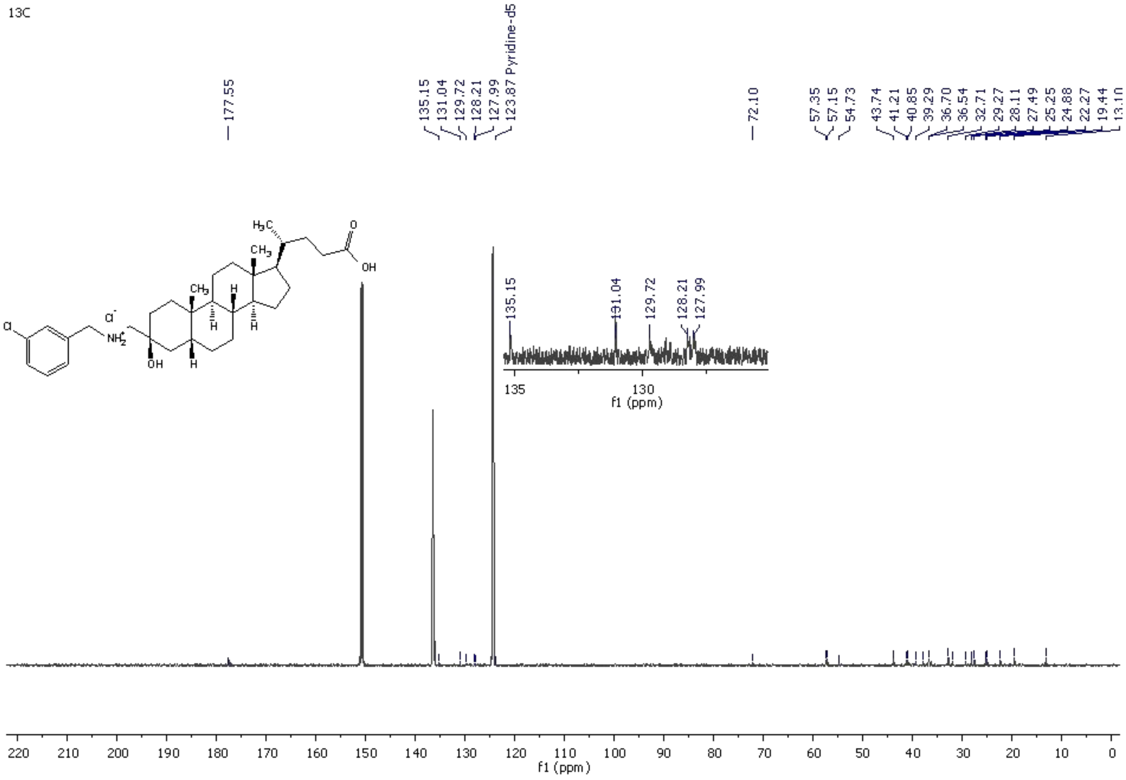

Figure S39. ^{13}C NMR (600 MHz) spectrum of compound **6i** in Pyridine-d5

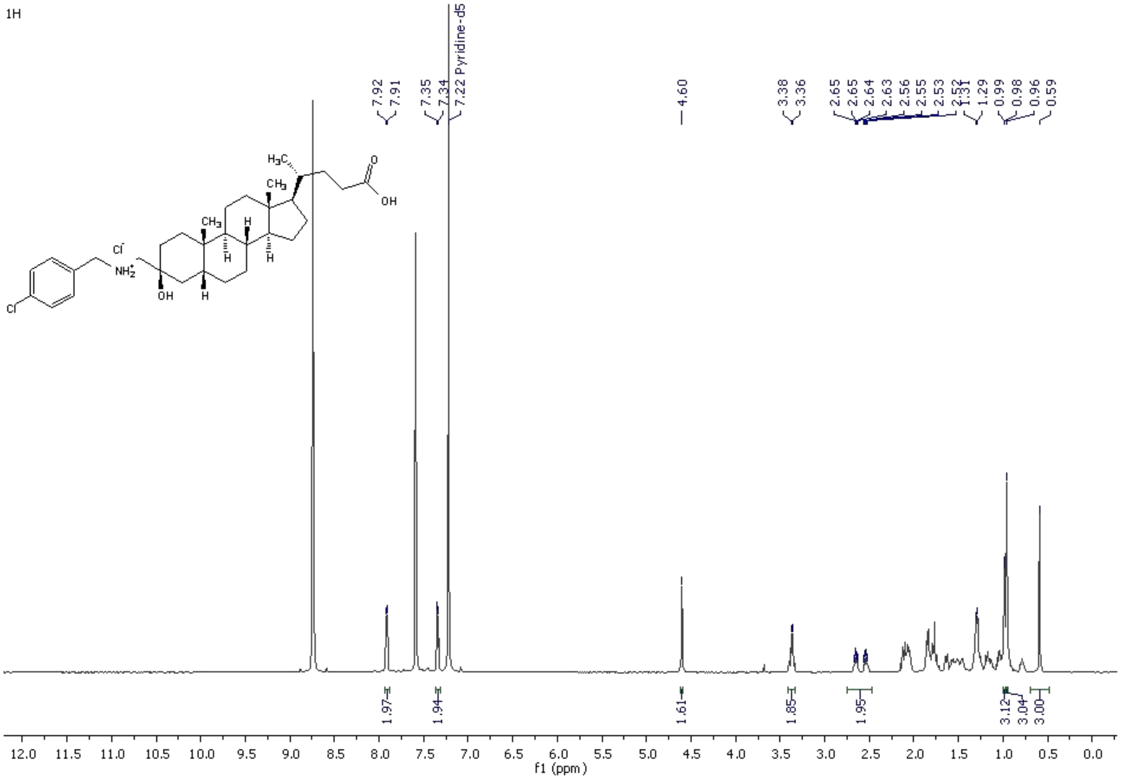

Figure S40. ^{1}H NMR (600 MHz) spectrum of compound **6j** in Pyridine-d5

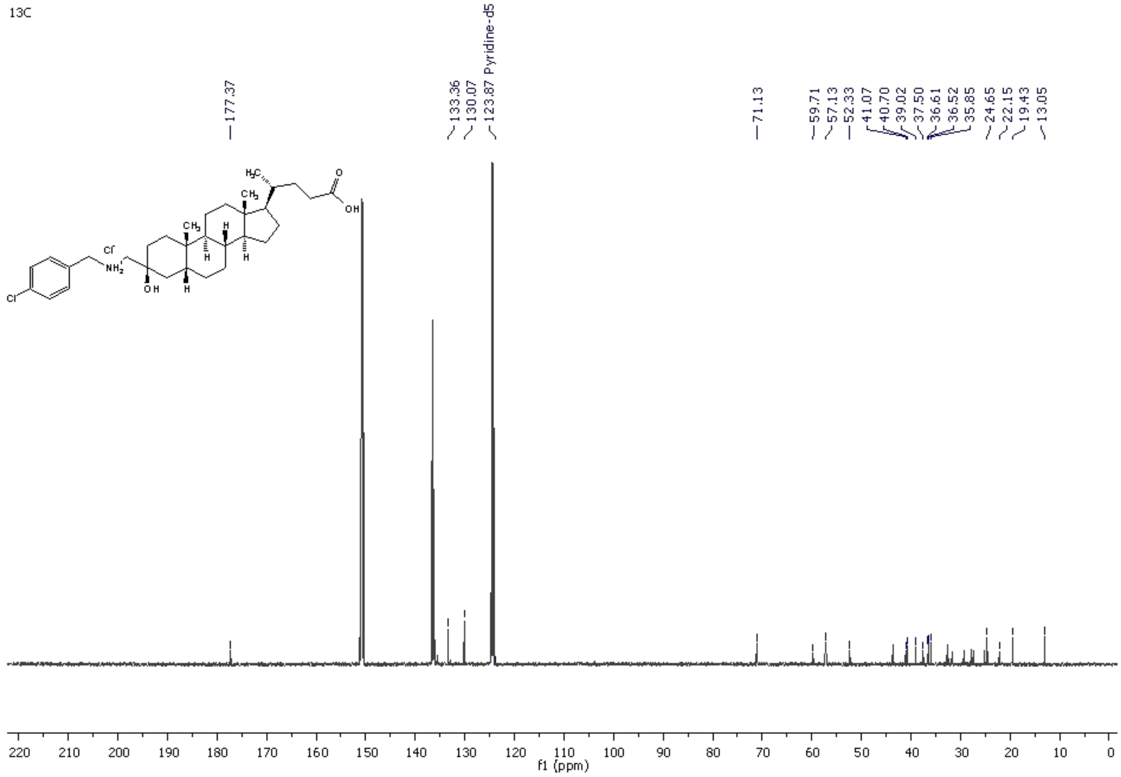

Figure S41. ^{13}C NMR (600 MHz) spectrum of compound **6j** in Pyridine-d5

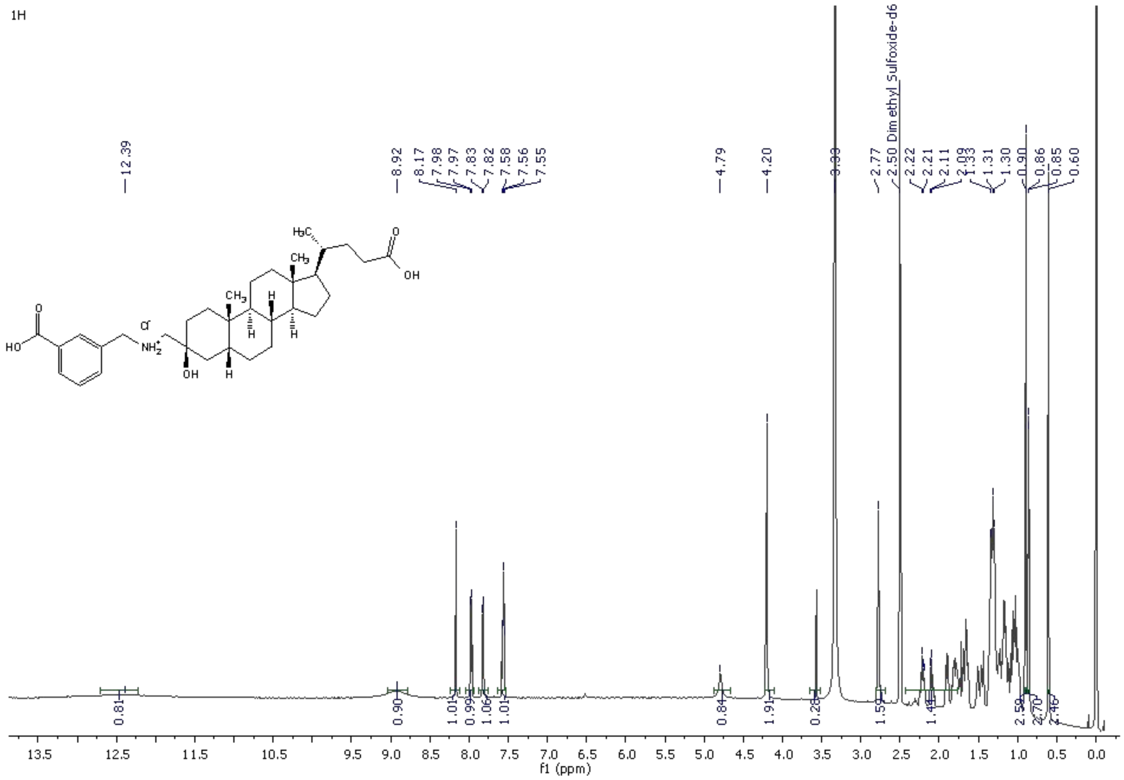

Figure S42. ^{1}H NMR (600 MHz) spectrum of compound **6k** in DMSO-d6

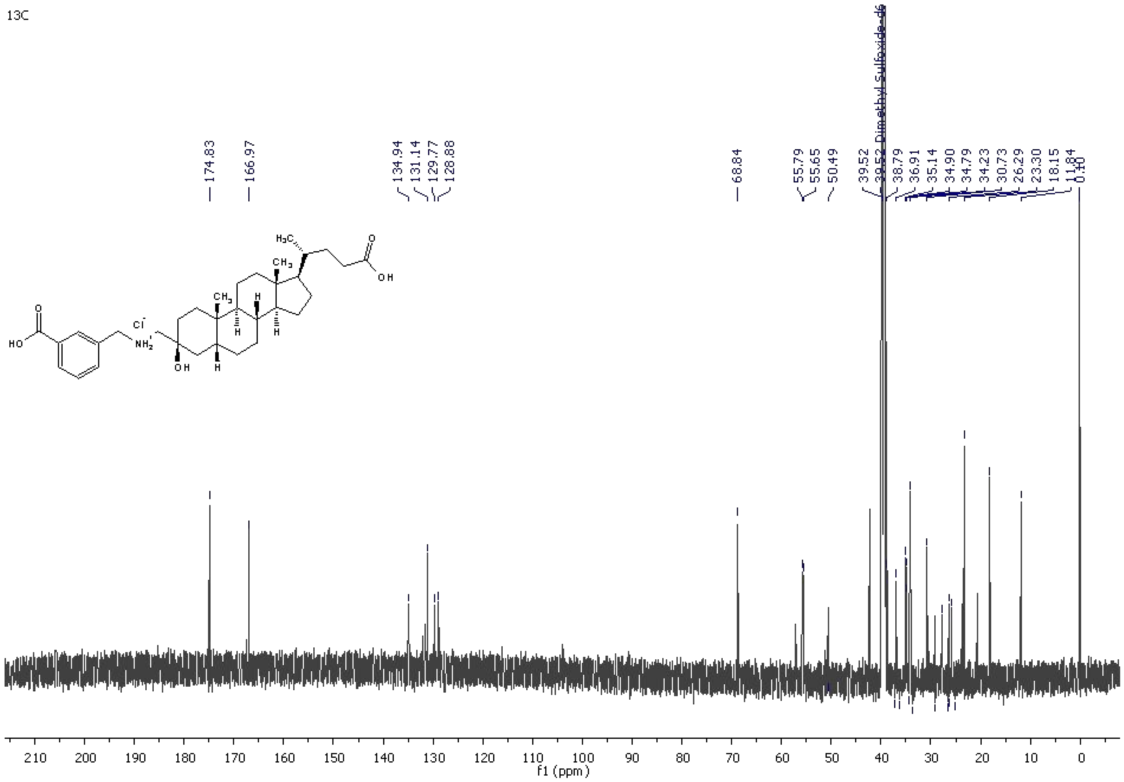

Figure S43. ^{13}C NMR (600 MHz) spectrum of compound **6k** in DMSO-d6

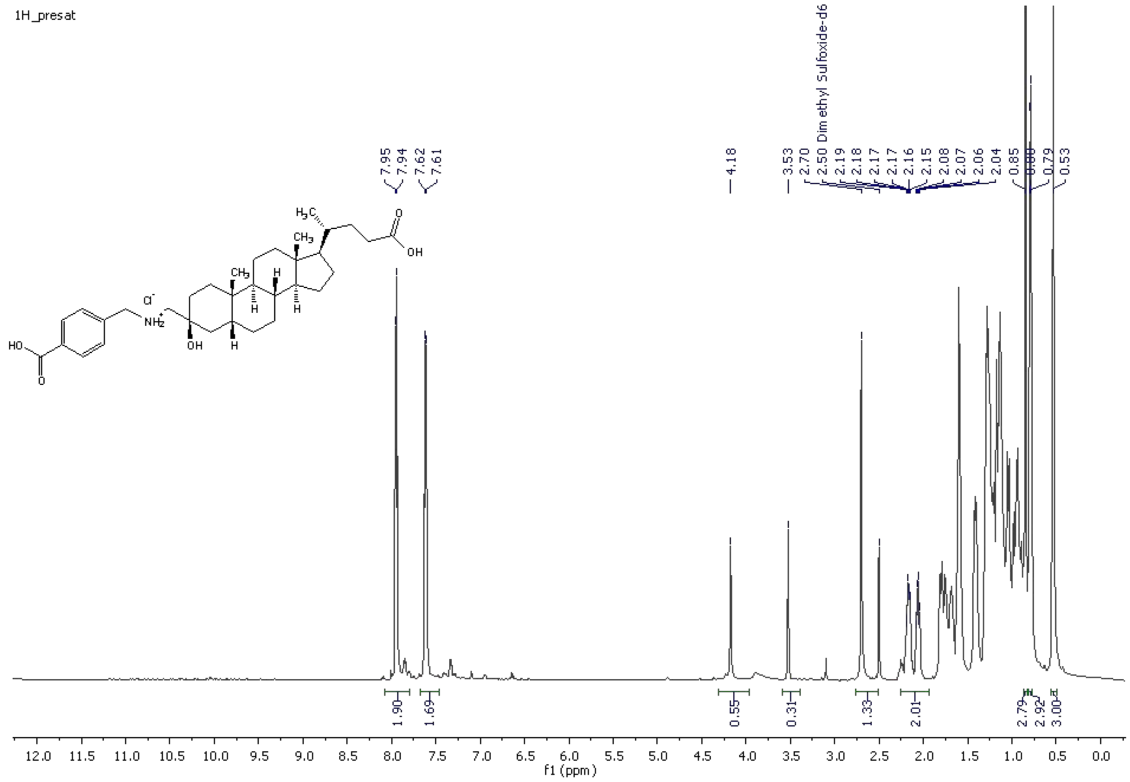

Figure S44. ^{1}H NMR (600 MHz) spectrum of compound **6l** in DMSO-d6

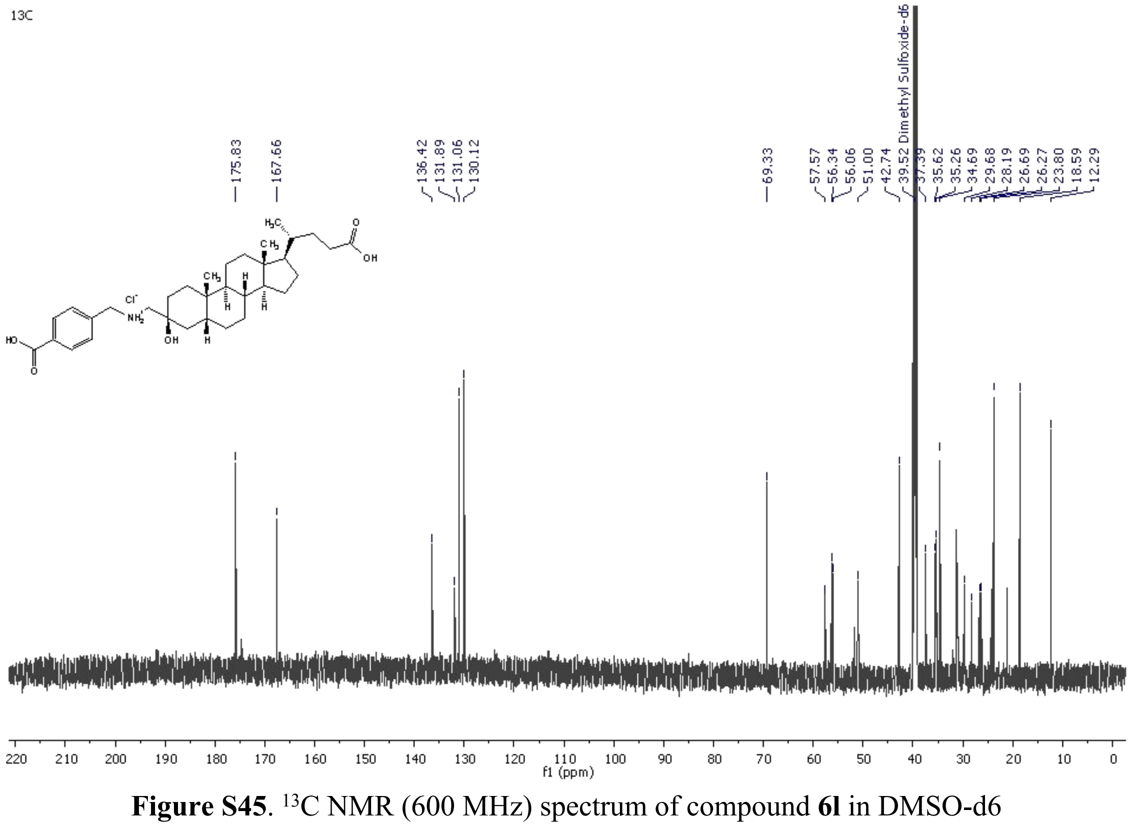

Figure S45. ^{13}C NMR (600 MHz) spectrum of compound **6l** in DMSO-d6

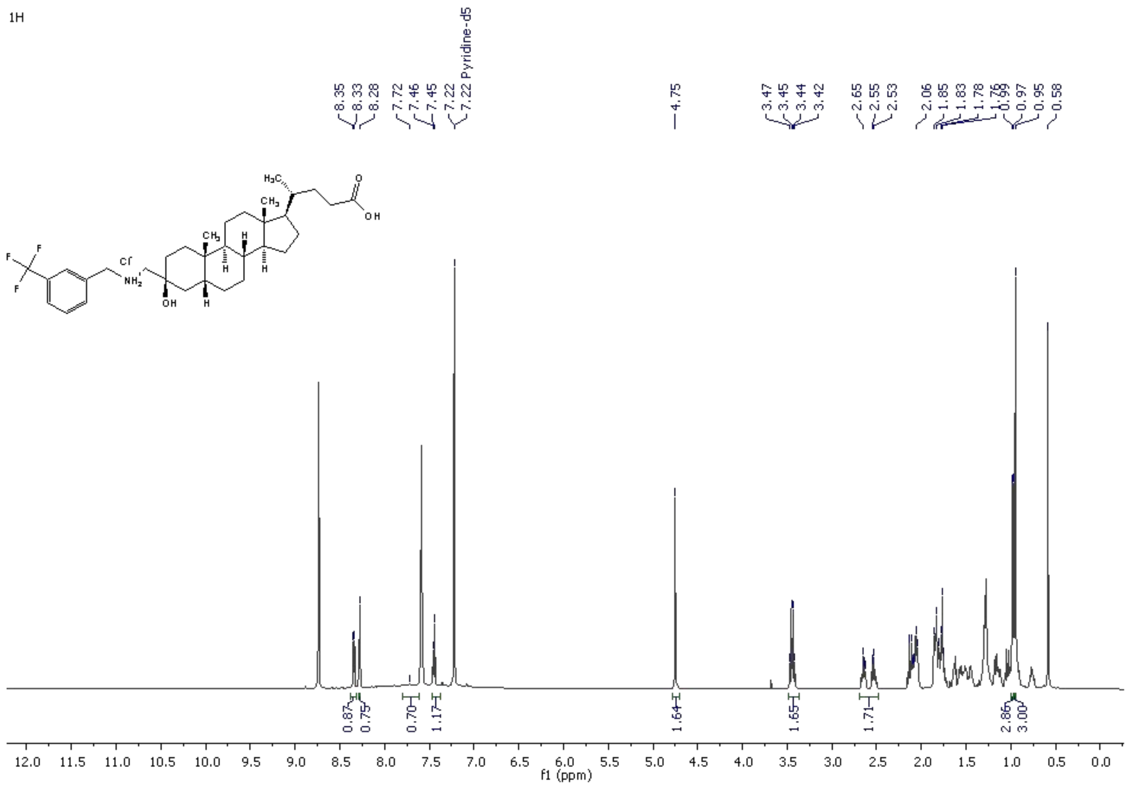

Figure S46. ^{1}H NMR (600 MHz) spectrum of compound **6m** in Pyridine-d5

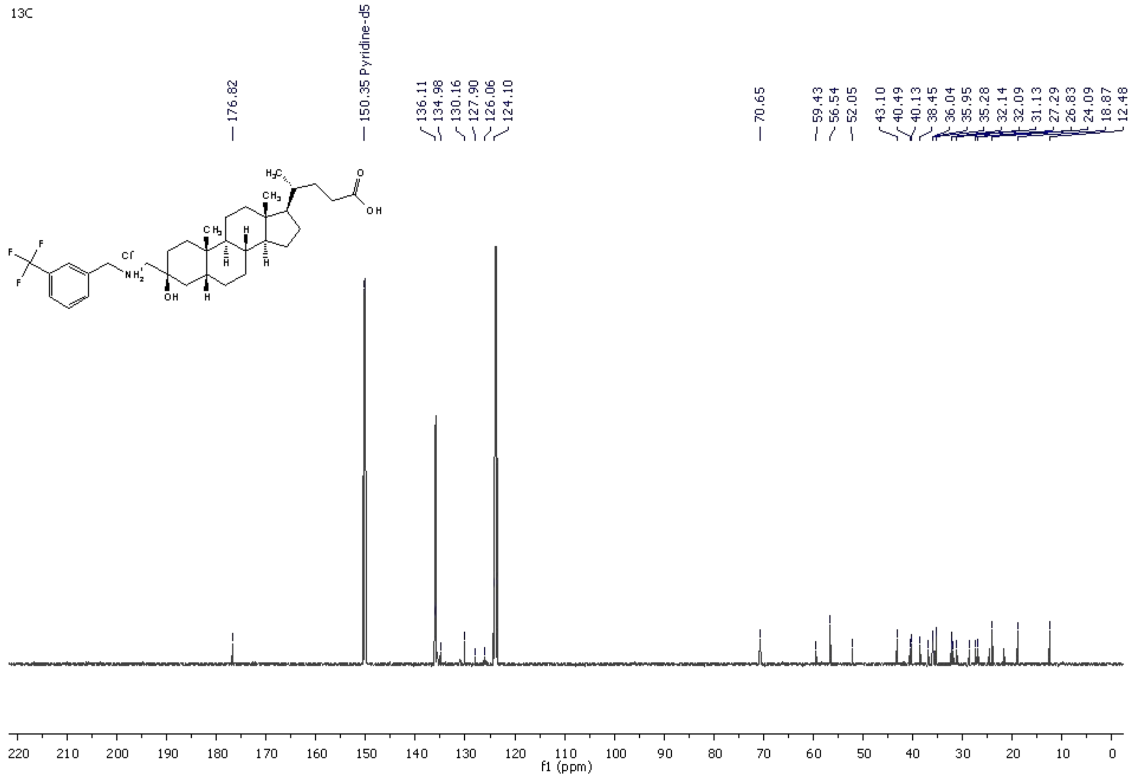

Figure S47. ^{13}C NMR (600 MHz) spectrum of compound **6m** in Pyridine-d5

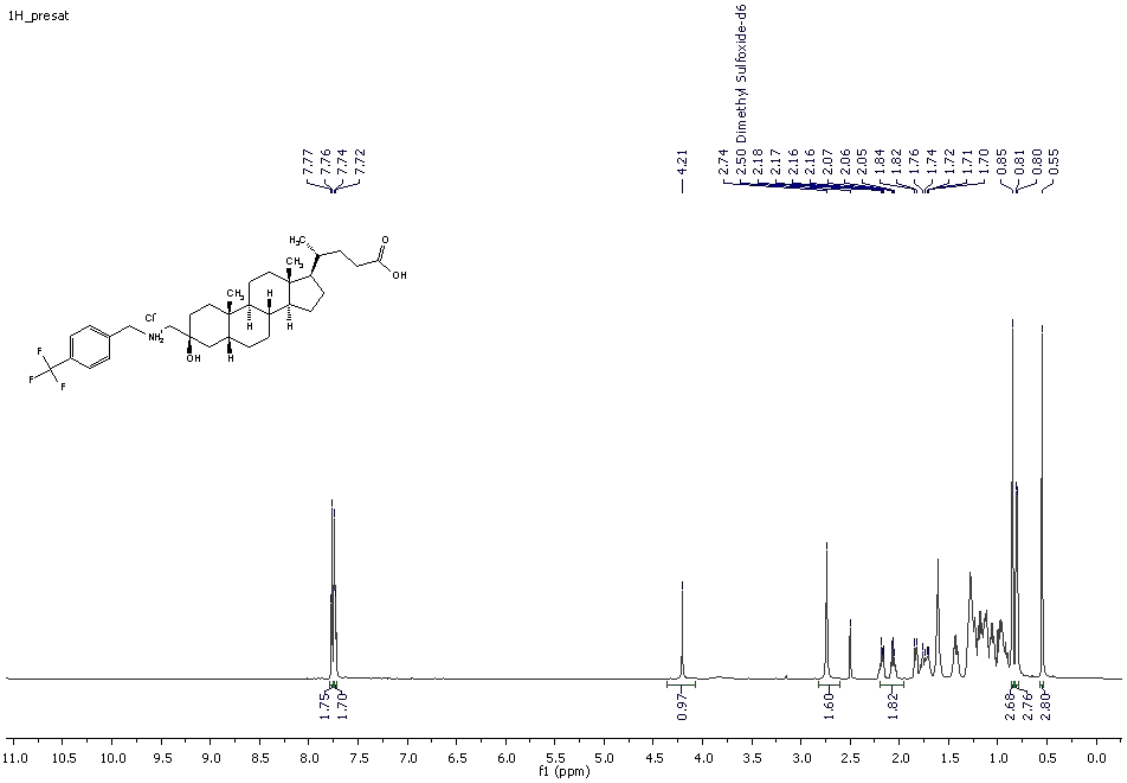

Figure S48. ^{1}H NMR (600 MHz) spectrum of compound **6n** in DMSO-d6

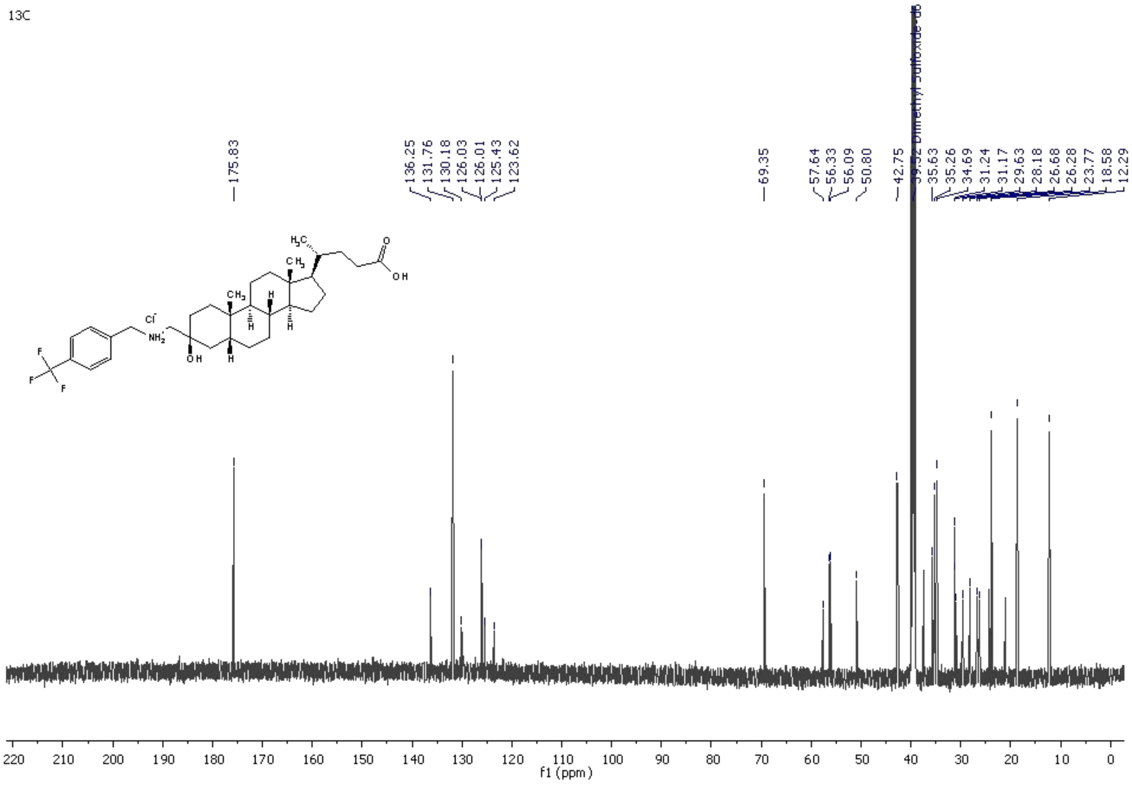

Figure S49. ^{13}C NMR (600 MHz) spectrum of compound **6n** in DMSO-d6

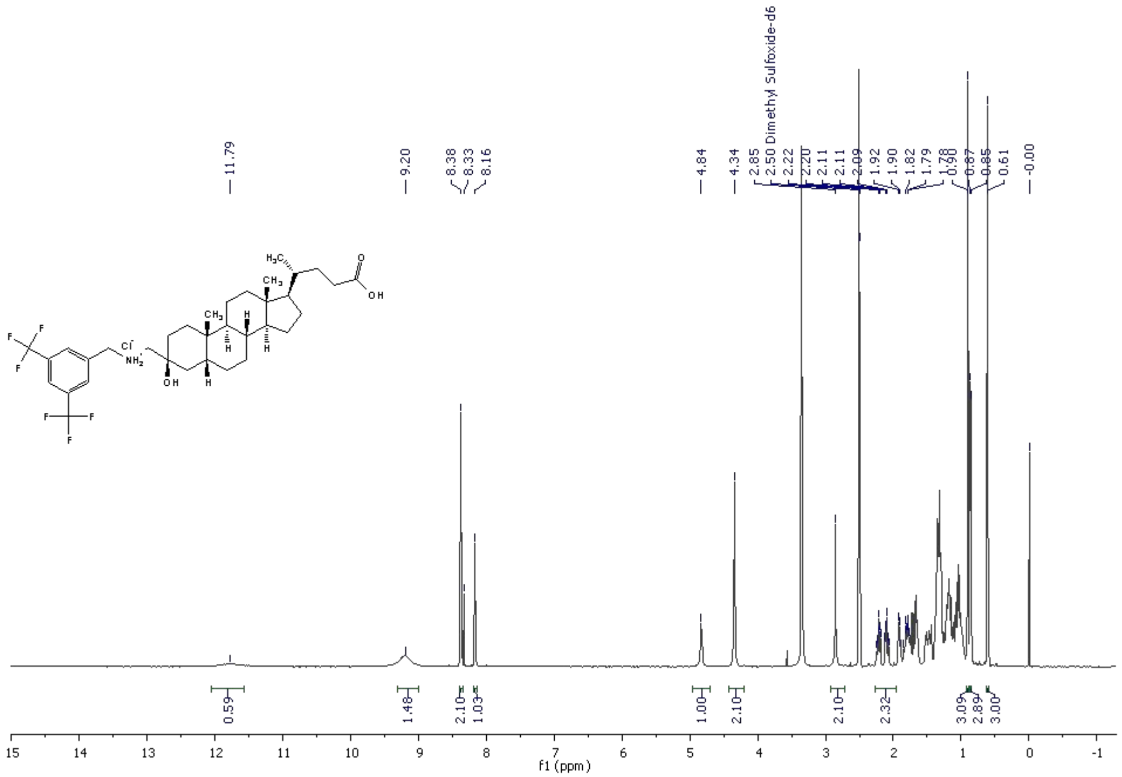

Figure S50. ^{1}H NMR (500 MHz) spectrum of compound **6o** in DMSO-d6

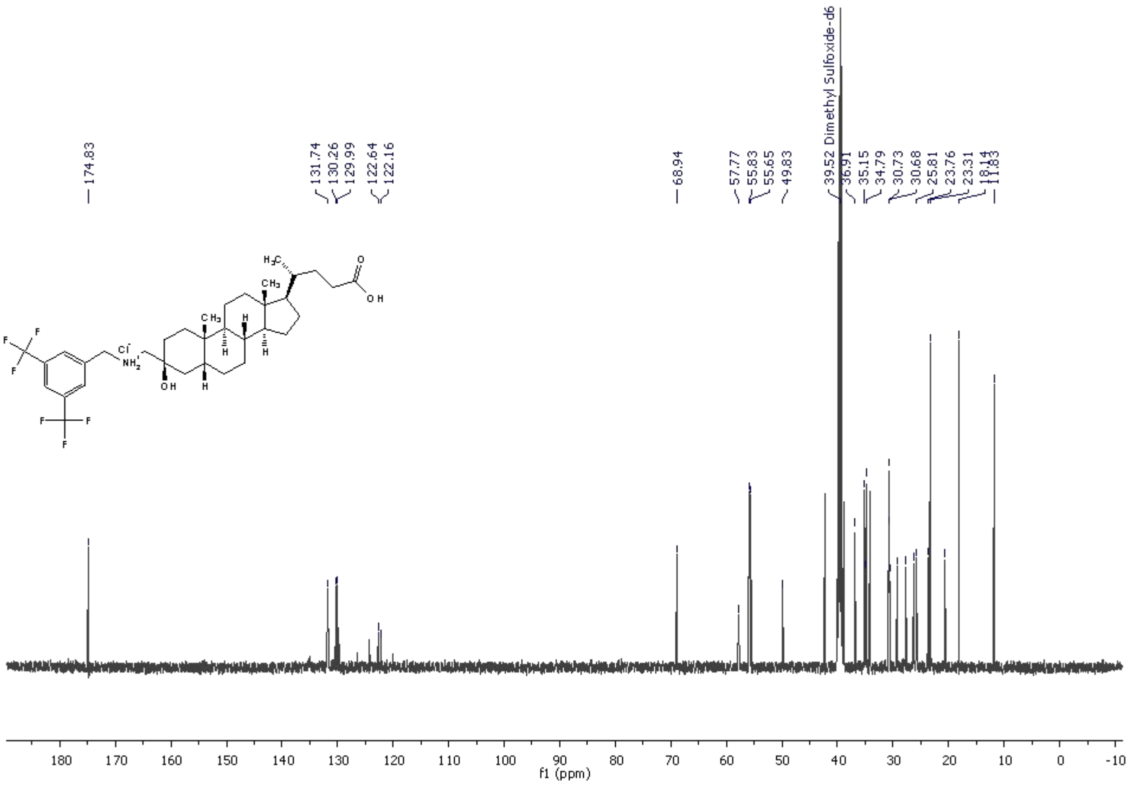

Figure S51. ^{13}C NMR (500 MHz) spectrum of compound **6o** in DMSO-d6

Mass Spectrum SmartFormula Report

Analysis Info

Analysis Name	D:\Data\Monica Rincon\Francisco Cortes Benitez\20230911_4.d	Acquisition Date	9/11/2023 5:20:35 PM
Method	Tune Low extendido.m	Operator	Admin
Sample Name	20230911_4	Instrument	micrOTOF 213750.00410
Comment			

Acquisition Parameter

Source Type	ESI	Ion Polarity	Positive	Set Nebulizer	0.5 Bar
Focus	Active			Set Dry Heater	150 °C
Scan Begin	50 m/z	Set Capillary	4500 V	Set Dry Gas	4.0 l/min
Scan End	3000 m/z	Set End Plate Offset	-500 V	Set Divert Valve	Waste

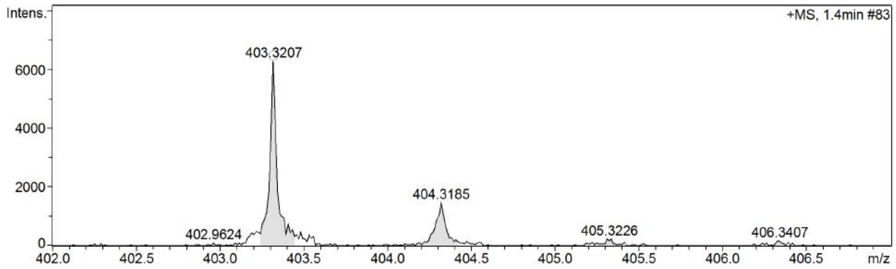

Meas. m/z	#	Ion Formula	Sum Formula	m/z	err [ppm]	|err| [mDa]	mSigma	# mSigma
403.3207	1	C26H43O3	C26H42O3	403.3207	-0.2	0.1	30.8	1
425.3025	1	C26H42NaO3		425.3026	0.3	0.1	28.4	1

Figure S52. Mass spectrum formula report of compound 4

Mass Spectrum SmartFormula Report

Analysis Info

Analysis Name	D:\Data\Monica Rincon\Francisco Cortes Benitez\20231006_6a.d	Acquisition Date	10/6/2023 5:04:00 PM
Method	Tune Low extendido.m	Operator	Admin
Sample Name	20231006_6a	Instrument	micrOTOF 213750.00410
Comment			

Acquisition Parameter

Source Type	ESI	Ion Polarity	Positive	Set Nebulizer	0.5 Bar
Focus	Active			Set Dry Heater	150 °C
Scan Begin	50 m/z	Set Capillary	4500 V	Set Dry Gas	4.0 l/min
Scan End	3000 m/z	Set End Plate Offset	-500 V	Set Divert Valve	Waste

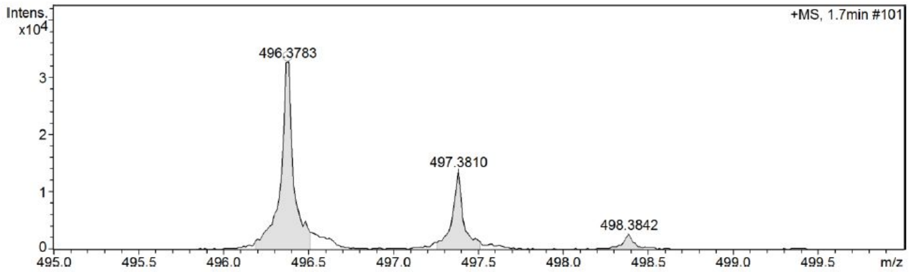

Meas. m/z	#	Ion Formula	Sum Formula	m/z	err [ppm]	|err| [mDa]	mSigma	# mSigma
496.3783	1	C32H50NO3	C32H50NO3	496.3785	0.5	0.3	32.5	1
	1	C32H50NO3	C32H49NO3	496.3785	0.5	0.3	32.5	1

Figure S53. Mass spectrum formula report of compound 6a

Mass Spectrum SmartFormula Report

Analysis Info

Analysis Name	D:\Data\Monica Rincon\Francisco Cortes Benitez\20231006_6b.d	Acquisition Date	10/6/2023 4:36:35 PM
Method	Tune Low extendido.m	Operator	Admin
Sample Name	20231006_6b	Instrument	micrOTOF 213750.00410
Comment			

Acquisition Parameter

Source Type	ESI	Ion Polarity	Positive	Set Nebulizer	0.5 Bar
Focus	Active			Set Dry Heater	150 °C
Scan Begin	50 m/z	Set Capillary	4500 V	Set Dry Gas	4.0 l/min
Scan End	3000 m/z	Set End Plate Offset	-500 V	Set Divert Valve	Waste

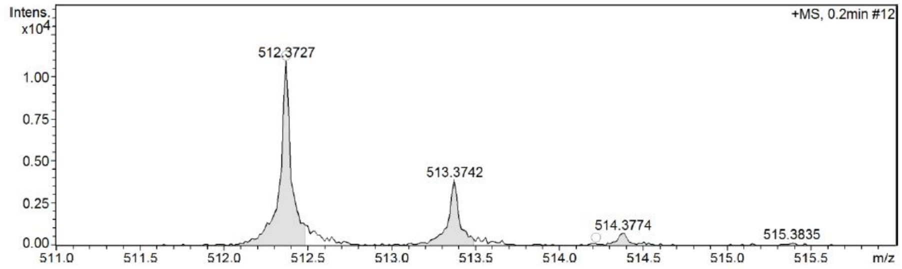

Meas. m/z	#	Ion Formula	Sum Formula	err [ppm]	|err| [mDa]	m/z	mSigma	# mSigma
512.3727	1	C32H50NO4	C32H50NO4	1.4	0.7	512.3734	4.0	1
	1	C32H50NO4	C32H49NO4	1.4	0.7	512.3734	4.0	1

Figure S54. Mass spectrum formula report of compound 6b

Mass Spectrum SmartFormula Report

Analysis Info

Analysis Name	D:\Data\Monica Rincon\Francisco Cortes Benitez\20231005_6c.d	Acquisition Date	10/5/2023 4:37:32 PM
Method	Tune Low extendido.m	Operator	Admin
Sample Name	20231005_6c	Instrument	micrOTOF 213750.00410
Comment			

Acquisition Parameter

Source Type	ESI	Ion Polarity	Positive	Set Nebulizer	0.5 Bar
Focus	Active			Set Dry Heater	150 °C
Scan Begin	50 m/z	Set Capillary	4500 V	Set Dry Gas	4.0 l/min
Scan End	3000 m/z	Set End Plate Offset	-500 V	Set Divert Valve	Waste

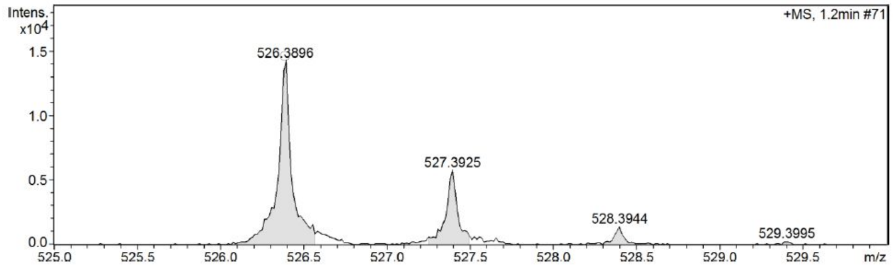

Meas. m/z	#	Ion Formula	m/z	err [ppm]	mSigma	# mSigma	Score	rdb	e⁻ Conf	N-Rule
526.3896	1	C33H52NO4	526.3891	-1.0	21.0	1	100.00	9.0	even	ok

Figure S55. Mass spectrum formula report of compound 6c

Mass Spectrum SmartFormula Report

Analysis Info

Analysis Name	D:\Data\Monica Rincon\Francisco Cortes Benitez\20231005_6d.d	Acquisition Date	10/5/2023 4:01:47 PM
Method	Tune Low extendido.m	Operator	Admin
Sample Name	20231005_6d	Instrument	micrOTOF 213750.00410
Comment			

Acquisition Parameter

Source Type	ESI	Ion Polarity	Positive	Set Nebulizer	0.5 Bar
Focus	Active			Set Dry Heater	150 °C
Scan Begin	50 m/z	Set Capillary	4500 V	Set Dry Gas	4.0 l/min
Scan End	3000 m/z	Set End Plate Offset	-500 V	Set Divert Valve	Waste

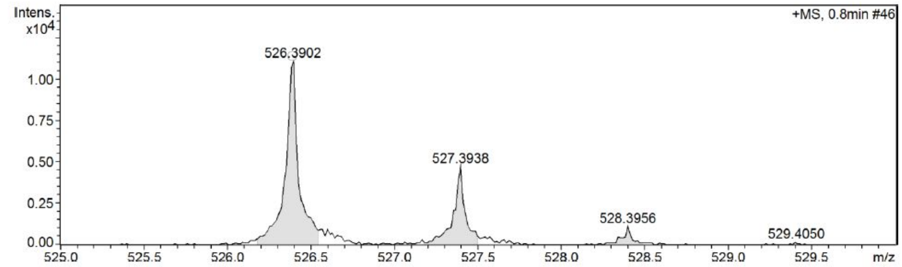

Meas. m/z	#	Ion Formula	Sum Formula	m/z	err [ppm]	err [mDa]	mSigma	# mSigma
526.3902	1	C33H52NO4	C33H51NO4	526.3891	-2.2	-1.2	31.9	2

Figure S56. Mass spectrum formula report of compound 6d

Mass Spectrum SmartFormula Report

Analysis Info

Analysis Name	D:\Data\Monica Rincon\Francisco Cortes Benitez\20240322_6e.d	Acquisition Date	3/22/2024 12:15:56 PM
Method	Tune Low extendido.m	Operator	Admin
Sample Name	20240322_6e	Instrument	micrOTOF 213750.00410
Comment			

Acquisition Parameter

Source Type	ESI	Ion Polarity	Positive	Set Nebulizer	0.5 Bar
Focus	Active			Set Dry Heater	150 °C
Scan Begin	50 m/z	Set Capillary	4500 V	Set Dry Gas	4.0 l/min
Scan End	3000 m/z	Set End Plate Offset	-500 V	Set Divert Valve	Waste

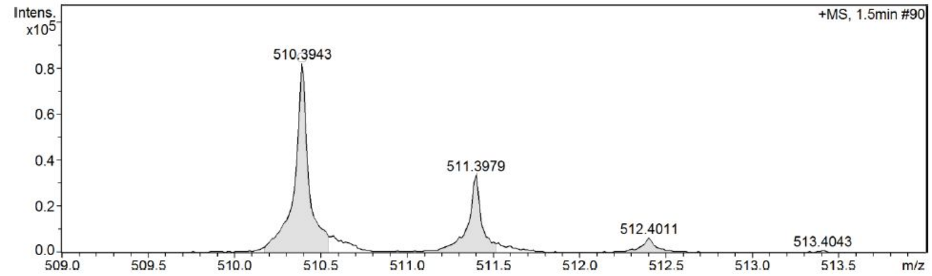

Meas. m/z	#	Ion Formula	Sum Formula	m/z	err [ppm]	|err| [mDa]	mSigma	# mSigma
510.3943	1	C33H52NO3	C33H52NO3	510.3942	-0.3	0.2	21.3	1

Figure S57. Mass spectrum formula report of compound 6e

Mass Spectrum SmartFormula Report

Analysis Info

Analysis Name	D:\Data\Monica Rincon\Francisco Cortes Benitez\20240322_6f.d	Acquisition Date	3/22/2024 11:59:25 AM
Method	Tune Low extendido.m	Operator	Admin
Sample Name	20240322_6f	Instrument	micrOTOF 213750.00410
Comment			

Acquisition Parameter

Source Type	ESI	Ion Polarity	Positive	Set Nebulizer	0.5 Bar
Focus	Active			Set Dry Heater	150 °C
Scan Begin	50 m/z	Set Capillary	4500 V	Set Dry Gas	4.0 l/min
Scan End	3000 m/z	Set End Plate Offset	-500 V	Set Divert Valve	Waste

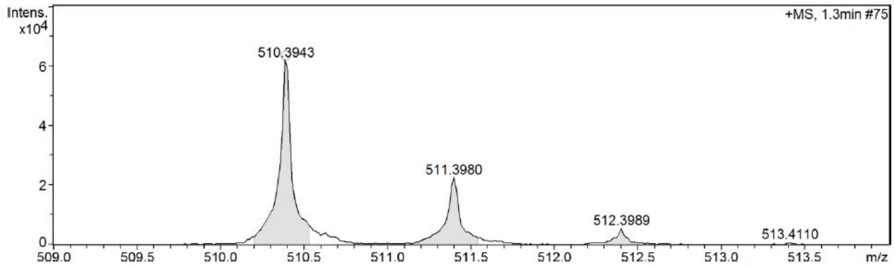

Meas. m/z	#	Ion Formula	Sum Formula	m/z	err [ppm]	|err| [mDa]	mSigma	# mSigma
510.3943	1	C33H52NO3	C33H52NO3	510.3942	-0.2	0.1	7.9	1

Figure S58. Mass spectrum formula report of compound 6f

Mass Spectrum SmartFormula Report

Analysis Info

Analysis Name	D:\Data\Monica Rincon\Francisco Cortes Benitez\20240322_6g_.d	Acquisition Date	3/22/2024 1:16:12 PM
Method	Tune Low extendido.m	Operator	Admin
Sample Name	20240322_6g_	Instrument	micrOTOF 213750.00410
Comment			

Acquisition Parameter

Source Type	ESI	Ion Polarity	Positive	Set Nebulizer	0.5 Bar
Focus	Active			Set Dry Heater	150 °C
Scan Begin	50 m/z	Set Capillary	4500 V	Set Dry Gas	4.0 l/min
Scan End	3000 m/z	Set End Plate Offset	-500 V	Set Divert Valve	Waste

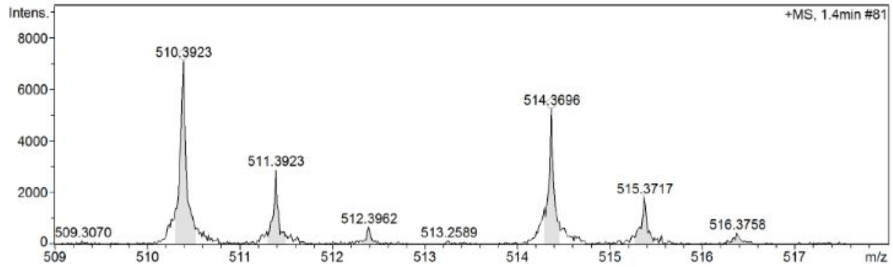

Meas. m/z	#	Ion Formula	Sum Formula	m/z	err [ppm]	|err| [mDa]	mSigma	# mSigma
510.3923	1	C33H52NO3	C33H52NO3	510.3942	3.7	1.9	24.4	1
514.3696	1	C32H49FNO3	C32H49FNO3	514.3691	-1.0	0.5	9.6	1

Figure S59. Mass spectrum formula report of compound 6g

Mass Spectrum SmartFormula Report

**Analysis Info**		Acquisition Date	3/22/2024 1:16:12 PM
Analysis Name	D:\Data\Monica Rincon\Francisco Cortes Benitez\20240322_6h_.d		
Method	Tune Low extendido.m	Operator	Admin
Sample Name	20240322_6h_	Instrument	micrOTOF 213750.00410
Comment			

Acquisition Parameter

Source Type	ESI	Ion Polarity	Positive	Set Nebulizer	0.5 Bar
Focus	Active			Set Dry Heater	150 °C
Scan Begin	50 m/z	Set Capillary	4500 V	Set Dry Gas	4.0 l/min
Scan End	3000 m/z	Set End Plate Offset	-500 V	Set Divert Valve	Waste

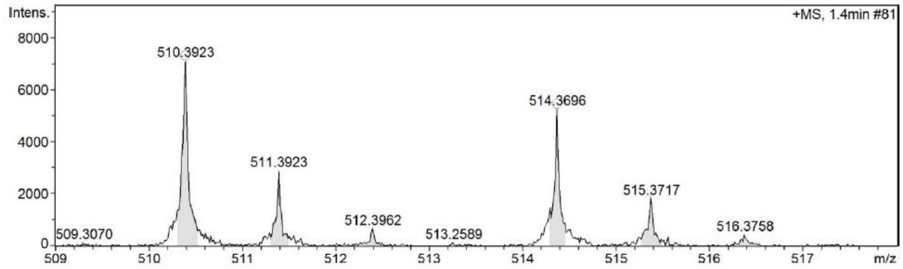

Meas. m/z	#	Ion Formula	Sum Formula	m/z	err [ppm]	|err| [mDa]	mSigma	# mSigma
510.3923	1	C33H52NO3	C33H52NO3	510.3942	3.7	1.9	24.4	1
514.3696	1	C32H49FNO3	C32H49FNO3	514.3691	-1.0	0.5	9.6	1

Figure S60. Mass spectrum formula report of compound 6h

Mass Spectrum SmartFormula Report

**Analysis Info**		Acquisition Date	10/6/2023 5:22:02 PM
Analysis Name	D:\Data\Monica Rincon\Francisco Cortes Benitez\20231006_6i.d		
Method	Tune Low extendido.m	Operator	Admin
Sample Name	20231006_6i	Instrument	micrOTOF 213750.00410
Comment			

Acquisition Parameter

Source Type	ESI	Ion Polarity	Positive	Set Nebulizer	0.5 Bar
Focus	Active			Set Dry Heater	150 °C
Scan Begin	50 m/z	Set Capillary	4500 V	Set Dry Gas	4.0 l/min
Scan End	3000 m/z	Set End Plate Offset	-500 V	Set Divert Valve	Waste

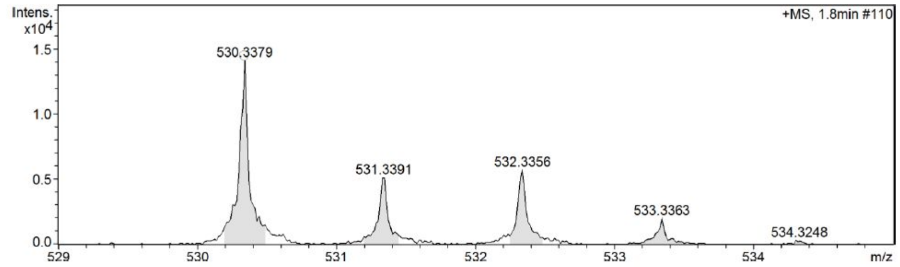

Meas. m/z	#	Ion Formula	Sum Formula	m/z	err [ppm]	|err| [mDa]	mSigma	# mSigma
530.3379	1	C32H49ClNO3	C32H49ClNO3	530.3395	3.1	1.6	15.1	1
	1	C32H49ClNO3	C32H48ClNO3	530.3395	3.1	1.6	15.1	1

Figure S61. Mass spectrum formula report of compound 6i

Mass Spectrum SmartFormula Report

Analysis Info

Analysis Name	D:\Data\Monica Rincon\Francisco Cortes Benitez\20231006_6j.d	Acquisition Date	10/6/2023 4:49:34 PM
Method	Tune Low extendido.m	Operator	Admin
Sample Name	20231006_6j	Instrument	micrOTOF 213750.00410
Comment			

Acquisition Parameter

Source Type	ESI	Ion Polarity	Positive	Set Nebulizer	0.5 Bar
Focus	Active			Set Dry Heater	150 °C
Scan Begin	50 m/z	Set Capillary	4500 V	Set Dry Gas	4.0 l/min
Scan End	3000 m/z	Set End Plate Offset	-500 V	Set Divert Valve	Waste

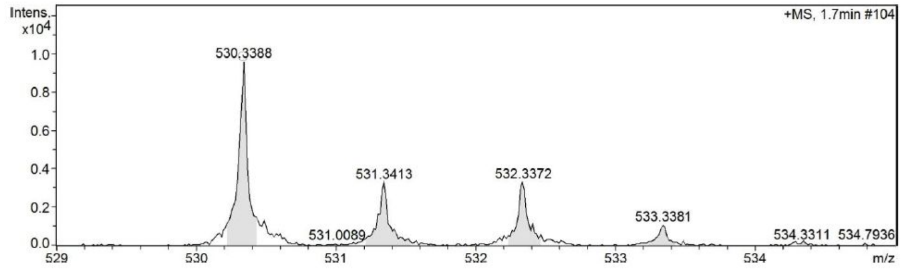

Meas. m/z	#	Ion Formula	Sum Formula	m/z	err [ppm]	|err| [mDa]	mSigma	# mSigma
530.3388	1	C32H49ClNO3	C32H49ClNO3	530.3395	1.4	0.7	22.7	2
	1	C32H49ClNO3	C32H48ClNO3	530.3395	1.4	0.7	22.7	2

Figure S62. Mass spectrum formula report of compound 6j

Mass Spectrum List Report

Analysis Info

Analysis Name	D:\Data\Monica Rincon\Francisco Cortes Benitez\20231006_6k.d	Acquisition Date	10/6/2023 5:41:26 PM
Method	Tune Low extendido.m	Operator	Admin
Sample Name	20231006_6k	Instrument	micrOTOF 213750.00410
Comment			

Acquisition Parameter

Source Type	ESI	Ion Polarity	Positive	Set Nebulizer	0.5 Bar
Focus	Active			Set Dry Heater	150 °C
Scan Begin	50 m/z	Set Capillary	4500 V	Set Dry Gas	4.0 l/min
Scan End	3000 m/z	Set End Plate Offset	-500 V	Set Divert Valve	Waste

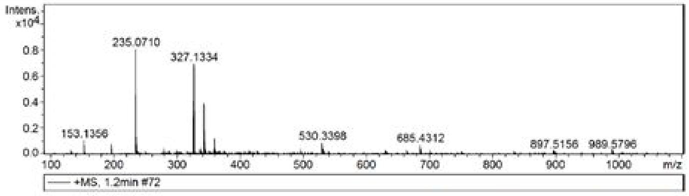

#	m/z	Res.	S/N	I	I %	FWHM
1	91.0507	7043	12.1	476	5.9	0.0129
2	131.9578	3306	7.1	278	3.4	0.0399
3	133.9524	4914	4.9	191	2.4	0.0273
4	153.1356	7219	28.6	1102	13.6	0.0212
5	197.0945	10180	20.6	804	10.0	0.0194
6	235.0710	7569	206.3	8074	100.0	0.0311
7	236.0748	8424	34.7	1356	16.8	0.0280
8	237.0813	10757	8.8	344	4.3	0.0220
9	239.1679	16200	4.9	192	2.4	0.0148
10	251.0424	13543	4.0	158	2.0	0.0185
11	281.1723	19331	6.9	272	3.4	0.0145
12	288.2764	18905	5.2	204	2.5	0.0152
13	301.1360	6890	6.5	256	3.2	0.0437
14	316.0468	26758	4.2	166	2.1	0.0118
15	327.1334	9043	177.2	6930	85.8	0.0362
16	328.1336	8369	39.5	1545	19.1	0.0392
17	329.1396	7591	6.4	250	3.1	0.0434
18	337.0980	7147	6.3	246	3.0	0.0472
19	338.3363	7862	6.8	264	3.3	0.0430
20	343.1082	9692	101.2	3946	48.9	0.0354
21	344.1101	10964	27.2	1060	13.1	0.0314
22	345.1077	5380	8.3	323	4.0	0.0641
23	351.1166	13139	8.1	315	3.9	0.0267
24	358.9743	5444	6.1	236	2.9	0.0659
25	360.3224	9645	32.2	1257	15.6	0.0374
26	360.9767	10308	4.7	184	2.3	0.0350
27	361.3268	9209	9.9	384	4.8	0.0392
28	368.4198	10168	8.9	348	4.3	0.0362
29	376.2975	6330	4.9	189	2.3	0.0595
30	408.3034	17618	4.7	180	2.2	0.0232
31	415.0408	8107	5.7	216	2.7	0.0512
32	417.0311	20517	5.3	203	2.5	0.0203
33	427.1495	7950	9.2	350	4.3	0.0537
34	496.3587	5363	7.2	268	3.3	0.0925
35	530.3398	9134	25.6	934	11.6	0.0581
36	531.3400	23694	14.3	520	6.4	0.0224
37	532.3318	7368	11.7	427	5.3	0.0722
38	533.3321	16908	5.4	198	2.5	0.0315
39	540.3516	21424	5.3	192	2.4	0.0252
40	631.2752	6422	6.0	211	2.6	0.0983
41	632.2792	27597	5.2	182	2.3	0.0229
42	664.4595	10810	10.9	378	4.7	0.0615
43	685.4312	9208	22.3	771	9.5	0.0744
44	686.4372	22991	14.8	511	6.3	0.0299
45	701.4134	8494	9.0	311	3.9	0.0826
46	834.4913	11753	5.4	178	2.2	0.0710
47	897.5156	4223	8.9	293	3.6	0.2125
48	898.5159	11612	7.8	255	3.2	0.0774
49	989.5796	7715	9.2	296	3.7	0.1283
50	990.5745	9193	6.9	220	2.7	0.1077

Figure S63. Mass spectrum formula report of compound 6k

Mass Spectrum SmartFormula Report

Analysis Info

Analysis Name	D:\Data\Monica Rincon\Francisco Cortes Benitez\20231006_6l.d	Acquisition Date	10/6/2023 3:48:46 PM
Method	Tune Low extendido.m	Operator	Admin
Sample Name	20231006_6l	Instrument	micrOTOF 213750.00410
Comment			

Acquisition Parameter

Source Type	ESI	Ion Polarity	Positive	Set Nebulizer	0.5 Bar
Focus	Active			Set Dry Heater	150 °C
Scan Begin	50 m/z	Set Capillary	4500 V	Set Dry Gas	4.0 l/min
Scan End	3000 m/z	Set End Plate Offset	-500 V	Set Divert Valve	Waste

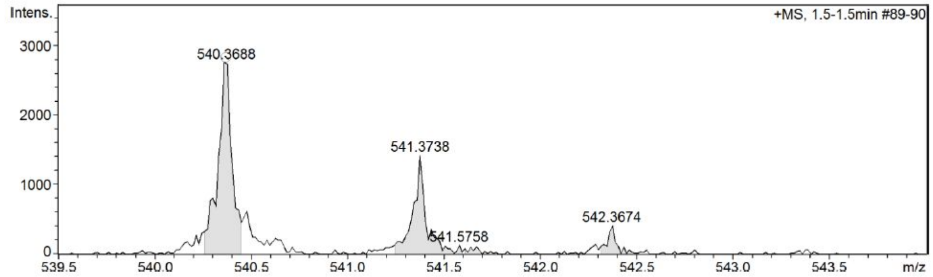

Meas. m/z	#	Ion Formula	Sum Formula	m/z	err [ppm]	|err| [mDa]	mSigma	# mSigma	N-Rule
540.3688	1	C33H50NO5	C33H50NO5	540.3684	-0.9	0.5	83.3	2	ok
	1	C33H50NO5	C33H49NO5	540.3684	-0.9	0.5	83.3	2	ok

Figure S64. Mass spectrum formula report of compound 6l

Mass Spectrum SmartFormula Report

Analysis Info

Analysis Name	D:\Data\Monica Rincon\Francisco Cortes Benitez\20231005_6m.d	Acquisition Date	10/5/2023 4:54:13 PM
Method	Tune Low extendido.m	Operator	Admin
Sample Name	20231005_6m	Instrument	micrOTOF 213750.00410
Comment			

Acquisition Parameter

Source Type	ESI	Ion Polarity	Positive	Set Nebulizer	0.5 Bar
Focus	Active			Set Dry Heater	150 °C
Scan Begin	50 m/z	Set Capillary	4500 V	Set Dry Gas	4.0 l/min
Scan End	3000 m/z	Set End Plate Offset	-500 V	Set Divert Valve	Waste

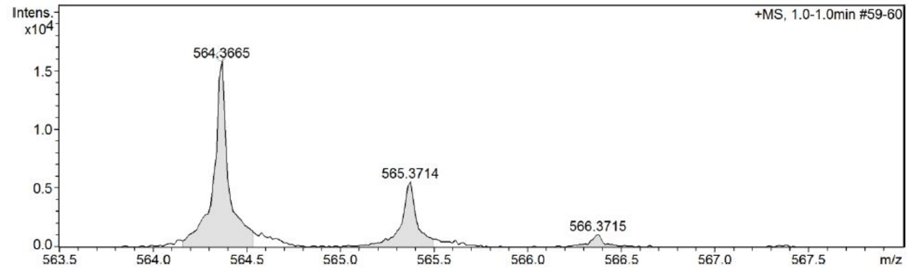

Meas. m/z	#	Ion Formula	Sum Formula	m/z	err [ppm]	|err| [mDa]	mSigma	# mSigma
564.3665	1	C33H49F3NO3	C33H48F3NO3	564.3659	-1.0	0.6	9.3	1

Figure S65. Mass spectrum formula report of compound 6m

Mass Spectrum SmartFormula Report

Analysis Info

Analysis Name	D:\Data\Monica Rincon\Francisco Cortes Benitez\20231005_6n.d	Acquisition Date	10/5/2023 4:19:44 PM
Method	Tune Low extendido.m	Operator	Admin
Sample Name	20231005_6n	Instrument	micrOTOF 213750.00410
Comment			

Acquisition Parameter

Source Type	ESI	Ion Polarity	Positive	Set Nebulizer	0.5 Bar
Focus	Active			Set Dry Heater	150 °C
Scan Begin	50 m/z	Set Capillary	4500 V	Set Dry Gas	4.0 l/min
Scan End	3000 m/z	Set End Plate Offset	-500 V	Set Divert Valve	Waste

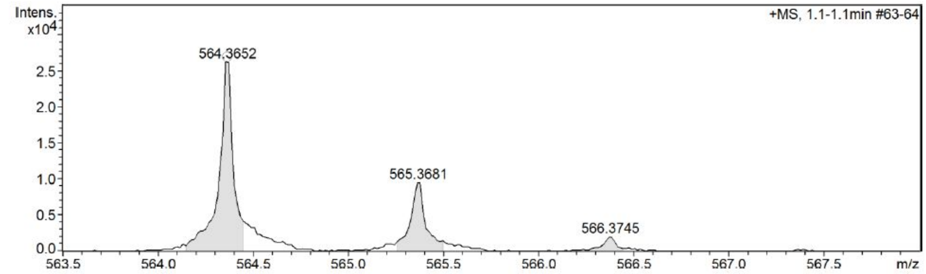

Meas. m/z	#	Ion Formula	Sum Formula	m/z	err [ppm]	|err| [mDa]	mSigma	# mSigma
564.3652	1	C33H49F3NO3	C33H48F3NO3	564.3659	1.3	0.7	2.8	1

Figure S66. Mass spectrum formula report of compound 6n

Mass Spectrum SmartFormula Report

Analysis Info

Analysis Name	D:\Data\Monica Rincon\Francisco Cortes Benitez\20231006_6o.d	Acquisition Date	10/6/2023 2:58:53 PM
Method	Tune Low extendido.m	Operator	Admin
Sample Name	20231006_6o	Instrument	micrOTOF 213750.00410
Comment			

Acquisition Parameter

Source Type	ESI	Ion Polarity	Positive	Set Nebulizer	0.5 Bar
Focus	Active			Set Dry Heater	150 °C
Scan Begin	50 m/z	Set Capillary	4500 V	Set Dry Gas	4.0 l/min
Scan End	3000 m/z	Set End Plate Offset	-500 V	Set Divert Valve	Waste

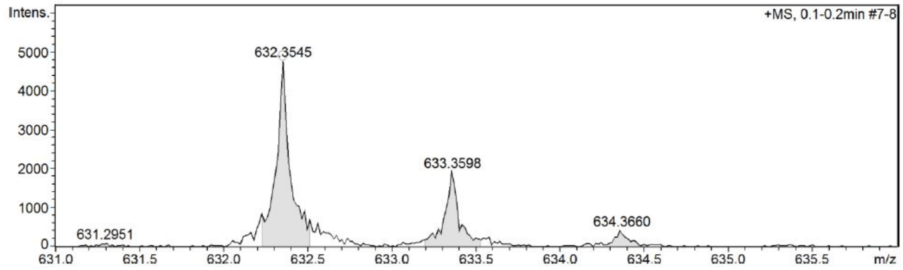

Meas. m/z	#	Ion Formula	Sum Formula	m/z	err [ppm]	|err| [mDa]	mSigma	# mSigma
632.3545	1	C34H48F6NO3	C34H48F6NO3	632.3533	-1.9	1.2	15.8	1
	1	C34H48F6NO3	C34H47F6NO3	632.3533	-1.9	1.2	15.8	1

Figure S67. Mass spectrum formula report of compound 6o

Table S3. Crystal data, structure solution, and refinement parameters for compound **4**

	**4**
Formula	$C_{26}H_{42}O_3$
Molecular weight	402.59
Temperature (K)	293
Crystal size (mm^3)	0.4 × 0.2 × 0.05
Crystal system	Monoclinic
Space group	$P2_1$
a (Å)	10.0725(7)
b (Å)	7.6889(4)
c (Å)	15.2885(10)
β (°)	93.408(6)
Volume ($Å^3$)	1181.95(13)
Z, ρ_{calc} (g/cm^3)	2, 1.131
μ (mm^{-1})	0.072
F(000)	444.0
Radiation	Mo Kα (λ = 0.71073)
2Θ range for data collection (deg)	5.934 to 52.736
Index ranges	$-12 \le h \le 12$, $-9 \le k \le 9$, $-19 \le l \le 19$
Reflections collected	37711
Independent reflections	4831
Data/restraints/parameters	4831/1/266
Goodness-of-fit on F^2	0.893
Final R indexes [I>=2σ (I)]	R_1= 0.0507, wR_2= 0.0977
Final R indexes [all data]	R_1= 0.1297, wR_2= 0.1165
Largest diff. peak/hole (e $Å^{-3}$)	0.10/−0.14
Flack and Hooft parameters	−0.4(10) and 0.2(11)
Inverted Flack and Hooft parameters	1.4(10) and 0.8(11)
CCDC number	2354163

Equation 1 was used to obtained enzyme kinetic parameters by fitting data to the Michaelis Menten model (Origin Pro 2018 (64 bit) SR1)

$$y - \frac{V_{max}\ i}{K_m + i} \qquad \textbf{E1}$$

The equations defined for the following non-linear inhibition models were used to determined the mechanism of PTP1B inhibition: competitive (E2), non-competitive (E3), uncompetitive (E4) and mixed (E5) (Origin Pro 2018 (64 bit) SR1)

$$y = \frac{V_{max}\ (X)}{K_m \left(1+\left(\frac{i}{K_i}\right)\right) + x} \qquad \textbf{E2}$$

$$y = V_{max} \frac{x^{nh}}{\left(1+\left(\frac{i}{K_i}\right)\right)(x_{05} nh) + \left(1+\left(\frac{i}{K_i}\right)\right)(x_{05}\ nh)} \qquad \textbf{E3}$$

$$y = \frac{V_{max}\ (x)}{\left((K_m)/\left(\frac{1+i}{K_I}\right)\right) + x} \qquad \textbf{E4}$$

$$y = \frac{\left((V_{max}\ (x))/\left(\frac{1+i}{\alpha K_I}\right)\right)}{x + K_m\left(\left(\frac{1+i}{K_i}\right)/\left(\frac{1+i}{\alpha K_I}\right)\right)} \qquad \textbf{E5}$$

V_{max} is the maximum velocity, x is the substrate concentration, i is the inhibitor concentration, *nh* is the number of Hill. K_i is the inhibition constant, and K_m is the Michaelis constant.
